# Digital Episodic Future Thinking Intervention (Luminaut): Co-Design and Iterative Development Study

**DOI:** 10.2196/74099

**Published:** 2026-05-06

**Authors:** Naomi Hoffmann, Caitlin A Howlett, Kate Little, Ian Gwilt, Megan A Rebuli, Paige G Brooker, Aaron Davis

**Affiliations:** 1 Commonwealth Scientific and Industrial Research Organisation, PO Box 10041Adelaide, SA, 5000Australia; 2 School of Architecture and Built Environment, Adelaide University, Adelaide, SA, Australia; 3 School of Art and Design, Adelaide University, Adelaide, SA, Australia

**Keywords:** Episodic Future Thinking, digital intervention, future discounting, health behavior change, health behaviors, co-design methods, mobile phone

## Abstract

**Background:**

Digital health interventions can be effective at changing behavior, but achieving long-term adherence remains a challenge. One psychological barrier to health behavior change is *future discounting*, or the tendency to prefer smaller, short-term rewards over larger, long-term rewards. Episodic Future Thinking (EFT) can disrupt future discounting and is a promising technique for improving health behavior, but such interventions have not been co-designed to address end user needs.

**Objective:**

This study aimed to co-design an app with end users to deliver an EFT intervention aimed at promoting health behavior change in those in the prerisk phase for chronic conditions.

**Methods:**

Community members participated in up to 2 series of face-to-face co-design workshops. A prototype of the app was reviewed, and insights were gathered to understand (1) the optimal characteristics of the app and (2) the concepts of future discounting and EFT. Themes were generated using inductive thematic analysis.

**Results:**

Participants were South Australian adults (n=30) who were predominately affluent women (27/30, 90%) aged 25‐44 years (mean 36.37, SD 5.65 years). Feedback generated from the first workshop series resulted in 26 suggestions of which 15 informed iterative app development. Higher-level principles were identified and categorized into 5 overarching themes: concept acceptance, triggers and barriers, personalization, gamification, and user-friendly interface.

**Conclusions:**

This study used co-design methodology to develop an app-based EFT intervention. Ongoing engagement with end users and key stakeholders (eg, health care professionals) is needed to ensure that the app meets changing needs. Future work will aim to evaluate its effectiveness in a large-scale clinical trial.

## Introduction

### Background

Noncommunicable diseases (NCDs), including cardiovascular diseases, diabetes, cancers, and chronic respiratory diseases, account for 75% of deaths each year [1], making them the leading cause of death worldwide [2]. The main risk factors for NCDs are largely preventable and include modifiable lifestyle behaviors such as poor dietary intake, physical inactivity, alcohol consumption, and illicit drug use [3]. Therefore, devising effective lifestyle interventions to reduce NCD risk is an important research priority. The aim of this study was to develop a co-designed smartphone app to deliver a psychological intervention to improve health behaviors in individuals at risk of NCD.

### Challenges in Promoting Sustained Health Behavior Change

Despite the well-established health benefits of consuming a nutritious diet and engaging in regular physical activity [4-6], many people find it challenging to adopt and maintain health-promoting behaviors [7]. Indeed, the 2022 Australian National Health Survey found that only 4.2% of Australians met both the fruit and vegetable intake recommendations, while only 23.9% met the physical activity guidelines [8]. Long-term adherence to lifestyle behavior change interventions is an ongoing challenge [7] and may be partly due to underlying psychological barriers. One such barrier is future discounting, namely, the tendency for individuals to devalue rewards as a function of delayed receipt [9], or prioritize behaviors that provide immediate gratification over longer-term benefits [10]. Higher rates of future discounting have been linked with poor health behaviors and obesity [11,12].

In the context of health behaviors, future discounting involves placing less value on the future by engaging in lifestyle behaviors that produce immediate rewards (eg, sedentary behavior and/or consuming discretionary foods high in salt, sugar, and/or fat). Furthermore, such behaviors often lead to the onset of negative, delayed health consequences (ie, chronic disease) in lieu of long-term future rewards (eg, good health). A preference for smaller, immediate rewards over larger, delayed rewards is a transdiagnostic process that has been implicated in health and disease [13]. Future discounting—or delay discounting as it is otherwise known—is typically measured via monetary or domain-specific (eg, food) choice tasks or questionnaires to assess how quickly rewards of varying amounts reduce in value over time. In the eating domain, a meta-analysis found a medium effect size (*d*=0.43) for the relationship between future discounting for food and monetary rewards and obesity [14]. This suggests that future discounting is a critical target for interventions aiming to modify risky lifestyle behaviors [15].

### Episodic Future Thinking Interventions

Episodic Future Thinking (EFT), namely, a form of prospective thought, involves mentally envisioning oneself experiencing specific, personal, and detailed future-oriented events [16]. EFT is a type of psychological intervention that aims to disrupt one’s tendency to discount the future by altering the perceived value of delayed outcomes [17]. EFT enables individuals to not only mentally experience upcoming events [18] but also consider both the immediate and extended consequences of their actions [19]. Specifically, EFT encourages individuals to consider the potential future consequences of current behaviors that have a detrimental impact on long-term health [12,20].

EFT is based on several components of interventions of behavior change technique (BCT) specified in the behavior change taxonomy [21] and Ontology [22]. These components include those related to goal-directed BCTs (ie, goal setting and comparing goals with current behavior) and increasing awareness of consequences of BCTs (ie, prompt comparative imaging of future outcomes). Nevertheless, it is important to understand why these techniques are effective by examining the mechanisms by which these intervention components change outcome behaviors [23,24]. The proposed mechanisms of action for EFT are a reduction in present bias or future discounting and an increase in long-term prospective thinking and valuation of future rewards [25,26], which fall under the domain of bodily disposition and, in particular, a mental disposition (ie, a temporal orientation toward the future) [27].

EFT interventions involve pre-experiencing positive, specific, vivid, future events that are either planned to occur or could realistically occur—a technique described as a form of “mental time travel” [28-30]. Specifically, it involves imagining *who* one is with, *what* one is doing, *where* one is, and *how* one is feeling. The process is used to generate a set of episodic cues to prompt participants to repeatedly practice visualization of such events. EFT cues have typically been generated using 2 approaches: interview-guided or survey-guided, with cue delivery methods usually involving text, audio, and/or visual formats [31].

Previous research has shown that EFT is effective for reducing future discounting rates. Three recent, large meta-analyses reported medium-sized effects in clinical and nonclinical populations [32-34]. These analyses showed that intervention effects were stronger when the imagined future events were more positive, vivid, and related to the delayed choice, as well as for those individuals with higher choice impulsivity, such as steeper discounting [33]. Specifically, EFT was more effective at promoting healthier decision-making in samples with higher choice impulsivity, which suggests that such training is more effective for people who are less likely to examine the future health consequences of their actions [35]. Thus, choice impulsivity may be a mechanism underpinning the effectiveness of EFT [36,37]. Furthermore, EFT has been shown to reduce the occurrence of a range of risky health behaviors, including cigarette smoking [38]; consuming less (approximately 300 calories) energy-dense, nutrient-poor food during an ad libitum eating task with adults and children with overweight and obesity [39,40]; and decreasing the consumption of alcoholic beverages in people with alcohol dependence [41].

Although EFT is a promising intervention technique, high variability exists in the specific methodology used. Specifically, previous studies have used various amounts of episodic cues generated, timeframes of future events, frequency of engaging with episodic cues, type of cue delivery (eg, text vs audio formats), and whether personal health goals are linked to the episodic cues. Further discrepancies include the control conditions used as comparators (eg, episodic recent thinking, standardized episodic thinking, and health information thinking) and whether the timeframe of episodic cues matches those of discounting time delays on various tasks and questionnaires used to assess such mechanisms of action [31,34]. Furthermore, studies have differed in intervention length and proximity of cue delivery to engaging in the targeted health behavior. Finally, most studies were conducted in the laboratory or face-to-face, which may warrant concerns regarding daily delivery of EFT cues to enable practice of imagery, as well as future intervention scale-up and implementation in real-world settings. While there is currently no standardized protocol for delivering EFT, it is important to address some of these limitations in developing future interventions. Specifically, it is critical to develop EFT interventions that can be scaled up for delivery in the real world and help people to make sense of and apply EFT in their everyday decisions.

More recent EFT studies have used mobile health (mHealth) and other digital technologies to deliver EFT interventions in real-world settings, with a number of studies using smartphones [15,42-44]. Nevertheless, these previous studies involved generating cues in person with a researcher, which were delivered via text or similar prompts to read daily and practice vividly imaging cues. Prior studies did not use smartphone apps to initially develop cues, nor to deliver them daily via in-built notifications. The independent use of a smartphone app also affords participants more autonomy. Studies using immersive environments such as virtual reality to deliver EFT have also emerged and have found promising results for fostering future-oriented thinking [45,46]. Additionally, some previous studies have delivered the EFT cues via an audible format, namely, recordings of participants reading their cues [42,43,47]. A recent pilot study using a smartphone app to develop and create audio-based EFT cues reported that such delivery reduces engagement as participants felt uncomfortable listening to their own voice, and it was practically challenging to find somewhere suitable to listen to such recordings [48]. Thus, it may be more acceptable to deliver cues via written format that could be read out loud if preferred, but it is crucial to engage with end users to determine the most acceptable delivery mode. Importantly, no research to date has developed a user-designed comprehensive digital EFT intervention that enables initial cue generation and notifications to foster daily practice.

### The Benefits of Co-Design

More broadly, one ongoing criticism of smartphone app development and design, including mHealth apps, is the lack of co-design methodology [49]. Co-design leverages insights from people’s lived experiences to foster design-led discovery, innovative ideas, and creative problem-solving, with end users’ unique perspectives, values, and needs being placed at the center of the design process [50,51]. The co-design approach also provides a way to ensure that a product, system, or service is suitable for the people for whom it is intended [52], which could mitigate some of the £100 billion (US $134 billion) per annum in research waste [53,54]. Over the last decade, repeated calls have emerged to adopt more participatory approaches to technology design to improve end user engagement [52,55] and to add more precision when tailoring content and interactions to community and individual needs.

### This Study

Although previous research has used mHealth technology to deliver EFT interventions, no research to date has used co-design to create an engaging digital intervention with potential end users. Furthermore, prior research examining digitally delivered EFT interventions has focused on laboratory-based testing. More broadly, such limitations likely contribute to the lack of uptake, adoption, and scale-up of digital health interventions in real-world settings [56], which adversely impacts health outcomes [57-59]. Using co-design may help address the evidence to practice gap by enhancing adoption by end users, lowering cost and waste, and boosting behavior change by targeting the appropriate enablers [60]. Using co-design is particularly important for mHealth interventions, given it is the most direct technology-consumer link [61]. Nevertheless, high drop-off rates are common due to design issues as end users’ preferences are not often considered [62]. Sustainable use of mHealth interventions could be facilitated using co-design processes [63]. Furthermore, the importance of documenting the co-design process used and how it has influenced the development of digital health intervention is necessary, given the current lack of standardized measures to assess impact [64] and the inconsistent reporting to date regarding specifics of the co-design sessions [65].

Thus, the purpose of this research was to co-design a smartphone app prototype of an EFT intervention, namely, “Luminaut.” The app aims to support individuals with their health-related behavior change goals via EFT by drawing on the perspectives of adults in the “prerisk” phase for chronic health conditions. The “prerisk” phase refers to the stage before risk factors for chronic conditions emerge whereby individuals are still relatively healthy but aiming to prevent the development of chronic health conditions. Specifically, this study used an iterative co-design process to (1) understand the optimal characteristics and components (eg, content, format, and delivery) of the *Luminaut* app based on the perspectives of potential end users, and (2) gather end users’ feedback based on interacting with the *Luminaut* app prototype.

## Methods

### Overview

This study used patient and public involvement, defined as research designed and conducted *with* or *by* members of the public rather than *to*, *about*, or *for* them [66]. Specifically, we used a co-design approach based on the British Design Council’s Double Diamond Design Process—a framework that structures the design process into 4 stages: Discover, Define, Develop, and Deliver [67]. Co-design was chosen for its participatory nature, engaging users and stakeholders throughout the process [68]. The Double Diamond framework uses an iterative, user-centered process and involves collaboration between stakeholders to revisit and refine design solutions based on emerging insights and continuous feedback. Gero and Milovanovic’s [69] Situated Function-Behaviour-Structure was used as an analytical framework as it provides a dynamic model for understanding how design evolves as a result of collaborative interactions, feedback, and ongoing refinement through the various stages of co-design. Using this lens, feedback, and decision-making among participants including users, researchers, and designers can help actively shape the development of the design, ensuring that it remains responsive to user needs and emerging insights.

### Ethical Considerations

This study was approved by the Commonwealth Scientific and Industrial Research Organisation Human Research Ethics Committee (ID 2023_055_LR). All procedures were conducted in accordance with the ethical standards of the responsible committee on human experimentation (institutional and national) and with the Declaration of Helsinki (1975), as revised in 2000. We adhered to the GRIPP2-SF (Guidance for Reporting Involvement of Patients and Public Short Form) reporting checklist, which is used for reporting the involvement of people with lived experience in research [66]. To maintain participant privacy, the authors deidentified any personal information contained in the data and results so that individual participants cannot be identified.

### Participants and Sample Size

Participants were adults aged 25-44 years. This age group was selected as individuals in this age group are considered to be in the “prerisk” phase for chronic health conditions [70] and are more likely to adopt and use health apps [71]. In line with co-design conventions, we aimed to recruit a minimum of 8 participants per co-design workshop, with no more than 20‐25 participants in a single workshop and up to a maximum total sample size of 36. Sample size calculations for co-design research are focused on accessing the viewpoints of different stakeholder groups, the development of saturation of insights, and deep engagement regarding the research questions, rather than focusing on statistical significance [72,73]. In many generative co-design practices, the focus is on accessing tacit and latent knowledge and insights through exploratory practices, rather than on accessing explicit knowledge, behavioral data, or generalizable “facts” [74].

### Co-Design Facilitator Team

The co-design project team comprised a research team including 2 behavioral scientists, a nutrition scientist, and a research dietitian who was also the study coordinator, along with a team of 3 design researchers with expertise in co-designing for health.

### Study Design

**Figure 1.**
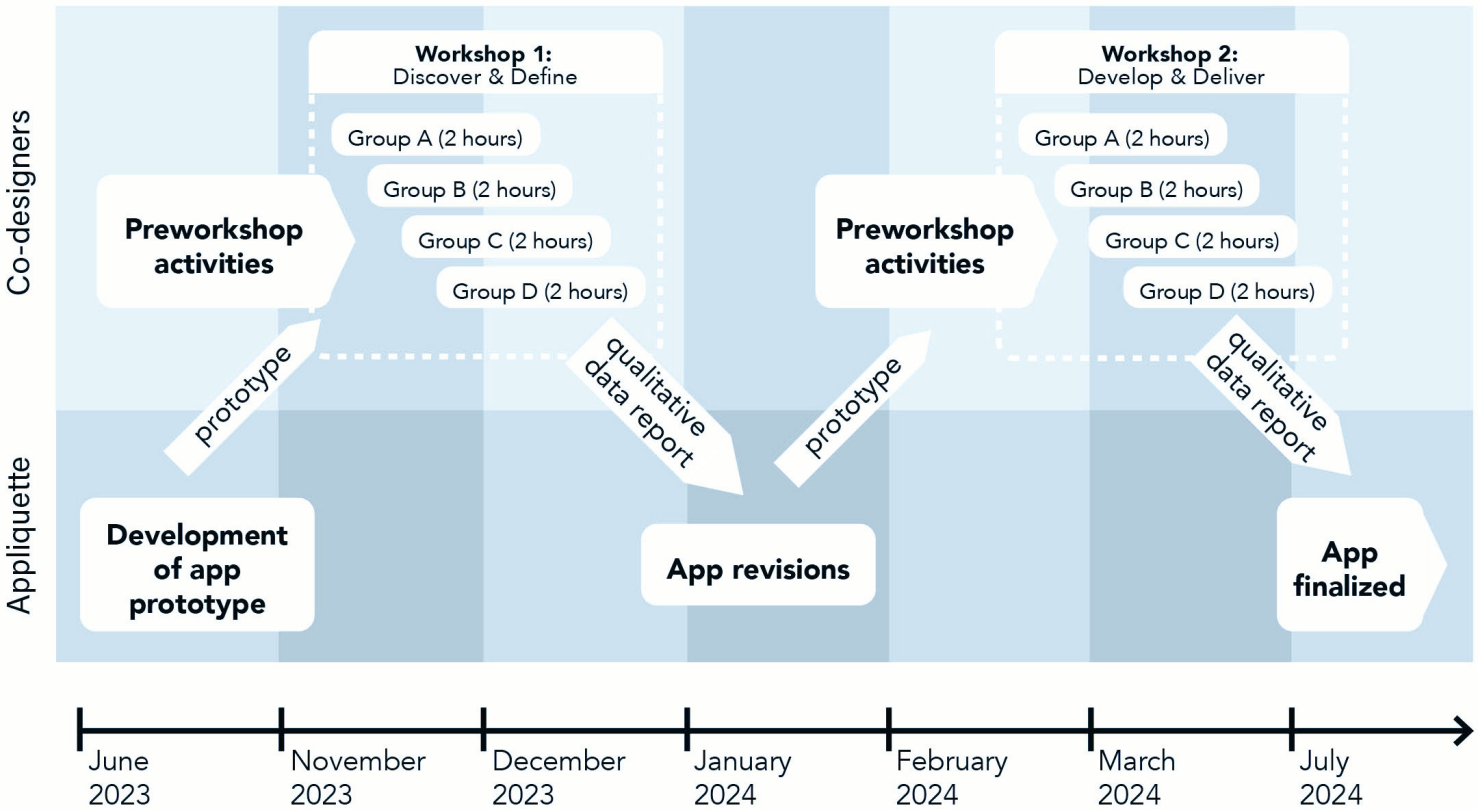
Schematic representation of the study design.

An overview of the study design is illustrated in Figure 1. Briefly, a prototype of the Luminaut mobile app was developed by a third-party app developer (Appliquette Pty Ltd). Once a prototype of the app was developed, participants installed the app on their personal mobile devices and provided feedback via a face-to-face workshop (“workshop 1”). Revisions to the mobile app were then made and presented to participants in a second workshop (“workshop 2”). The app was revised a second and final time to reflect any additional feedback raised.

### Development of the “Luminaut” Mobile App Prototype

Before commencing engagement with the co-design facilitators, the researchers worked directly with Appliquette to develop an app prototype as a starting point for the design challenge that translated the mechanics of the principles of Future Discounting into an app-based experience. This iterative prototyping approach is commonly used in user experience design and provided a shared starting point of (immediate) past experience for the workshop, rather than relying on having had experience with other analogous smartphone apps [75].

A schematic depiction of the app was developed using mood boards in a collaborative interface design tool, Figma, as a way for Appliquette to develop iterative prototypes and collect feedback from the researchers to satisfy the functional requirements of the app. Figma was used to cocreate and illustrate the page layout and order, content (eg, formatting and arrangement), functionality (eg, help and back buttons), images and animations, and different interface displays. The app was pilot-tested by a minimum of 2 researchers during all stages of development. The content of the app is outlined in Textbox 1. Of note, participants can be allocated to complete all or any combination of these app components but were asked to complete all components in this study.

Textbox 1. **Luminaut app components and specific activities.**

Health Goals

- Users selected a health domain (ie, physical activity or dietary behavior).
- Users created 3 health goals (related to the chosen health domain) that were achievable within 3 predetermined timeframes (3, 6, and 12 months).
- Users rated how achievable and important their goals were (1=very achievable or unimportant to 5=very achievable or important).
- Users who rated health goals as 3 or less were asked to regenerate a goal that was important and achievable.

Future Events

- Users described 3 future events that they have planned or anticipate could take place within 3 predetermined timeframes (3, 6, and 12 months).
- Each event had to be positive, vivid, and described using present tense, and must occur within a 24-hour timeframe.
- Users rated how enjoyable, important, exciting, and vivid each event is (1=very achievable or unimportant to 5=very achievable or important).

Episodic Cues

- Combine health goals with each positive future events to form cues delivered via daily in-built notifications (personalized).

Ecological Momentary Assessment

- Questions to capture momentary mood and emotions included

- Fatigue
- Sleepiness
- Alertness
- Energy

### Procedure

Participants were recruited through paid advertisements on Facebook and Instagram (Meta). Interested individuals were directed to complete a lead generation form, which prompted them to enter their name and preferred email address. Those who expressed interest in participating were emailed a brief online questionnaire hosted in REDCap (Research Electronic Data Capture; Vanderbilt University, 2004), which included an electronic participant information sheet and consent form that outlined the study inclusion and exclusion criteria. Individuals were required to tick a checkbox to indicate electronic informed consent. Those who provided informed consent were directed to a set of questions to determine eligibility, dietary requirements, availability (ie, date and time), and preferred workshop location. Those who completed the initial questionnaire were assigned a unique identifier code, which was generated in REDCap. To be eligible to participate, individuals needed to be (1) aged between 25 and 44 years, (2) fluent in the English language, (3) have access to an internet-connected smartphone device (iOS or Android) that has the minimum required operating system (ie, iOS11 or Android 6), (4) willing and able to install a free prototype of the “Future Self 4 Health” smartphone app on their device, and (5) able to attend up to 2 face-to-face workshops held in a metropolitan area of Adelaide, South Australia. Individuals with a self-reported diagnosis of aphantasia, namely, the inability to imagine or visualize or create mental images, were not eligible to participate. Of note, the app was originally called “Future Self for Health” and was then later changed to *Luminaut* following feedback obtained during the co-design process (for more information, see Multimedia Appendix 1).

Those deemed eligible were provided a unique link to complete an online survey to collect demographic information (ie, age, gender, education level, fluent speaker of languages other than English, health conditions, postcode, and smartphone device) hosted in REDCap. Participants were then contacted by the research coordinator and sent a follow-up email containing preworkshop activities and specific details about the face-to-face workshops. Participants received an honorarium in the form of an e-gift card up to the value of Aus $100 (US $71.48) for each workshop that they attended.

### Preworkshop Activities

The *Luminaut* prototype app was free for participants to download via the App Store (Apple) or Google Play Store (Android) using beta-testing links (ie, TestFlight). Prior to workshop 1, participants were emailed instructions about how to install and login to the app and information concerning their allocated workshop (ie, date, time, and venue), as well as an infographic to familiarize participants with the concept of future discounting. Participants received a link to download the app prototype and were asked to test its functionality and features over approximately a 1-week period.

### Co-Design Workshops

A series of 8 workshops (4 iterations of workshop 1, and 4 iterations of workshop 2) were conducted between November 2023 and March 2024. Participation involved completing up to two, 2-hour face-to-face workshops. Co-design workshops were facilitated through Match Studio (a creative research laboratory at the University of South Australia). To increase participant diversity, a series of workshop times and locations were offered, with the aim to recruit a sample of individuals with different levels of socioeconomic advantage. The locations chosen encompassed Local Government Areas (LGA) with both high and low rankings according to the Australian Index of Relative Socio-economic Advantage and Disadvantage (IRSAD) [76]. The two locations in South Australia were (1) the University of South Australia City West Campus, Adelaide (LGA IRSAD quintile 5); and (2) the Christie Downs Community House, Christies Downs (LGA IRSAD quintile 3) [76]. Two time slots were offered: daytime (10:30 AM to 12:30 PM) or evening (5:30 PM to 7:30 PM).

In response to participant preferences, most workshops (6/8, 75% comprising 4 daytime and 2 evening) were facilitated at the University of South Australia City West Campus as well as 2 daytime workshops at the Christie Downs Community House. The workshops used a combination of participant-led documentation and recording during the sessions. Where small group verbal sharing took place, these parts of the workshops were recorded using Dictaphones (Panasonic RR-US065, Olympus VN-731PC, and Sony R41174041), while facilitators also took field notes to aid in transcription and thematic analysis. Light refreshments were offered to participants throughout as a way of offering additional value to participants and providing an opportunity to invert power relationships between the facilitators (serving) and participants (receiving).

Workshops were informed by previous studies and iteratively by findings arising from each co-design session, in accordance with the Design Council’s Double Diamond Design Process framework (ie, Discover, Define, Develop, and Deliver), which combines both divergent and convergent approaches [67]. The first workshop was designed to engage with the “Discover” and “Define” phases of the Double Diamond, using creative activities to help participants access and share tacit and latent, as well as explicit, knowledge [74]. Activities involved facilitated discussions and participant-led documentation (worksheets, low-fidelity prototypes, and card-sorting activities). A timeline of activities during workshop 1 is provided in Table 1, with a longer description of each workshop contained in Multimedia Appendix 2.

**Table 1.** Timeline of activities during workshop 1.

Time, minutes	Arrival
5	Opening remarks: Acknowledgment of Country, purpose and objectives of the workshop
15	Future discounting introduction and icebreaker
30	Activity 1: Card sort: What is and what isn’t future discounting
25	Activity 2: Where and when activity stories
10	Break
30	Activity 3: App inventory exercise
30	Activity 4: Specific app test feedback
5	Wrap up
	Close

Following the completion of each instance of this workshop, insights were synthesized to clarify the app’s intended function, the behaviors it aimed to support, and to refine the design objectives. Across the workshop 1 series (ie, 4 workshops), small changes were made to the length of time spent on each of the activities to respond to the experiences and interests of the co-design participants. At the completion of the 4 instances of this workshop, the data were analyzed by AD and KL using inductive thematic analysis [77]. The analysis was then translated into an interim report that was shared with the researchers who reviewed the collated feedback in collaboration with AD and KL. The researchers devised a list of changes to be made to the app. Appliquette Pty Ltd addressed and implemented the changes, which were categorized as “high,” “medium,” and “low” priority, and released an updated version of the app to participants.

A few weeks prior to workshop 2, participants were given the opportunity to test the revised version of the app (over approximately a 1-week period) and were asked to provide feedback and reflections via an anonymous online survey, hosted via the Qualtrics survey platform (Qualtrics). This survey was designed to prompt open-ended reflection while also asking participants to compare what they had been testing with the original prototype. Participant contributions to this survey were analyzed through an inductive thematic analysis process and were used to shape the design and focus of workshop 2, which engaged the “Develop” and “Deliver” phases of the Double Diamond process. A timeline of workshop 2 is provided in Table 2.

**Table 2.** Timeline of activities during workshop 2.

Time, minutes	Arrival
5	Opening remarks: Acknowledgment of country, purpose, and objectives of the workshop
5	Future discounting introduction: Recap from previous workshop
20	Activity 1: Explaining future discounting to a friend using storyboard template and noun project icons
10	Activity 2: Development of sorting game activity based on selected icons
(10)	Optional activity: Financial and other barriers to achieving goals. Mapping example icons and types of visualizations to financial work, family, stress, etc, barriers
10	Break
15	Activity 4: Metaphors and dashboards review Avatars, tree growing, looking through portal, adjusting a date, advent calendar, road, stepping stones/islands, seasons, graphs/abstract data visualization, list
40	Activity 5**:** Specific feedback on current app state What milestones give a badge? How much difference does a photo make? What if it is a stock image? (Use cards from first workshop + icon cards)
5	Wrap up
	Close

### Thematic Analysis

Involvement in the co-design workshops allowed researchers to immerse themselves in the data. The reflexive thematic analysis framework established by Braun and Clarke [77] was used for inductive coding, namely, generating codes directly from the data rather than from preexisting categories. Following each workshop, all data, including participant-led documentation, researcher field notes, transcribed audio recordings, and survey responses, were reviewed to identify key themes and patterns and coded using NVivo (QSR International) and Microsoft software (Microsoft Inc, ie, Excel and Word).

Data from each of the 9 activities across the workshops (workshop 1 series=4 activities, workshop 2 series=5 activities) were reviewed independently by the co-design facilitators after each iteration of the workshop to ensure that the workshops were effectively contributing to the ongoing development process. The co-design facilitators made small changes, such as adjusting the time spent on different activities, to ensure that all intended explorations in the workshop were undertaken across the workshop series. Initial themes were also reviewed by the co-design facilitators to develop a rich understanding of the data following Braun and Clarke’s [78] recent guidance on quality practice in reflexive thematic analysis at the end of the workshop series to develop recommendations and overarching themes.

Across the series of co-design workshops, the process of theme development involved multiple cycles of coding, categorizing, and revising. Themes were reassessed at each stage to ensure that they accurately captured the core patterns in the data, with codes merged, split, or redefined as necessary. This iterative approach enabled the identification of key themes that emerged as most relevant to the area of enquiry [77]. A final report was then generated by the co-design team for the nutrition and behavioral scientists (Multimedia Appendix 3). The report included high-level insights from the co-design process, a description of the workshop structure and activities, interim recommendations following workshop 1 series, data from the asynchronous feedback process, and data from both workshop series.

## Results

### Participant Recruitment

**Figure 2.**
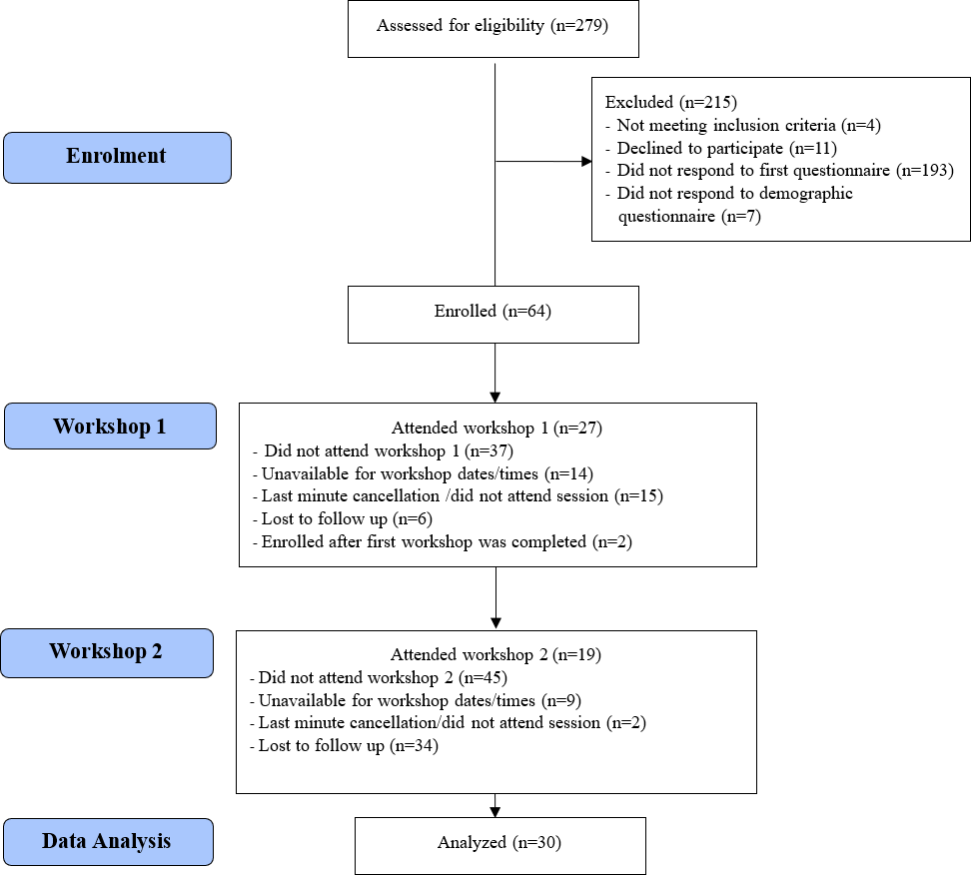
Participant flow diagram.

A total of 279 individuals expressed interest in participating in the study via the social media (Facebook and Instagram) advertisements. Of these, 71 individuals provided informed consent, completed the follow-up questionnaire, and met the study eligibility criteria, following which 64 (90%) individuals completed the demographic questionnaire and were enrolled into the study. Eligible participants were contacted by the research coordinator (MR) to confirm their availability to participate. A total of 30 (47%) participants attended at least 1 workshop, 16 (25%) participants completed both workshops, 11 (17%) participants completed the first workshop only, and 3 (5%) participants completed the second workshop only (Figure 2).

### Sample Characteristics

The characteristics of the participants who attended workshops are shown in Table 3. Participants were mostly women aged 25‐44 years with a bachelor or postgraduate university degree, who owned an Apple smartphone device, had relatively high socioeconomic advantage in accordance with the Australian Bureau of Statistics Socio-Economic Indexes for Areas, Australia [76], and lived with no or 1 chronic health condition. The characteristics of all participants who were initially enrolled into the study are provided in Multimedia Appendix 4.

**Table 3.** Characteristics of participants in workshops 1 and 2 as well as the total sample.

Characteristic	Workshop 1	Workshop 2	All attendees
Total, N	27	19	30
Female, n (%)	24 (88.89)	17 (89.47)	27 (90)
Age (years), mean (SD)	36.74 (5.52)	37.37 (4.96)	36.37 (5.65)
Age range (years)	25‐44	28‐44	25‐44
Education, n (%)			
Secondary education (year 12)	—^a^	—	—
Certificate III and IV level	2 (7.41)	—	2 (6.67)
Advanced diploma/diploma level	1 (3.70)	—	1 (3.33)
Bachelor degree level	8 (29.63)	9 (47.37)	11 (36.67)
Graduate diploma/certificate level	7 (25.93)	4 (21.05)	7 (23.33)
Postgraduate degree level	9 (33.33)	6 (31.58)	9 (30)
IRSAD^b^ quintiles, n (%)			
1 (most disadvantaged)	1 (3.70)	1 (5.26)	1 (3.33)
2	1 (3.70)	—	1 (3.33)
3	8 (29.63)	5 (26.32)	9 (30)
4	4 (14.81)	4 (21.05)	5 (16.67)
5 (most advantaged)	13 (48.15)	9 (47.37)	14 (43)
Fluent in languages other than English, n (%)	10 (37.04)	5 (26.32)	11 (36.67)
Number of chronic health conditions, n (%)			
0	11 (40.74)	10 (52.63)	14 (46.67)
1	12 (44.44)	6 (31.58)	12 (40)
2	2 (7.41)	2 (10.53)	2 (6.67)
3	2 (7.41)	1 (5.26)	2 (6.67)
Smartphone device type, n (%)			
Apple	16 (59.26)	11 (57.89)	18 (60)
Android	11 (40.74)	8 (42.11)	12 (40)

a Not available.

b IRSAD: Index of Relative Socio-economic Advantage and Disadvantage—SEIFA Postal Area (POA) [76].

### Specific App Feedback From Co-Design Engagement

At the end of the first workshop series, 26 suggestions for changes to the *Luminaut* app were identified from the data (Multimedia Appendix 3). These suggestions ranged from high-level conceptual suggestions (eg, shifting “language to be more positive rather than the negative framing of ‘future discounting’”) to specific feedback (eg, “lock or add friction to reentering quiz after completion to avoid looping”). The research team recommended implementing 16 of these suggestions within the app, which were categorized as high (n=8), medium (n=6), or low (n=2) priority. A high priority suggestion included adding in clearer instructions and screenshots that showed a preview of how to navigate the app, while a medium priority suggestion included using images to represent goals (icons or stock images) and a low priority suggestion included enabling push notifications that could be customizable based on personal preferences by asking participants to nominate suitable times of day practice visual imagery using the cues. Practical considerations including time and budget were the primary reasons for not implementing all user suggestions at this stage; however, these suggestions were noted for future possible versions of the app. An additional 4 suggestions were identified as needing further exploration and bespoke activities were developed to explore these ideas in the second series of workshops. These suggestions mostly included exploring an introduction narrative or ways to introduce the concept of “future discounting” quickly, as well as opportunities for various visual metaphors to be used as a structure for the app dashboard.

**Figure 3.**
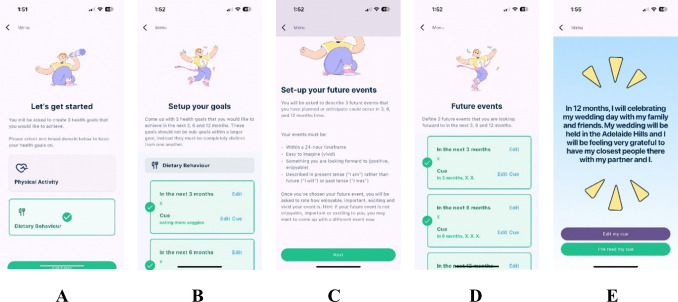
. Design of the Episodic Future Thinking (EFT) Luminaut smartphone app. (A) The screen where participants select a health domain (physical activity or dietary behavior); (B) the instruction screen for setting up health goals for the 3 timepoints for the selected health domain; (C) the instruction screen for setting up the future events; (D) the home screen for the 3 timepoints for the future events; and (E) an example of an EFT cue delivered to participants daily to assist in practicing visual imagery.

During the second workshop series, participants provided further feedback on the app design (see Figures 3A-3E for screenshots of the updated app prototype). Specifically, they provided specific feedback on the experience of using the updated prototype, while also exploring the higher-level conceptual challenges identified during the first workshop series. This dual focus enabled the co-design process to explore ideas that could reasonably be integrated and explored in this project and were appropriate to the project stage, while also contributing to strategic opportunities for future research. Key feedback on the app included that there were “too many notifications” and exploring whether sentence starters or generative artificial intelligence could be integrated to help participants create realistic, yet aspirational health goals. Facilitators reflected that there was a tension between viewing the app as a goal setting and achievement app and understanding the principles of catalyzing subtle behavior shifts away from future discounting; in effect, a microexample of the challenge of future discounting itself.

A total of 8 narrative elements were identified for use within the app. The most common was variations of “make good choices now to enjoy the benefits later,” which mirrored the introductory content presented to participants. However, a novel conception also emerged with “planting a seed and nurturing a plant” providing a metaphor that is positive in both the present and future framings. Preference for navigational metaphors also followed this biophilic concept with growth and trees identified as the most preferred way of arranging information. Metaphors that were identified as inappropriate tended to separate time into discrete moments rather than a continuum, including a portal to a future time, moving between islands, and jumping seasons.

### Insights Into Future EFY Intervention Design

#### Overview

As well as delivering specific feedback that could inform the ongoing app development, the project also enabled the development of higher-level principles and learnings that can be widely applied in similar projects and systems using EFT interventions. Five overarching themes were identified, which were each grounded in the lived experiences of participants and integral to the design decisions made during the project. The five themes were (1) Perceived Value, Understanding, and Trust in Shaping Acceptance of the EFT Concept; (2) Factors Influencing Engagement: Triggers and Barriers; (3) The Role of Personalization in Enhancing User Experience; (4) Game Mechanics as Drivers of User Motivation; and (5) The Importance of Usability in Optimizing User Experience.

#### Perceived Value, Understanding, and Trust in Shaping Acceptance of the EFT Concept

Concept acceptance encompassed participants’ understanding and trust in the underlying premise of the app. While many participants recognized the app’s potential to facilitate behavior change, there was notable confusion regarding some aspects of the intervention, including its functionalities and intended outcomes. One participant remarked that, “[It needs] some explanation or context to help know what to do (e.g., infographic)” and another requested, “...guidance about how to set a goal and how to achieve it. ‘Help me figure it out.’” This underscored the necessity for clearer communication about how the app would support users in their health journeys, particularly highlighting the importance of an introductory guide to convey this information effectively.

Trust also emerged as a crucial factor with participants indicating that the app’s credibility and the reliability of its recommendations were essential for their ongoing use and for considering recommending it to others. The participants sought to use an app from a brand they could trust. They also sought evidence of scientific foundations and anecdotal evidence or direct experience of successful outcomes from using the app. One participant said, “I have to see the complete product, finished, use it for a bit and see that it is working for me.” Others remarked that they would need to see “scientific evidence” or have the app “recommended by a psychologist.”

The concept of EFT is grounded in scientific evidence; however, communicating this effectively is challenging due to the complexity of the psychological underpinnings, a challenge that participants identified. This theme was explored iteratively throughout the co-design process, with potential solutions collaboratively developed during the co-design sessions to enhance clarity and understanding.

#### Factors Influencing Engagement: Triggers and Barriers

Participants identified various triggers that could influence their consideration of using the app, such as hearing about the illness of a friend or family member, health events, or personal milestones. These triggers could serve as motivators to engage with the app, prompting users to seek support for their health journeys. Examples include, “Family member diagnosed with illness, eg, diabetes,” “Bad news from the GP,” and “Change in location/moving.”

Barriers were also identified that could hinder their engagement. These included competing priorities in daily life (“What are all the other things that I should be doing?”), time and effort required to use the app (“Effort, mental effort”), aversion to constraint (“You don’t want to be the type of person who denies themselves”), difficulty navigating unfamiliar or complex tasks (“Inability to prioritise can be a big barrier, that overwhelm feeling”), technological illiteracy (“Some people don’t even know how to install an app”), and financial cost (“Healthy food is expensive”). Understanding the interplay between triggers and barriers can help inform design decisions, ensuring that the app effectively supports users in overcoming obstacles while leveraging motivating factors to encourage sustained use.

#### The Role of Personalization in Enhancing User Experience

The theme of personalization emerged prominently, with participants emphasizing the significance of tailoring the app experience to individual needs and preferences. Many expressed a desire for customizable features, including editable goals, the ability to change notifications (time and frequency), and options to select avatars and images that reflect their personal preferences and circumstances. For example, one participant requested the “[o]ption to switch off [notifications]. [I’m] trying hard to reduce phone usage, for health.” Participants indicated that a personalized approach could foster a deeper connection to the app, which may ultimately enhance motivation and adherence. This insight informed design decisions, resulting in the inclusion of flexible options.

#### Game Mechanics as Drivers of User Motivation

##### Overview

Participants showed enthusiasm for gamification elements within the app, believing that game-like features could enhance user engagement and make the experience more enjoyable. Suggestions included reward systems, challenges, social sharing capabilities, progress tracking, and streak tracking. Participants noted that incorporating these elements could transform engagement with the app into a fun and interactive experience, fostering community support and accountability, while offering rewards that reinforced positive behavior. Integration of gamified components designed to encourage sustained interaction and facilitate progress tracking is planned for future iterations of the app. The integration of these gamification elements is expected to create a more engaging and motivating user experience.

##### Reward Systems

Several participants suggested implementing a reward system, where users could earn rewards for completing tasks. For example, “[I] like goal setting aspects...and small rewards in-app for progressing.” Suggested rewards included frequent flyer points, financial credit, vouchers, and in-app rewards (eg, badges). One participant mentioned that the app’s use of positive language feels rewarding, remarking that, “Positive language is itself a reward. Maybe you don’t need [other] rewards.”

##### Challenges

Participants expressed interest in participating in challenges. One participant stated, “I like to enter competitions.”

##### Social Sharing

The potential for social sharing features was also highlighted. Multiple participants contributed, with one remarking that, “Connecting with friends is a big one.[it’s] encouraging and inspiring to see other people, including family and friends,” and another commented, “I like that functionality, where you can give someone a virtual high five. Celebrate the fact that they reached the 100-day streak or whatever.”

##### Progress Tracking

The ability to track progress through gamified elements was noted as a key feature, to record and present information for direct feedback and tracking over time, useful for monitoring progress toward personal goals. During the discussion, participants expressed their views: one noted, “[I like to] see information at a glance…[to] check if I'm improving,” and another remarked, “Tracking...a way of showing in the app, this is where I started, and this is where I got to...”

##### Streak Tracking

The concept of tracking streaks was particularly popular among participants. One participant stated, “Streaks are motivating.”

### The Importance of Usability in Optimizing User Experience

Participants were prompted and encouraged to provide feedback on their lived experiences with the app. Bugs and glitches were identified and addressed throughout the process. Participants emphasized that an easy-to-navigate interface is essential for ensuring engagement and accessibility. Feedback underscored the need for simplicity and intuitiveness in app design, highlighting the importance of clear instructions, straightforward layouts, and visually appealing design elements. By prioritizing usability, there is an opportunity to create an engaging environment that fosters positive interactions with the app.

## Discussion

### Principal Findings

This study involved co-designing a digital health intervention, “Luminaut,” which is aimed at disrupting future discounting via EFT in individuals at risk of developing chronic health issues. The co-design approach that was used generated app-specific feedback for technical improvements and led to high-level conceptual suggestions that helped shape the Luminaut intervention. This study revealed 5 high-level conceptual suggestions that were important to the participants when engaging with digital EFT interventions such as the Luminaut app. These themes included concept acceptance, triggers and barriers, personalization, gamification, and a user-friendly interface. Overall, the co-design approach was integral to shaping the Luminaut app and it also provides insights into EFT interventions more broadly regardless of delivery mode.

The first theme identified was that *concept acceptance* of future discounting may promote participants’ understanding and trust in the underlying premise of the app. The co-design process provided valuable insights into how participants receive the concept of future discounting in the context of app creation and how they would like to interact with it. Specifically, the high-level conceptual suggestions related to this theme included the overarching challenge of engaging with the concept of future discounting. The concept of future discounting was perceived as being primarily negative in nature and it was suggested that more positive framing of the concept such as “future investing” be used when describing the intervention to participants, which should be considered when delivering EFT or other psychological interventions aimed at reducing future discounting. While it was relatively straightforward to include an explanation of the term “future discounting” into subsequent versions of the Luminaut app, it prompts further consideration of lay understandings of complex psychological phenomena. These contextual insights may elude formal assessments of efficacy, such as randomized controlled trials, which are necessarily more structured and less flexible; however, when combined, they may offer complementary insights at different stages of the research. Through the open, iterative, and creative process, participants shared their evolving understanding and active engagement with the concept, rather than simply providing feedback. By sharing their learning journey, participants revealed both the process of understanding and what they already know about the future discounting concept based on their prior experiences.

In addition, it was found that there are specific *triggers and barriers* that motivate or hinder individuals’ engagement with the Luminaut app. Specifically, the participants identified that the recommendation of the app by a health care professional (eg, a psychologist) would boost trust, which expands on previous co-design research indicating that participants would be more willing to use an app if it was prescribed [79]. Such findings support the idea of using such apps as a way to empower individuals who are ready to take action toward health-related behavior change, which is consistent with the findings of previous co-design work in participants living with chronic health conditions [80].

The finding that participants valued *personalization* based on individual needs and preferences aligns with prior research indicating that profiling, tailoring, and autonomy are important when engaging individuals with digital health solutions for chronic disease prevention or management [79,81,82]. Taken together, such findings are a critical reminder that there is no “one-size-fits-all” approach when it comes to health care and that creating digital technologies that align with individual needs is a promising strategy for health behavior change [83]. Indeed, an advantage of the co-design process is its ability to identify diverse collectively agreed factors that may enhance engagement with such interventions to promote health behavior change.

In addition, elements of *gamification* including reward systems, challenges, social sharing, progress tracking, and streak tracking were also mentioned as features that could elicit a more enjoyable experience while using the app. The latter finding supports previous work showing that apps of gamified features in nongame settings, particularly in digital interventions for health and chronic conditions, are growing rapidly and may enhance the perceived experiences of users [84]. Future work is needed to determine whether the inclusion of such elements has benefits beyond boosting short-term engagement as indicated in recent systematic reviews [85].

Finally, a *user-friendly interface* was encouraged to ensure engagement and accessibility. The latter speaks to the idea of ensuring that digital interventions have high levels of usability, which may be enhanced via incorporating simple features (eg, a back button) and should be systematically evaluated such as via the System Usability Scale score [86].

### Learnings From the Co-Design Process

One of the key learnings from the co-design process included the need to iteratively develop the workshop content based on participant feedback. Namely, the co-design process involved not only engaging with end users to co-design the product (ie, the Luminaut app) but the research process itself. For example, the co-design workshops involved exploring how the concept of future discounting could be communicated quickly and effectively, and the development of an overarching navigational metaphor for engaging with the future. A key piece of feedback from the first series of workshops was that the way the co-design facilitators explained the concept of future discounting made the whole project “make sense.” Thus, in the second series of workshops, the facilitators constructed an activity using a storyboarding template to focus on how participants might explain the concept of future discounting to their friends.

In addition, our multidisciplinary approach provided both challenges and benefits, which is common when using such approaches [87]. For example, there were discipline-based differences regarding conceptualizations of bias in research whereby co-design practitioners consider health researchers a key stakeholder to include as participants in co-design workshops. In contrast, the nutrition and behavioral health scientists viewed researcher input as a potential source of bias in such settings, given their status as “experts” in their given field. In this case, it was deemed useful to have one of the content experts (ie, researchers) present at each workshop as an observer and to answer any content-specific questions if they arose from participants.

### Strengths and Limitations

This study had several strengths and limitations. While co-design studies often focus on involving end users in the conceptualization of research questions prior to any research taking place, this study was focused on exploring how access to support for overcoming future discounting behaviors might be scaled through digital technologies. The requirement of the research funding to develop a digital intervention served as a limitation; however, the advanced prototyping that was enabled by the imposition of this constraint enabled richer explorations of how technology mediated health interactions to take place. Indeed, other similar research aimed at developing digital health interventions has also used prototypes (ie, a website) as it provides stakeholders with a tangible artifact to engage with during the iterative testing process [88]. In this study, as the app design progressed, findings from the “Develop” and “Deliver” phases were used to refine earlier stages, ensuring that the design remained responsive to user needs and stakeholder input. This iterative process ensured that the design was continuously aligned with evolving insights. Nevertheless, it should be noted that this approach does necessitate the upfront allocation of time and money to support this iterative, stakeholder-engaged process, albeit the reward is a more sustainable and appropriate intervention in the medium to long term.

Although our sample was relatively large, which was a clear strength, the sample was subject to several limitations, such as comprising mostly female participants who spoke English fluently with high levels of education. Participants also had a relatively advantaged socioeconomic status, despite attempts to promote diversity through holding workshops in disadvantaged areas (eg, in the outer southern areas of Adelaide). Thus, the Luminaut app may be more tailored toward affluent women in terms of the look, feel, and technological familiarity. Of note, the lack of a representative sample in this study is consistent with previous work and remains an ongoing challenge in health co-design research [80]. Nevertheless, others have argued that as qualitative research relies on purposive sampling methods, there is less emphasis placed on achieving statistical generalizability than there would otherwise be when using quantitative methods [89].

Another limitation is that the 2 selected workshop locations may have excluded those individuals living in rural and remote areas and/or without readily available access to technology such as smartphone devices, which may have further contributed to the “digital health divide” based on an individual’s social status [90]. Indeed, individuals with low socioeconomic status as well as older adults and ethnic minorities typically have lower digital literary and more difficulty accessing digital health interventions [91]. Such accessibility issues may be addressed in future research using non–digital health intervention solutions or via a digital and/or physical asynchronous co-design process. Specifically, the concept of “low-contact co-design” involving the use of technology to replicate the use of physical workshops conducted at different times and locations has been proposed to navigate the process of co-design when physical proximity is a challenge, which provides co-designers with new opportunities and possibilities for engaging with communities [92].

An additional strength of this study was the use of co-design methodology, which generates unique, insightful contributions and progresses unknowns. Nevertheless, such methods do not have the significance of larger-scale population research that will be derived from future clinical trials aimed at examining the efficacy of the app for improving health outcomes. Co-designing with a predetermined output (eg, a smartphone app–based intervention) is challenging as it constrains the process. However, we were subject to limitations of the funding for the current project and funding models more broadly, which require precise descriptions about the format and delivery of interventions in funding applications. While this approach allowed the research team to focus on exploring digital solutions, it may have limited the ability to critically evaluate whether an app was the ideal solution for delivering this intervention. Notwithstanding, a key component of EFT training involves sending reminders or prompts to participants to practice the mental imagery exercises, which is easily facilitated via techniques such as automated smartphone notifications. Since smartphone app development remains expensive, there is an ongoing tension between deep engagement with members of the community and conducting consumer market research or beta testing, the latter of which may be more economically efficient. Notwithstanding, consumer market research would not allow unique insights into EFT intervention development and the concept of future discounting more broadly.

Finally, the inclusion of both highly experienced facilitators with expertise in co-design [68] and content experts in nutrition and behavioral science within the project team is considered a strength and undoubtedly enhanced the research process. Indeed, the use of multi-disciplinary teams with different content expertise and methodological viewpoints is deemed critical for developing digital health solutions for complex problems [93].

### Implications for Future Research and Health Policy or Practice

This study provides a blueprint for other researchers to determine the feasibility of using co-design practices in developing digital health behavior change mobile apps as well as EFT interventions more broadly. Furthermore, it would be useful to determine the extent to which co-designed apps enhance effectiveness in a more formal evaluative capacity. Nevertheless, the growing use of such practices speaks to the potential for creating sustainable and personalized digital preventive health programs to target a reduction in chronic conditions, a key priority area for Australian and global health strategies [94]. Finally, the insights gained from this project will help inform future large-scale clinical trials in prerisk or at-risk groups aimed at testing the feasibility, acceptability, and efficacy of the intervention alongside ongoing usability testing. Such trials could also serve the dual purpose of highlighting the cost-effectiveness of digital health interventions via an economic evaluation [95], which is critical for reducing the gap between research and end user needs and increasing clinical research utility [96].

Importantly, this project represents the first phase of the co-design process. Specifically, co-design uses an iterative process whereby consumers and other key stakeholders are continuously involved in refinement and development of the intervention in response to ongoing feedback and changing needs. In addition, future co-design work could involve health professionals who may be involved in facilitating behavior change or self-management of chronic conditions in individuals. Indeed, the current finding that trust could be enhanced via recommendations from credible sources (eg, psychologists), which is essential for ongoing use, supports the idea of engaging health care professionals in the co-design process. Furthermore, previous research has identified that a lack of health professional recommendations is a major barrier to self-management app use primarily due to the preparedness and capacity of such professionals [97]. Thus, such engagement would likely facilitate a better understanding of health professionals’ use of digital health apps in practice to support wide-scale implementation and community evaluation in the real world.

Finally, participants identified a range of suggested improvements that could be integrated into future iterations of the app. Some of these improvements involved using additional BCTs, namely, the active ingredients of interventions that are critical for changing behavior or the key mechanisms of action [21]. These included self-monitoring of behavior (ie, tracking against health goals) and providing information about health consequences if health goals were not achieved. Future research should aim to map these suggested improvements onto the taxonomy of BCTs based on relevant health behavior change theories and a theoretical framework known as the Theoretical Domains Framework [98,99], which was developed by behavioral scientists and implementation researchers. Such mapping could help inform which combination of BCTs to include in future versions of the app based on an evaluation of potential effectiveness drawn from the existing literature alongside the existing BCTs used. Specifically, EFT uses several techniques described in the behavior change taxonomy such as goal- and planning-related strategies as well as comparing future goals with existing behaviors [21].

### Conclusions

This study used a co-design process to inform the development of the “Luminaut” app, a digital EFT intervention that could potentially improve and maintain health-promoting behaviors and disrupt the psychological phenomenon known as future discounting (ie, valuing smaller, immediate rewards over larger, delayed rewards). Such interventions may lead to an enhanced adoption of healthier lifestyle changes amongst Australian adults who are currently in the prerisk or at-risk phase for chronic conditions. Using co-design methods to refine the design of digital health interventions may improve the relevance, scalability, sustainability, and engagement for end users. Engagement with digital interventions is a priority to ensure long-lasting behavior change and enable healthy ageing of the Australian population. The current project was valuable in gaining consumer perspectives about what makes the “Luminaut” app a relevant and engaging tool to promote adherence to health-related behavior change interventions and, in turn, has the potential to reduce the risk and burden of chronic conditions.

## Supplementary material

10.2196/74099 Multimedia Appendix 1 Description of the app naming competition.

10.2196/74099 Multimedia Appendix 2 Description of the workshops.

10.2196/74099 Multimedia Appendix 3 Project data report.

10.2196/74099 Multimedia Appendix 4 Characteristics of the enrolled participants according to workshop attendance.

10.2196/74099 Checklist 1 GRIPP2-SF checklist.

## Abbreviations

BCT: behavior change technique
EFT: Episodic Future Thinking
GRIPP2-SF: Guidance for Reporting Involvement of Patients and Public Short Form
IRSAD: Index of Relative Socio-economic Advantage and Disadvantage
LGA: Local Government Areas
mHealth: mobile health
NCD: noncommunicable disease
REDCap: Research Electronic Data Capture

## References

[1] (2024). Noncommunicable diseases.

[2] (2018). Noncommunicable diseases country profiles 2018.

[3] Budreviciute A, Damiati S, Sabir DK (2020). Management and prevention strategies for non-communicable diseases (NCDs) and their risk factors. Front Public Health.

[4] Anderson E, Durstine JL (2019). Physical activity, exercise, and chronic diseases: a brief review. Sports Med Health Sci.

[5] Afshin A, Sur PJ, Fay KA (2019). Health effects of dietary risks in 195 countries, 1990–2017: a systematic analysis for the Global Burden of Disease Study 2017. Lancet.

[6] Hu FB (2024). Diet strategies for promoting healthy aging and longevity: an epidemiological perspective. J Intern Med.

[7] Middleton KR, Anton SD, Perri MG (2013). Long-term adherence to health behavior change. Am J Lifestyle Med.

[8] (2023). National health survey. Australian Bureau of Statistics.

[9] Madden GJ, Bickel WK (2010). Impulsivity: The Behavioral and Neurological Science of Discounting.

[10] Lin H, Epstein LH (2014). Living in the moment: effects of time perspective and emotional valence of episodic thinking on delay discounting. Behav Neurosci.

[11] Hershfield HE (2011). Future self-continuity: how conceptions of the future self transform intertemporal choice. Ann N Y Acad Sci.

[12] Kakoschke N, Cox DN, Ryan J (2023). Disrupting future discounting: a commentary on an underutilised psychological approach for improving adherence to diet and physical activity interventions. Public Health Nutr.

[13] Bickel WK, Jarmolowicz DP, Mueller ET, Koffarnus MN, Gatchalian KM (2012). Excessive discounting of delayed reinforcers as a trans-disease process contributing to addiction and other disease-related vulnerabilities: emerging evidence. Pharmacol Ther.

[14] Amlung M, Petker T, Jackson J, Balodis I, MacKillop J (2016). Steep discounting of delayed monetary and food rewards in obesity: a meta-analysis. Psychol Med.

[15] Kakoschke N, Hawker C, Castine B, de Courten B, Verdejo-Garcia A (2018). Smartphone-based cognitive bias modification training improves healthy food choice in obesity: a pilot study. Eur Eat Disord Rev.

[16] Atance CM, O’Neill DK (2001). Episodic future thinking. Trends Cogn Sci (Regul Ed).

[17] Peters J, Büchel C (2010). Episodic future thinking reduces reward delay discounting through an enhancement of prefrontal-mediotemporal interactions. Neuron.

[18] Szpunar KK, Spreng RN, Schacter DL (2014). A taxonomy of prospection: introducing an organizational framework for future-oriented cognition. Proc Natl Acad Sci U S A.

[19] Boyer P (2008). Evolutionary economics of mental time travel?. Trends Cogn Sci (Regul Ed).

[20] Sofis MJ, Lemley SM, Jacobson NC, Budney AJ (2022). Initial evaluation of domain-specific episodic future thinking on delay discounting and cannabis use. Exp Clin Psychopharmacol.

[21] Michie S, Richardson M, Johnston M (2013). The behavior change technique taxonomy (v1) of 93 hierarchically clustered techniques: building an international consensus for the reporting of behavior change interventions. Ann Behav Med.

[22] Marques MM, Wright AJ, Corker E (2023). The Behaviour Change Technique Ontology: Transforming the Behaviour Change Technique Taxonomy v1. Wellcome Open Res.

[23] Michie S, Thomas J, Johnston M (2017). The Human Behaviour-Change Project: harnessing the power of artificial intelligence and machine learning for evidence synthesis and interpretation. Implement Sci.

[24] Nielsen L, Riddle M, King JW (2018). The NIH Science of Behavior Change Program: transforming the science through a focus on mechanisms of change. Behav Res Ther.

[25] Schacter DL, Benoit RG, Szpunar KK (2017). Episodic future thinking: mechanisms and functions. Curr Opin Behav Sci.

[26] Ward AM (2016). A critical evaluation of the validity of episodic future thinking: a clinical neuropsychology perspective. Neuropsychology.

[27] Schenk PM, Wright AJ, West R (2023). An ontology of mechanisms of action in behaviour change interventions. Wellcome Open Res.

[28] Tulving E (1993). What Is episodic memory?. Curr Dir Psychol Sci.

[29] Szpunar KK (2010). Episodic future thought: an emerging concept. Perspect Psychol Sci.

[30] Suddendorf T, Corballis MC (1997). Mental time travel and the evolution of the human mind. Genet Soc Gen Psychol Monogr.

[31] Brown JM, Stein JS (2022). Putting prospection into practice: methodological considerations in the use of episodic future thinking to reduce delay discounting and maladaptive health behaviors. Front Public Health.

[32] Ye J yan, Ding Q yu, Cui J fang (2022). A meta-analysis of the effects of episodic future thinking on delay discounting. Q J Exp Psychol (Hove).

[33] Rösch SA, Stramaccia DF, Benoit RG (2022). Promoting farsighted decisions via episodic future thinking: a meta-analysis. J Exp Psychol Gen.

[34] Colton E, Connors M, Mahlberg J, Verdejo-Garcia A (2024). Episodic future thinking improves intertemporal choice and food choice in individuals with higher weight: a systematic review and meta-analysis. Obes Rev.

[35] Benoit RG, Gilbert SJ, Burgess PW (2011). A neural mechanism mediating the impact of episodic prospection on farsighted decisions. J Neurosci.

[36] Peters J, Büchel C (2011). The neural mechanisms of inter-temporal decision-making: understanding variability. Trends Cogn Sci (Regul Ed).

[37] Rung JM, Madden GJ (2019). Demand characteristics in episodic future thinking II: the role of cues and cue content in changing delay discounting. Exp Clin Psychopharmacol.

[38] Stein JS, Wilson AG, Koffarnus MN, Daniel TO, Epstein LH, Bickel WK (2016). Unstuck in time: episodic future thinking reduces delay discounting and cigarette smoking. Psychopharmacology (Berl).

[39] Daniel TO, Stanton CM, Epstein LH (2013). The future is now: reducing impulsivity and energy intake using episodic future thinking. Psychol Sci.

[40] Daniel TO, Said M, Stanton CM, Epstein LH (2015). Episodic future thinking reduces delay discounting and energy intake in children. Eat Behav.

[41] Snider SE, LaConte SM, Bickel WK (2016). Episodic future thinking: expansion of the temporal window in individuals with alcohol dependence. Alcohol Clin Exp Res.

[42] Sze YY, Daniel TO, Kilanowski CK, Collins RL, Epstein LH (2015). Web-based and mobile delivery of an episodic future thinking intervention for overweight and obese families: a feasibility study. JMIR Mhealth Uhealth.

[43] O’Neill J, Daniel TO, Epstein LH (2016). Episodic future thinking reduces eating in a food court. Eat Behav.

[44] Leahey TM, Gorin AA, Wyckoff E (2020). Episodic future thinking, delay discounting, and exercise during weight loss maintenance: the PACE trial. Health Psychol.

[45] Mertens ECA, Siezenga AM, Tettero T, van Gelder JL (2022). A future orientation intervention delivered through a smartphone application and virtual reality: study protocol for a randomized controlled trial. BMC Psychol.

[46] Mertens ECA, Siezenga AM, van der Schalk J, van Gelder JL (2024). A novel smartphone-based intervention aimed at increasing future orientation via the future self: a pilot randomized controlled trial of a prototype application. Prev Sci.

[47] Persson DR (2023). Episodic future thinking as digital micro-interventions.

[48] Persson DR, Bardram JE, Bækgaard P (2024). Perceptions and effectiveness of episodic future thinking as digital micro-interventions based on mobile health technology. Digit Health.

[49] Mrklas KJ, Barber T, Campbell-Scherer D (2020). Co-design in the development of a mobile health app for the management of knee osteoarthritis by patients and physicians: qualitative study. JMIR Mhealth Uhealth.

[50] Boyd H, McKernon S, Mullin B, Old A (2012). Improving healthcare through the use of co-design. N Z Med J.

[51] Davis A, Tuckey M, Gwilt I, Wallace N (2023). Understanding co‐design practice as a process of “welldoing”. Int J Art Design Ed.

[52] Giacomin J (2014). What is human centred design?. Design J.

[53] Glasziou P, Chalmers I (2016). Paul glasziou and iain chalmers: is 85% of health research really “wasted”?. BMJ.

[54] Chalmers I, Glasziou P (2009). Avoidable waste in the production and reporting of research evidence. Lancet.

[55] Scariot CA, Heemann A, Padovani S (2012). Understanding the collaborative-participatory design. Work.

[56] Frank P, Young WR (2025). Remote Monitoring and Wearable Devices in Healthcare.

[57] Burke LE, Ma J, Azar KMJ (2015). Current science on consumer use of mobile health for cardiovascular disease prevention: a scientific statement from the American Heart Association. Circulation.

[58] Malloy JA, Partridge SR, Kemper JA, Braakhuis A, Roy R (2022). Co-design of digital health interventions for young adults: protocol for a scoping review. JMIR Res Protoc.

[59] Winters N, Oliver M, Langer L, Unterhalter E (2020). Measuring the Unmeasurable in Education.

[60] Ferguson C, Hickman LD, Turkmani S, Breen P, Gargiulo G, Inglis SC (2021). “Wearables only work on patients that wear them”: barriers and facilitators to the adoption of wearable cardiac monitoring technologies. Cardiovasc Digit Health J.

[61] Tzimourta KD (2025). Human-centered design and development in digital health: approaches, challenges, and emerging trends. Cureus.

[62] Carrera Diaz K, Yau J, Iverson E (2025). Human-centered design approach to building a transition readiness mHealth intervention for early adolescents. J Pediatr Psychol.

[63] An Q, Kelley MM, Hanners A, Yen PY (2023). Sustainable development for mobile health apps using the human-centered design process. JMIR Form Res.

[64] Hanrahan M, Wilson C, Keogh A (2025). How can patients shape digital medicine? A rapid review of patient and public involvement and engagement in the development of digital health technologies for neurological conditions. Expert Rev Pharmacoecon Outcomes Res.

[65] Kilfoy A, Hsu TCC, Stockton-Powdrell C, Whelan P, Chu CH, Jibb L (2024). An umbrella review on how digital health intervention co-design is conducted and described. NPJ Digit Med.

[66] Staniszewska S, Brett J, Simera I GRIPP2 reporting checklists: tools to improve reporting of patient and public involvement in research. BMJ.

[67] (2015). Innovation by design: how design enables science and technology research to achieve greater impact.

[68] Sanders EBN, Stappers PJ (2008). Co-creation and the new landscapes of design. CoDesign.

[69] Gero J, Milovanovic J (2021). The situated function-behavior-structure co-design model. CoDesign.

[70] Rashmi R, Mohanty SK (2023). Examining chronic disease onset across varying age groups of Indian adults using competing risk analysis. Sci Rep.

[71] Carroll JK, Moorhead A, Bond R, LeBlanc WG, Petrella RJ, Fiscella K (2017). Who uses mobile phone health apps and does use matter? A secondary data analytics approach. J Med Internet Res.

[72] Leask CF, Sandlund M, Skelton DA (2019). Framework, principles and recommendations for utilising participatory methodologies in the co-creation and evaluation of public health interventions. Res Involv Engagem.

[73] Guest G, Bunce A, Johnson L (2006). How many interviews are enough? An experiment with data saturation and variability. Field methods.

[74] Sanders E, Stappers PJ (2012). Convivial Toolbox: Generative Research for the Front End of Design.

[75] Pettit CJ, Glackin S, Trubka R (2014). A co-design prototyping approach for building a Precinct Planning Tool. ISPRS Ann Photogramm Remote Sens Spatial Inf Sci.

[76] (2021). Socio-economic indexes for areas (SEIFA). Australian Bureau of Statistics.

[77] Braun V, Clarke V, Cooper H, Camic PM, Long DL, Panter AT, Rindskopf D, Sher KJ, Thematic analysis (2012). Research Designs: Quantitative, Qualitative, Neuropsychological, and Biological Ed.

[78] Braun V, Clarke V (2021). One size fits all? What counts as quality practice in (reflexive) thematic analysis?. Qual Res Psychol.

[79] Pienkowska A, Ang CS, Mammadova M, Mahadzir MDA, Car J (2023). A diabetes education app for people living with type 2 diabetes: co-design study. JMIR Form Res.

[80] Tay BSJ, Edney SM, Brinkworth GD (2021). Co-design of a digital dietary intervention for adults at risk of type 2 diabetes. BMC Public Health.

[81] Asbjørnsen RA, Hjelmesæth J, Smedsrød ML (2022). Combining persuasive system design principles and behavior change techniques in digital interventions supporting long-term weight loss maintenance: design and development of eCHANGE. JMIR Hum Factors.

[82] Gibson I, Neubeck L, Corcoran M (2024). Development of a digital health intervention for the secondary prevention of cardiovascular disease (INTERCEPT): co-design and usability testing study. JMIR Hum Factors.

[83] Asbjørnsen RA, Wentzel J, Smedsrød ML (2020). Identifying persuasive design principles and behavior change techniques supporting end user values and needs in eHealth interventions for long-term weight loss maintenance: qualitative study. J Med Internet Res.

[84] Alahäivälä T, Oinas-Kukkonen H (2016). Understanding persuasion contexts in health gamification: a systematic analysis of gamified health behavior change support systems literature. Int J Med Inform.

[85] Sardi L, Idri A, Fernández-Alemán JL (2017). A systematic review of gamification in e-Health. J Biomed Inform.

[86] Sauro J, Lewis JR (2011). When designing usability questionnaires, does it hurt to be positive?.

[87] Agapie E, Haldar S, Poblete SG (2022). Using HCI in cross-disciplinary teams: a case study of academic collaboration in HCI-Health teams in the US using a team science perspective. Proc ACM Hum-Comput Interact.

[88] Waddell A, Seguin JP, Wu L (2024). Leveraging implementation science in human-centred design for digital health.

[89] Agius SJ (2013). Qualitative research: its value and applicability. Psychiatrist.

[90] Kontos E, Blake KD, Chou WYS, Prestin A (2014). Predictors of eHealth usage: insights on the digital divide from the Health Information National Trends Survey 2012. J Med Internet Res.

[91] Arias López M del P, Ong BA, Borrat Frigola X (2023). Digital literacy as a new determinant of health: a scoping review. PLOS Digit Health.

[92] Davis A, Gwilt I, Wallace N, Langley J (2021). Low-contact co-design: considering more flexible spatiotemporal models for the co-design workshop. Strategic Design Res J.

[93] Marwaha JS, Landman AB, Brat GA, Dunn T, Gordon WJ (2022). Deploying digital health tools within large, complex health systems: key considerations for adoption and implementation. NPJ Digit Med.

[94] Murray E, Hekler EB, Andersson G (2016). Evaluating digital health interventions: key questions and approaches. Am J Prev Med.

[95] Slattery P, Saeri AK, Bragge P (2020). Research co-design in health: a rapid overview of reviews. Health Res Policy Sys.

[96] Ioannidis JPA (2016). Why most clinical research is not useful. PLoS Med.

[97] Bradway M, Morris RL, Giordanengo A, Årsand E (2020). How mHealth can facilitate collaboration in diabetes care: qualitative analysis of co-design workshops. BMC Health Serv Res.

[98] Cane J, O’Connor D, Michie S (2012). Validation of the theoretical domains framework for use in behaviour change and implementation research. Implementation Sci.

[99] Atkins L, Francis J, Islam R (2017). A guide to using the Theoretical Domains Framework of behaviour change to investigate implementation problems. Implementation Sci.

