# Supplementary material for: Digital Episodic Future Thinking Intervention (Luminaut): Co-Design and Iterative Development Study
Source: JMIR Hum Factors. 2026 May 6;13:e74099. doi: 10.2196/74099 (PMC13148339; doi:10.2196/74099)
Supplement: Multimedia Appendix 1 [file humanfactors-v13-e74099-s001.docx]

**Multimedia Appendix 1**

Description of app naming competition

The researchers (NK, CH, MR) held an app naming competition between the 15^th^ and 19^th^ of July 2024 on Viva Engage, namely, an online social media platform available to all CSIRO staff members, students and affiliates. A brief description of the primary purpose of the app was included, and staff were invited to share their most creative name for the app. Individuals were given specific criteria to adhere to when devising a name for the app, which are as follows: i) the name needed to be short (i.e., either a real or made-up phrase or acronym), non-descriptive (i.e., unrelated to the function of the app), and it could not already be in use. By entering the competition, participants were given the chance to win an AUD $60 gift card. Once the competition closed, all the potential app names were collated into an excel file and the researchers (NK, CH, MR) reviewed each of potential names independently and created a short-list of names they each preferred. A meeting with a colleague from the Business and Development (B&D) team was held on 23^rd^ July 2024 to determine the level of agreement between the researchers, wherein a combined short-list of preferred names for the app was created. From here, the researchers and B&D colleague performed searches (i.e., using the TM checker, which is free and available on the IP Australia website: <https://tmchecker.ipaustralia.gov.au/> and internet browsers) to determine the viability of trade marking each name that was chosen. The name ‘*Luminaut’* was agreed upon by the researchers and was ultimately selected because i) it met the aforementioned criteria (i.e., short, non-descriptive and not already in use), and ii) it has an underlying meaning – that is, “a word combining ‘lumina’ (light or clarity) with ‘naut’ (navigator) to represent navigating a bright future”.
