# Supplementary material for: Digital Episodic Future Thinking Intervention (Luminaut): Co-Design and Iterative Development Study
Source: JMIR Hum Factors. 2026 May 6;13:e74099. doi: 10.2196/74099 (PMC13148339; doi:10.2196/74099)
Supplement: Multimedia Appendix 2 [file humanfactors-v13-e74099-s002.docx]

**Multimedia Appendix 3.** Detailed description of workshops.

*Workshop 1: ‘Discover’ and ‘Define’*

Workshop 1 commenced with a short presentation that provided an overview of the concept of future discounting. Following this, participants engaged in an ice-breaker activity that aided in building rapport between the participants, co-design facilitators, and researchers. A collaborative card-sorting activity was used to stimulate discussion and thinking about the types of activities and experiences that might relate to future discounting. The workshop then shifted to a story-based template to explore situations where future discounting might occur in everyday life, acknowledging the vast range of biopsychosocial factors that influence this behaviour, while also seeking insights into when an app-based intervention may be best received. After a short break, participants were asked to consider the vast range of applications on their personal mobile phones, and to explore those that they would broadly categorise as being ‘wellbeing apps’, ‘food apps’, ‘physical activity apps’ or ‘goal setting apps’. Following this, a stack of envelopes with reflection questions for small group discussions were placed onto the tables. Facilitators and researchers ceded the floor to participants during this activity, asking them to read these questions aloud and then steer these conversations.

*Workshop 2: ‘Develop’ and ‘Deliver’*

During workshop 2, participants were given a series of 81 icons that had been developed based on the results of the card sorting activity in workshop 1. This included 27 icons that represented activities that were focused on an immediate reward, 33 focused on a future reward, and 21 that were ambiguous or likely to be seen as leading to both an immediate and a future reward. Participants used these icons as prompts for constructing their storyboards, then were asked to re-sort these to confirm their allocation into these categories. In three of four workshops, an optional discussion was undertaken that explored external and systems-based barriers to engaging with the future, before a short break. The remainder of workshop 2 was then devoted to reviewing a series of 10 metaphors that might be used to structure the navigation of the app through a group discussion and collaboratively reviewing the recommendations to be included in the final report to be sent to Appliquette.
