# Supplementary material for: Digital Episodic Future Thinking Intervention (Luminaut): Co-Design and Iterative Development Study
Source: JMIR Hum Factors. 2026 May 6;13:e74099. doi: 10.2196/74099 (PMC13148339; doi:10.2196/74099)
Supplement: Multimedia Appendix 3 [file humanfactors-v13-e74099-s003.docx]

**Multimedia Appendix 4.** Project data report.


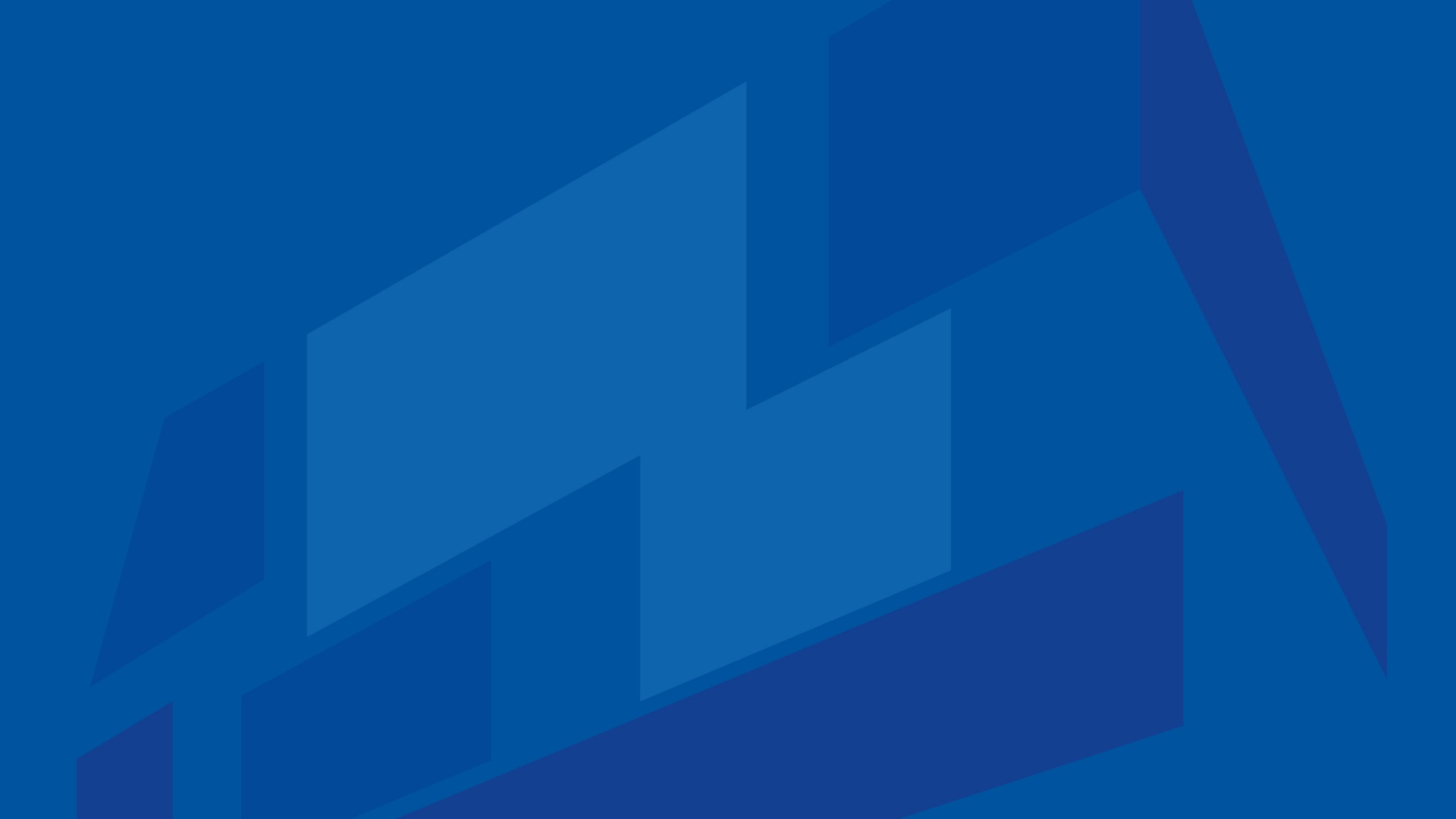

**Co-designing a smartphone application to promote future-oriented thinking**

Project Data Report

July 2024

Co-designing a smartphone application to promote future-oriented thinking

Project data report

July 2024

Prof Ian Gwilt*, Dr Aaron Davis, Dr Kate Little

UniSA Match Studio

*Ian.Gwilt@unisa.edu.au

**Background**

This report presents the outcomes from a series of co-design workshops facilitated by Prof Ian Gwilt, Dr Aaron Davis, and Dr Kate Little. The workshops explored the concept of Future Discounting, and in particular, the design of an app-based intervention to assist people to engage in Episodic Future Thinking (EFT).

### The challenge

‘Non-communicable diseases (NCD), including cardiovascular diseases, some cancers, type 2 diabetes (T2DM), and respiratory diseases, are largely driven by modifiable, lifestyle risk factors (i.e., physical inactivity and poor diet) (1, 2) and are the leading cause of illness, disability, and mortality in Australia (3). In 2020-21, almost half of the Australian population, equivalent to 11.6 million people, were living with at least one chronic condition (4). Although consuming a nutritious diet and engaging in regular physical activity are critical to reducing the risk of chronic diseases, many people find it challenging to adopt and maintain health-promoting behaviours (5). Low adherence to lifestyle behaviour change interventions remains an ongoing challenge potentially due to the human psychological tendency to prefer immediate rewards (e.g., sedentary activities and consuming discretionary foods) over long-term future rewards (e.g., heart health); a phenomenon referred to as future discounting (6).

### The intervention

EFT, a form of prospective thought, involves mentally envisioning oneself experiencing specific, personal, and detailed future-oriented events (7). EFT is one type of psychological intervention that provides promise in disrupting one’s tendency to discount the future by helping individuals consider the potential future consequences of behaviours that impact healthy ageing and the development of chronic diseases. Previous studies have used smartphone technology to deliver EFT interventions in real-world settings (8, 9); however, no research to date has used co-design methods – that is, actively collaborating with people who directly benefit from the co-design process (10, 11) – to create an engaging EFT intervention with end-users who are in the pre-risk (i.e., 25-44 years of age) phase for chronic disease to facilitate long-lasting, health promoting behaviour change.

The ‘Future Self 4 Health’ app has the potential to disrupt future discounting (i.e., valuing smaller, immediate rewards over larger, delayed rewards) and lead to greater adoption of healthier lifestyle changes among Australian adults who are currently in the pre-risk or at-risk phase for chronic disease. Using co-design methods to refine the design of lifestyle interventions to make them relevant, scalable, sustainable, and engaging for end-users is a priority area to elicit long-lasting behaviour change to reduce the risk and burden of chronic disease and enable the Australian population to age healthily.

**Table of Contents**

1. Executive summary and high-level insights from the co-design process 5
2. Workshop structures and activities 6
3. Data from Workshop 1 series 7
4. Interim recommendations following Workshop 1 series 17
5. Data from asynchronous feedback process 19
6. Data from Workshop 2 series 22

Appendix I: Workshop templates and resources 35

Appendix II: Explaining Future Discounting to a Friend 41

## 1. Executive summary and high-level insights

The project delivered a series of findings that relate to the app itself, as well as to higher-level strategies to engage with the concept of future discounting. Key insights relating to Future Discounting and insights specific to the App concept are summarised in the tables below.

In reading these insights it should be noted that in general, while the workshops provided a variety of instrumental insights into how goal setting processes might be linked to the app, there was an overarching challenge in participants engaging with the higher-level psychological concept of Episodic Future Thinking.

### Future Discounting Related Insights

| It is important to emphasise the power of visualising oneself in the medium-term future, and to clarify the difference from mainstream goal setting and implementation |
| --- |
| A practical description of activities that illustrate the concept (e.g. compose a letter to future me in a week, delivered in one week) may be the most effective way of communicating this concept rather than a more mechanistic explanation |
| The concept is complicated by socially-endorsed and immediately-enjoyable future beneficial behaviours (e.g. I like eating salad). This may be addressed by an exploration of the terminology of ‘discounting’ versus ‘investing’ and the shift away from the notion of a binary between choices for now or the future. |
| Assistance with initial engagement (largely framed in the workshops as goal setting, but relating to any kind of future visioning) at first (prefilled or examples, live composition of cue, perhaps use icons/images) |
| While treated in the literature largely as an individual psychological phenomenon, a social element, such as a potential role for a mentor may be an interesting opportunity |
| The complexity of tasks associated with a future discounting intervention, as well as an associated underlying perception of the possibility of failure were identified as potential barriers |
| The language of ‘health goals’ led participants to assume the intervention was about personal goal setting, rather than catalysing thinking about the future. Developing alternative language may assist in communicating the concept and science of Future Discounting. |

### App Related Insights

| Introduction to the app concept should include:   1. Start with thinking about choices and positive future benefits (see the Explain Future Discounting to a Friend activity – ‘Future you will thank you’) 2. Add the impact of simply visualising yourself in the future – supported by scientific underpinnings 3. Finally, include an introduction of the EMA and its purpose (if this is part of the intervention) |
| --- |
| There is potential to explore the integration of generative AI in helping create cues or create visualisations of cues |
| It is important to introduce the Ecological Momentary Assessment and how it contributes to the overall intervention (if at all) |
| A dashboard could effectively support the concept and facilitate navigation using one of the identified visual metaphors of tree, stepping stones, or road (see visual metaphors section) |
| Customisation has been identified as important, such as customising push notification frequency, time of day, etc |
| General best practice design useability principles should be applied, such as slider directions to being consistent to reduce confusion, or the introduction of graphic feedback (e.g. an emoji changing from tired to refreshed in line with the selection on a slider to reinforce the selection that is being made) |
| The app requires a considerable amount of personal investment (time and effort), and an aversion to constraint was considered a potential barrier to use. Therefore, consider:   1. Providing an acknowledgement of this upfront 2. Introducing efforts to reduce friction (customisation of notifications, help with cues and navigation, etc) 3. Differentiating between this app and a goal setting app, and 4. Making the experience more (immediately) rewarding (aesthetic rewards, badges, etc for small steps) |
| Introduce gamification. Opportunities included rewards/badges/changing avatar or tree or movement along the path/streaks and optional social sharing |
| Potential behaviours to reward: your own accomplishments, completing EMA/viewing cues, reflecting on future thinking process, setting goals, social (e.g. high fiving a friend) |
| Potential rewards: growth of tree or flower, badges, a gallery, changing avatar, a simple list of achievements, streaks. |
| Positive language is important. Some participants discovered that the positive language in the app was a reward in its own right. |

## 2. Workshop structures and activities

A series of 8 workshops were conducted between November 2023 and March 2024. Participants were offered a series of times and locations, including both a city-based location and a suburban location, and day or evening time slots. Four daytime and two evening workshops were facilitated at the University of South Australia City West Campus and two daytime workshops at the Christie’s Downs Community House. A total of n=30 participants were engaged overall, and each workshop ran for 2.5 hours.

The structures below provide a guide to the flow of activities in these workshops. Templates and resources have been included in appendix I.

### Workshop 1 structure

|  | **Arrival** |
| --- | --- |
| 5 | **Opening remarks** – Acknowledgement of Country, purpose and objectives of the workshop |
| 15 | **Future discounting introduction** |
| 30 | **Activity 1:** Card sort: What is and what isn’t future discounting |
| 25 | **Activity 2:** Where and when activity stories |
| 10 | **Break** |
| 30 | **Activity 3:** App inventory exercise |
| 30 | **Activity 4:** Specific app test feedback |
| 5 | **Wrap up** |
|  | **Close** |

### Workshop 2 structure

|  | **Arrival** |
| --- | --- |
| 5 | **Opening remarks** – Acknowledgement of Country, purpose and objectives of the workshop |
| 5 | **Future discounting introduction** recap from previous workshop |
| 20 | **Activity 1:** Explaining future discounting to a friend using storyboard template and noun project icons |
| 10 | **Activity 2:** Development of sorting game activity based on selected icons |
| (10) | **Optional Activity:** Financial and other barriers to achieving goals  Mapping example icons and types of visualisations to financial, work, family, stress, etc. barriers |
| 10 | **Break** |
| 15 | **Activity 4:** Metaphors and dashboards review  Avatars, tree growing, looking through a portal, adjusting a date, advent calendar, road, stepping stones / islands, seasons, graphs / abstract data visualisation, list |
| 40 | **Activity 5:** Specific feedback on current app state   - What milestones give a badge? - How much difference does a photo make? What if it is a stock image? (Use cards from first workshop + icon cards) |
| 5 | **Wrap up** |
|  | **Close** |

3: Data from Workshop 1 **Series**

Data presented in the tables that follow includes both participant documentation, and facilitator observation. This documentation approach is in line with best practice in co-design research and allows greater depth and context to be provided with data than participant contributions alone.

Where appropriate, themes have been grouped, and **bold** **text** indicates a key insight that has been translated into a recommendation or finding.

| **Themes for using health, wellbeing, goal setting, food, and fitness apps (WS1.1 only)** | | | |
| --- | --- | --- | --- |
| bonuses/ savings | cost | expert recommendation | other |
| **brand trust** | **customisable** | Function | routine |
| connects with something else | ease of use | **gamification/ streaks/** motivation | social |
| convenience | enjoyable | interest | **stats/ data display** |

| **Reasons for using health, wellbeing, goal setting, food, and fitness apps (WS1.1 only)** | | | |
| --- | --- | --- | --- |
| Rewards | Savings | Convenience | Discounts |
| Free | Super convenient | Really useful | easy |
| I like to enter competitions | large range | Accurate recording | see info at a glance |
| Guided mindfulness | Linked to my watch | Changes with me | **track progress** |
| **Recommended by psychologist** | avoids need to talk to anyone to order (uber eats) | I can book my gym class | I go to the club (gym) |
| **Gamified' to a degree, progress tracking, move through course** | My fitness pal free, allows calorie tracking, weight tracking | can track my steps and other parameters over time | Social link, follow friends, can see their activity = motivation |
| **Includes 'streaks' which are motivating** | Untapped - social, record keeping, badges | I get points then $ credit | Linked to my watch |
| Quick options that I can pick and choose | Emails from my fitness pal include good recipes | Gives me direct feedback | **Feedback that I've hit my goals** |
| Connects to my Garmin watch | Monitor personal goals | **Familiar brand** | Health: curiosity |
| connected to my scales | Works in the background | Easy to use | Part of my routine |
| **can see composition breakdown and progress** | I use vouchers (e.g. 2 for 1, 25% off) | Connects to my watch | Kinda works in the background |
| Maintain streaks | Easy | Habit | Ring fit: Fun! |
| Like to record my bike rides/large walks - can check if I'm improving (Strava) | Fasting tracker - switched to count calories instead of intermittent fasting specifically | **Did like goal setting aspects of conquer challenges and small rewards in app for progressing** | choice in narrator, flexibility within app to suit preferences |
| Covers many different topics and styles | Paid for it so didn't want to waste my money | To get Qantas points for meeting my goal # of steps | Good stats about my health and performance |
| Get Qantas points for activity (e.g. walking certain # of steps) | friends can see inactivity = motivates (social pressure) | Like to see my heart rate and sleep activity tracked | My fitness pal can track fasting progress as well (multipurpose) |
| Pre-ordering | Just there on my phone | Put in claims (family health) | avoids need to leave the house (uber eats) |

| **Themes for NOT using health, wellbeing, goal setting, food, and fitness apps (WS1.1 only)** | | | |
| --- | --- | --- | --- |
| appearance/ui | didn't know I had | other | **cost** |
| brand trust | function | routine | interest |
| connects with something else | gamification/ streaks/ **motivation** | stats/data display | **time/effort** |

| **Reasons for NOT using health, wellbeing, goal setting, food, and fitness apps (WS1.1 only)** | | | |
| --- | --- | --- | --- |
| Not appealing | Not enough information | **Takes effort (to open, to do workouts)** | I can see info in other apps I am using |
| - no time to explore the apps | **Unrealistic targets** | Not part of my routine | Didn't even know I had it or could delete it (Fitness) |
| Have to think about it | I go to the stores infrequently and occasionally use them if the app offers a special price | Not prepared to make commitment to get results | Just haven't set it up |
| I prioritise having fun, ie games, youtube | No longer have fitbit | Not much benefit | Too demanding |
| abstract, ie 'mental health' | **Had to pay for challenges on conquer** | **Time consuming to enter everything** | Lost interest |
| Didn't even know I had it | Flo - can track mentsrual cycle in garmin(but not as detailed considering using flow again | Bad for my mental health | Not important to me |
| Not in my routine | Conquer challenges come with a medal I don't need - wasteful | Requires effort | Black and white - cals in vs cal out |
| I don't pay for extra features and now I have fitbit | keep trying to sell me upgrade | no longer wear fitbit | Don't like anything Apple |

| **Themes of moments for potential intervention** | | | |
| --- | --- | --- | --- |
| Starting something new | Idle time at home or at work (daytime) | **When using social media for too long** | **Time of day (various) depending on individual** |
| When feeling creative / looking for a new challenge / in the mindset for change | Stressed, tired, exhausted, overwhelmed, anxious, nervous, frustrated | **Planning day or activity** | **Day of week (various) Weekday / weekend (various)** |
| **Pay day, leadup to pay day, financial windfall (financial?)** | Hitting exercise / step / gym goals | Part of other changes – e.g. diet change |  |

| **Moments for potential intervention** | | | |
| --- | --- | --- | --- |
| Volunteering for extracurricular activities | Scrolling websites, sitting on the couch | ‘wasting’ time watching videos on social media | **Sleeping in, scrolling social media, watching tv** |
| Alert, feeling creative, wanting something to do | Feeling unenthusiastic about exercising | Shopping for the girls’ night / making a list | Use the holidays as a time to explore career options |
| Organised | Stressed or tired | **In the morning before breaky** | Completing my degree |
| At the supermarket buying food / staring into the pantry | Booking into my gym class | **Planning my work day** | Feeling overwhelmed at work |
| **Avoiding going to bed at night** | All through the day | **Tired after a long commute or a long day at work** | **Tired and have some spare time, evenings** |
| Stressed or frustrated at work or home | Grocery shopping | Every time I get in the car | Wandering away from my desk to make a cup of tea |
| Overwhelmed by life, parenting, work, family issues | Before events = less self-control | Day off with no excuse | **After work** |
| Use vending machine at work, which is generally bad food | Try to be more consistent – lose track of routine | **At the end of a long day at work** | On my phone during the day – constantly replying / reading |
| Have zero awareness of my spending habits | Thinking about eating a lolly or biscuit (or a third or fourth) | **Early in the morning / before sunrise** | A few weeks before we catch up |
| **Right before going to bed, on holidays** | Finished cooking, before I go to bed | **Before I go to bed, during the day, reminders** | End of the week, taking time |
| Deciding what to cook for dinner for family – stressed to find something that everyone would enjoy | **About to head out to an event where I don’t have control of the food choices and eating will be a part of the social activity** | Tired, want a moment to rest / reset, need to focus, hungry, socialising with friends | **Tired, right after I wake up in the morning and after dinner, on the couch / in bed** |
| Riding bike or active transport – not catching the bus | Handling Christmas food situations or study snacks | Working more and generating more income / savings | Spending more time in the gym and worked out more |
| In the kitchen | Spending less and saving more | Few long planning goals | Healthy dinner week |
| Stuck with too many choice projects. Some steps needed and regular work | **Closer to payday in order to prevent spending when resources are high** | **Comfy on the couch / tired after bike commuting / found on the couch** | Tired, not motivated to get dressed, focused on the distance to commute |
| Stressed / didn’t pack enough food / roughed for time | **Just after getting paid / using social media** | When I finish my current work project | Thinking about going for a walk, when I have free time |
| When I am not emotionally well: anxious, nervous, frustrated etc. | Before going to bed, when waking up, as I’m planning my day | Trying to spend more time out of the house with my children, deciding what to do | Overly sedentary, e.g. days off / weekends / struggling with physical aspects of work |
| Wanting to become more physically fit | **Watching TV on the couch on a weekend** | Feeling stressed or frustrated at the state of the house | **In the mindset for change – time and energy rich** |
| Return to following the Mediterranean diet | Use the school holidays as a time to factor in down time | Waking up one morning to do a yoga/ mindfulness session | Planning to go with Rach – on whatsapp. |
| Home late from work and feeling too tired (or lazy) to cook | Losing hours at night unproductively scrolling on my phone | Stressed about what to have for dinner because I’ve had a full on day | In the afternoon when I start thinking about cancelling exercise classes |
| Doing the housework | Staying in family unit | Idle time | Bored at work |
| At 10pm or just after getting into bed | Morning or just after coming back home from work | Before any social event |  |
| Couch at night | Can start now | Preparing meal plans |  |

| **Themes in triggers of considering the importance of combatting future discounting** | | | |
| --- | --- | --- | --- |
| Family member unwell physically or mentally | Financial stress | **Physical ability realisation / strength fail, out of breath etc.** | **When receiving good news about health – do more** |
| Personal health, bad news from GP, poor test results | Weigh-in | **Starting something new – new house, new job, etc.** | Injury |
| **Media story about a young person with illness** |  |  |  |

| **Triggers of considering the importance of combatting future discounting** | | | |
| --- | --- | --- | --- |
| Parents or extended family are unwell | **Unexpected expenses, inability to pay / have to live paycheck to paycheck** | Unable to do something due to a lack of strength / fracture / or other consequence of health condition | Family |
| Health | Bad news from the GP | Seeing someone completely overtaken with fear / anxiety | **Good news r.e. changes in body composition** |
| Jumping on the scales and finding I’m a few kilos heavier than expected | Running to the bus and being out of breath | Health issues – physical or mental | **Change in location / moving** |
| Health issues e.g. asthma, poor blood test results, anxiety | Family member diagnosed with illness e.g. diabetes | Major injury | Constantly feeling unwell |
| Thinking about your future self | **Showing compassion for my future self** | Cost of living | My work |
| Bank account | `Money to do things | Time is passing – rewarding time | Thinking about important goals |
| Sticky note on debit card | Experience by proxy of health scare / diagnosis | Being aware of negative consequences of short term choice | My GP giving me poor blood test results e.g. NAFLD or diabetes |
| Reprimanded at work for not getting things done on time | Someone selling one of my books for me | My dog wanting to spend more time outside | Personal connections to events / stories, my father smoking, friend ending up with cancer / voice box |
| Personally witnessing detrimental event, father sleep apnoea, thinking about what would happen if falling asleep driving | Personal experience | Friend, colleague, or family being diagnosed with illness, or dying | Media story about someone my age or younger having an illness or dying |
| Health scare | Partner being unsatisfied / unhappy | Mental health episode / break down | Changes to work situation, e.g. increased hours / workload |
| Future high score on diabetes test | Serios illness of partner or child | Increased blood-pressure | Mother having heart attack |
| Friendship | Availability of [ ] | High stress levels at work | Family health concerns being forefront |
| Large sudden expenses | Relationship strain from poor management of finances / organisation | Sudden change at work | Broke 10 years ago, not I save more |
| Several of my friends died due to bad health – I’m fitter now | Hiking / walks – do when you can | Family diagnosis with health condition | Adverse medical test results / screening results |
| Losing a family / friend | Looking at people who have a healthier life – mum, grandfather, family | Being aware of pros/cons of keeping to a gym routine when its not for muscle mass / building | Nail biting- knowing more about bacteria that lives under the nails |

| 1. **Without opening the app, what do you remember doing?** | | | |
| --- | --- | --- | --- |
| Goal setting 3, 6, 12 months | Food or physical activity | Visualisation of future activity / event statements | “I am … with … “ |
| Survey of how you felt | Is this important to you? Achievable? | How well can you visualise it? | Provided cues – remind about the future |
| Login – couldn’t get past | Repository, future self at 3, 6, 12 months | Goals you want to change | How you feel at the time |
| **Stuck in a loop** | Goal setting – feels good | Didn’t reward for achievement or recognise it | Pretty picture |
| Soft pastel colours | Welcoming and positive | Pick future events – challenging | **Same questions repeated** |
| Ebb and flow of day makes responding for right now challenging. Wanted to be positive | First time was honest, second time was too tired but didn’t want to bias so said I was good | **A vision board would help** | Hard to visualise the future |
| Time frames, 3, 6, 12 month | Hard to visualise | **Didn’t do anything!** | Choose food or activity and set some goals |
| **Confused about purpose** | **Lack of information** | Future situation caused confusion | **Lack of context** |
| Where are they? | **Looping – am I supposed to repeat this?** | Pretty / beautiful | Small goals – no wrong answers |
| **Could have used examples** | Felt like reding a prompt – transported to positive space | **Wording was challenging to get right in the interface** | **Cue was always 12 months – doesn’t work for me. I need 3 or 6 month first** |
| Cute animation | Goal – sparkly / poppy graphic | How tired etc. feelings | Liked “I will” instead of “I am” story oriented |
| Liked the language. Feel more immersed in what you want to do | Questions – 2 themes, PA and diet | Event? | Summary of last goal, 1 year ahead, prompt |
| Energy level, fatigue, alertness test | 3 parts | Detailed goals, visualised, summary of what I put in | How do you feel > I am feeling |
| 3, 6, 12 months | **Animated character– couldn’t bypass = annoyed** | Sub-questions – goals | Planning with people |
| Survey – prompt how you are feeling | **Unsure what it was for** | Initial questions, then trying to think about things you may not have thought, stepping through short, medium, long | **Asked same question over and over, couldn’t get past** |
| 3 months is quite long |  |  |  |

| 1. **Once you open the app, how easy is it to know what you are supposed to do?** | | | |
| --- | --- | --- | --- |
| **Would like to have my own avatar** | Not easy | Why is that there? | **Not clear why opens to dashboard** |
| **Once press the button it asks a question** | Only one option | Comes up to 12 month | Unclear how often to fill the survey in |
| 3 month goal? | **Would be easier if directing goals to be behaviours** | Should goals be related or not? | Emotion, connection. Long term or instant gratification |
| Connected | Future me’s problem = bad decision is negative. Good job past me would be useful. | **Every day v.s. every week – control over frequency** | **Goal setting. Small steps v.s. single goal?** |
| Not super easy | **Same thing over and over** | Feels like a mistake | **Goal setting was intuitive, the ongoing was confusing** |
| Getting stuck | **Survey appears again immediately** | Not intuitive | **Couldn’t see my own data / rhythm** |
| **Stuck in a loop** | **What does x have to do with y?** | Where are the rewards? – e.g. duolingo | Easy to follow prompts, unclear why |
| Big initial setup. Too hard and long. Took 15 mins | Have to have time to spend – warn me | Examples (of goals)? From community? | No scaffolding. |
| De-identified examples or framework would make it easier | Not very | Needs more information | Would like to be able to access dashboard at any point |
| Navigation | Scale | Confusing | **Link between goals and prompts not clear** |
| Quite self-explanatory – didn’t go through a lot | Connections not clear | **Easy to follow but hard to know ‘why?’** | **Data to demonstrate connection** |
| Pretty straight forward | Doesn’t tell you what’s coming | One screen instead of several for survey | Have to adjust sliding to move on |
| Complete on one [p] instead | **Why? Is missing** | No feedback on how you are going | Same with results on how progressing – where are they? |
| Goal / incentive is missing |  |  |  |

| 1. **When testing the app, which parts of the app did you use most?** | | | |
| --- | --- | --- | --- |
| They survey, because it kept coming up | **Kept popping up with the same thing** | Keeps presenting same survey | Survey kept looping |
| Complete survey button loop | Name of which survey might help | 1 part was repeated over and over | Could be – what do you need to go and do that (breaking down goals) |
| 3, 6, 12 are difficult. It would be good to see how they are connected | Prompts? | A ‘want help?’ or ‘guided tour’ would be helpful | Revisiting goal setting would be helpful |
| The survey – because I was forced to | Future events more beneficial than the health goals – adding where really painted the picture | Health goals – felt more locked in | Enjoyed the guidance – permission to do this |
| Linked 3, 6, 12 months rather than [mass] goal | Environment survey – it was all I could do | Goal setting – thinking in the moment | Enjoyed the story of goal setting |
| Continually being prompted for the scale | Goal setting | Goal setting | Assessment repeated 5-10 times |
| Repetitive – same prompt kept appearing | **Expecting feedback, e.g. “well done you’ve…”** | **Feedback would reinforce achievement** | Day slumped, how should I respond? |
| 1 walk through – only re-do survey as an option so disengaged | Simple = positive | **Straight into set a goal was jarring. Not done this before** | Missing how to get there with the goals |
| **Would like the satisfaction of “ticking things off”** | **Things to read / motivations / “teachable moments” are missing** | Simple little faults | Goal setting because it took longer |
| **Had to repeat because it didn’t save and I wanted to think about it** | Kept sending me to the same survey | Login (Couldn’t get past) | Double full stops were annoying |
| The survey repeats too much |  |  |  |

| 1. **What things to you feel are missing from the app? What could be added to make it easier to use?** | | | |
| --- | --- | --- | --- |
| Feedback – why – and response | Record link to outcome | **Graph to see progress** | **Score / medal** |
| Add a way to go back when you accidentally enter a quiz | **Link with other apps –if apps could talk to each other (e.g. steps each day) help ‘achieve’ goals** | Change the animation of the character | Time periods – not sure what these are for |
| **Icons at the bottom to jump between things** | Social – part of a group | Edit a goal to make grammatical sense or to change it | Demonstrate graph over time – inputs |
| Links – present data back | Links – goal linked to an activity | **Customisation with a picture – upload or photo library** | Information email – would be useful embedded in the app |
| Self report + comments. E.g. “I’m tired because” | Option to skip or return later | Infographic – short term / long term visually too similar | Customise colour and audio for visually impaired |
| Missing prompts / story / narrative | Toolbar | ‘Help’ feature | Feedback |
| Language too complex | Dashboard feel | Language was good | More visual |
| Baseline survey so prompts can be measured (reference point) | Smiling mind has gamification elements | Courses and learning? | Milestones / chunking problems down |
| Connections and logic are missing | Call to action missing | Cue – how I am – goal? | Reflecting editing and adjusting goals |
| A week later, a month later, how’s it going? | Visual prompt / mood board rather than just text? | Not too heavy visually. Welcoming and approachable | Disjointed at present |
| Accountability – why didn’t you do this? | Tweak or change goals? | Stretch goals? | Check in with the goals? |
| Introduction and context | Tutorial showing the cartoon – setting the goal etc. | Be able to skip through things | Article links (for why?) |
| Able to recap and links for more info | Option to change goals in a guided way | Encourage evolving the goals | Paid = quality = extra value |
| Prompts to guide you through | How often? | Is this all it is? | Context first to establish what it’s for |
| Links: Setting goals, visualising things, then self assessment appear disconnected | Guidance about how to set a goal and how to achieve it. “help me figure it out” | Free, no ads. If it’s expensive I won’t use it | More instructions and examples with option to not engage with them |
| **In other apps, repeated content makes me want to delete the app and disengage** | Can it be something different every time? (different categories of information or learning) | Create own avatar that you identify or connect with | Some exploration / context to help know what to do (e.g. infographic) |
| Introduction / context / about the app | “am I doing this right?” | Saving would be good. Lose faith when losing data | Fitness AND health goals |
| Icons that connect with goals | Less wordy |  |  |

| 1. **How do you think this app links to the concept of Future Discounting?** | | | |
| --- | --- | --- | --- |
| By putting in future scenarios – visualise but not too far away | Not clear how to connect the scenarios with the activities / behaivours | A person e.g. dietician would be more helpful | Support from a real person needed for accountability |
| Guidance for setting goals, e.g. suggestions, multiple choice to start thinking | Reminding of goals / long term | 12 months is not too far in the future and is achievable | Moment assessment = connect to now |
| E.g. Harribaut Advertisement | Terminology? Future discounting v.s. delayed reward v.s. delayed gratification v.s. future investing? (more positive?) | **Focused on visualising the future person** | “**opening the portal**” |
| **Making the connection between future and present self** | **Connecting all of the selves** | Goal setting part brings the future closer, the rest doesn’t seem related | Future but no discounting? |
| **Starting with 3 month goal not 12 month goal would be easier** | Incentives? | Financial pressures? | Want prompts and reminders for accountability |
| **Breaking goals down** | Level of commitment this week | Recognition | Reminder to rest / take it easy / enable a “cheat day”? |
| Checking in about how things are | Time scale? | Asking you think about ‘ideal future’ | Not an explicit link between now and the ideal future |
| The questions don’t help make the link | Check in after you’ve done it | Self awareness in the moment | Drilling down into the value of the goal for me could change over time. Being able to revisit is important |
| Goals at front of mind | **Struggle times = reminder of goal** | A cheerleader / personal prompt would be good | 3 months first, then 6 months, then 12 months would make more sense. Clear, less clear, hard to visualise |
| **Completely different goals or ‘chunked’ goals needs to be established. Not clear in introduction** | **Editable goals** | Can the grammar auto-correct? | Goal setting, short / medium / long term |
| You might not know | Reminders to stay focused on a goal | “future me” | “future benefit” |

| 1. **How do you think we could make it more obviously address Future Discounting** | | | |
| --- | --- | --- | --- |
| Teaching – theory is missing | Video introduction (not too long) | Infographic wasn’t memorable, but helped a bit | Links for later deep dive |
| Humour could be good | Hear a story of someone who has done it | Notification controls – customisable | Not enough there to use it consistently |
| A clear definition of what is meant by future discounting | **Summary background. This is what we are talking about.** | **First person / personalised examples** | **Left / right swipe for cards / images of ‘future discounting’ to practice recognising behaviours** |
| Understand why? | For long term use it needs to be fun | Include the infographic | Video of Aaron’s explanation – audio / visual |
| Build self reflection and self awareness and make that clear | Video introduction of what future discounting is and how to control your own feelings | Gamification – needs to be specific | Adherence not working |
| Instrument – unpacked – time of day | Set goals > this is what future discounting is > tweak goals | Noom example: trade-offs. Options and choices that are still good not black and white | It's not all or nothing |
| Inspiration | Win-win options? | Explain how the app works | Define what ‘cues’ are |
| A little information at the front and then go down the rabbit hole | Make some dummy goals to walk throuhg the app first | Do the words matter? ‘future discounting’ | Some info with the option to dive in further |
| Having the information in different levels (more or less info) | **“investing in your future self” is stronger language** | “time is going to bass so you may as well do something” – Dr. Phil | No context yet |
| Chronic disease – link to experience now | Are you experiencing ___ now? | Option to help with goal setting | “yolo” |
| Future is positioned as a negative not a positive | **Location notification on the gym app for a prize / reward** | Infographic / introduction | In-person combination with health professional |
| ‘expert’ trustworthy person for video intro | Question mark bubble for help – context specific | Audio for accessibility | Feedback when completing survey |
| Feedback r.e. survey over time – encouragement | Timeline – check in at 3, 6, 12 months? | Visual feedback and encouragement | Stepping stones to indicate progress visually |
| Embed in workplace – free apps from work – transferability could be an issue though | Finance – e.g. banking app – show financial goals | Travel apps have trip as a goal. Integrate reminders | Training apps |

**4. Interim recommendations following Workshop 1 series**

| **Recommend?** | **Description** | **Priority L/M/H** | **Comment** |
| --- | --- | --- | --- |
| N | CSIRO branding up front to build trust | L | Can of worms. Revisit |
| Y | Gamification  streaks, badges etc. for regular check-in  celebration for setting and completing goals  option to share accomplishments on social media or via text etc. | H |  |
| **Y** | Dashboard  calendar of completing check-ins / EMAs / viewing prompt  badge or stamp for completion, option for ‘hiding / fixing’ missed days (e.g. duolingo) | M |  |
| Y | Goals presented in list with text-based contextual countdown to 2 week resolution (e.g. “just under 2.5 months left”) | M |  |
| Y | Query integration with other apps? E.g. when hitting step goal, or using a social media app for more than [custom] minutes?, when doing a weigh-in. | L | Report back on resource requirements for this |
| Y | Push notifications  Customisable to time of day and day of week based on personal preference | M | Early bird, night owl, bit of both as options. Visual selection. |
| Y | Ability to edit, reframe, or reset goals and future events (any text-based inputs) | M | At the end of the goal setting, test prompt > confirm or edit. Allow changes for the first 48 hours |
| N | Explore: guided reflection of health conditions in immediate family / friends to prompt importance? | L | Consider including in background context alongside positive elements. |
| Y | Data entry fatigue  A tour / tutorial environment where people are taken through the process of setting goals etc. with 3 x pre-filled goals to select from. | M |  |
| N | Data entry fatigue  AI to take key words or text and translate into a standard prompt sentence format (with correct grammar) | L |  |
| N | Query inclusion of financial goals in future discounting intervention, particularly where these are linked with food choices / PA behaviours | L | Explore what this might look like and where the boundaries might be in workshop 2 |
| N | Inclusion of personal stories – “someone like me” – as a proxy for having a personal connection / family member diagnosis | L | Embed personal story into ‘tour’ environment as ‘humanised’ version |
| Y | Check app logic to avoid looping | H |  |
| N | Introduction narrative – query inclusion of a short animation / link to other pages / info?  Explanation of ‘why’ in context | M | Explore further in Workshop 2. Motivational interviewing? |
| Y | Query: inclusion of images in goals? – upload from device | H |  |
| Y | Avatar – provide option to upload photo of self or choose an avatar / emoji | H |  |
| Y | Lock or add friction to re-entering quiz after completion to avoid looping | H | Popup dialogue if clicked again within 10 mins – “are you sure you wish to do this again?” |
| Y | Add ‘save as draft’ and ‘continue’ functions to partially completed activities | M | Please confirm difficulty of this |
| TBC | Explore different menu / navigation options to make the different sections of the app more clear | H |  |
| N | Develop ‘fun facts’ / ‘teachable moments’ to pop up in response to completing the EMA, goal setting, etc.  Info cards? | L | Additional intervention component, revist if this emerges again |
| N | Explore portal visual metaphor in dashboard – going through the portal to view goals, connect with future self avatar, connect with future | M | Prototype at workshop 2 |
| Y | Query: is it possible to include ability to break goals down into smaller chunks? | L | Provide ability to record notes on how you might have progressed toward the goal / prompt (and access these from the dashboard?) |
| Y | Clarify if goals should be linked or not – potentially explain that both are possible in the app. Visual example. | H | Embed permission to set related or disparate goals as part of the ‘tour’.  Note to app developers: goals and events at all timepoints need to be displayed, not just 12 months |
| N | Query: interest to include picture sorting into ‘future oriented’ / ‘immediate oriented’ to allow people to learn through doing (e.g. picture card activity in workshop 1) | H | Prototype in workshop 2 |
| Y | Shift language to be more positive. ‘Future discounting’ is very negative – taglines such as investing in future self etc. are good. | H | (not for developers) |
|  | Consider language and name of app |  | To explore in workshop 2 |

## 5. Data from asynchronous feedback process

Between the two sets of workshops, participants were asked to test a new version of the app, and to provide feedback and reflections via the Qualtrics platform.

| **How do you rate the experience of using the Future Self 4 Health (version 2) app compared with the previous version?** | |
| --- | --- |
| **Rating** | **Description** |
| Much worse | It doesn't work for me. When I select 'Continue' to enter either the Future Events or Health Goals, it doesn't give me an option to enter any goals, rather it just says 'Great job! You have defining your future events/goals'. Also, when I am then directed to the Ecological Assessment, it does not allow me to complete the survey. |
| Somewhat worse | Access and user ability is difficult **maybe my phone is old** (4-6 old android) when I got to the ""Lets get started"" I chose physical activity and pressed continue however nothing moved, I tried opening and reinstalled the app but got stuck at this point (I do like the new layout, clear and simple colours and pictures)  Then the app also was something yay completed goals and sent me to a survey but it also went nowhere. Also I keep getting notifications to complete morning or evening survey and sends me to a page ""sit tight for a moment"" but still goes nowhere. |
| About the same | It’s an app! |
|  | I couldn’t really tell much difference |
|  | I did not have the opportunity to trial the previous version so I don't have anything to compare it to. |
|  | Did not see version 1 |
| Somewhat better | Looks sleeker, as-you-go instructions |
|  | It seems to be a bit more straightforward, though I would like some prompting examples for the cues statement development. |
|  | There was a lot better information and guidance as to what to do. Much better prompts |
|  | Better because it was clearer what I need to do; but it felt very repetitive, the mood check-ins interrupted me at work or busy times of the day and I didn’t feel my mood depended at all on prompts, but on what happened at work and home. |
|  | I actually get push notifications to complete the mood surveys (and the push notifications are PERSISTENT, to the point of a little annoying because if you miss the timepoint there are multiple notifications until the survey is done) |
|  | It was hard initially to get used to tapping out of the prompt screen. It wasn’t intuitive. |
| Much better | Improved guidance through the goal setting phase and explaining why we are doing each step. More customisation |
|  | There are no interface issues at all that I encountered this time around |

| **Following the provision of a list of updates: Based on this list, and the experience of using the updated app, do you feel that your feedback / suggestions from the first workshop have been addressed?** | |
| --- | --- |
| **Rating** | **Description** |
| Yes |  |
| Yes, but not entirely | Not sure if it's my technology but my phone but app only lets me get so far then the continue button doesn't work. Happy that other new ideas have been implemented |
|  | **Having more of a link between the check-ins and the future events and health goals** |
|  | A bit more instruction on the future event ie mine were something that could happen in 12 months but could just as easily happen next week |
| Some, but not many | The items in the list sound to be good improvements, however, they do not reflect what I have experienced with the new app. |

| **One of the most requested changes was for an introduction to be included in the app to provide context and some guidance. How would you rate the changes made to address this?** | |
| --- | --- |
| **Rating** | **Description** |
| 5 | 😁 |
| 4 | The Intro was helpful |
|  | I skipped the intro haha |
|  | There was generally enough background but I still would like some examples on cues. |
|  | **More context and information was provided. Better explaination of what we were enaging in and what is means** |
|  | The intro was short and clear about future discounting. I do think based on interaction with this version of the app its still not entirely |
| 3 | 99% of what the app does is giving you prompts and asking you to do mood checkins - **there doesn’t seem to be any benefit to the user of doing those checkins** |
|  | it's relatively easy to use, but not 100% intuitive yet |

| **We also made quite a few changes to improve the user-experience within the app. How did we go with making these changes?** | |
| --- | --- |
| **Rating** | **Description** |
| 5 | 😁 |
| 4 | It was easy to navigate |
|  | Much more user friendly, thought he **pop ups were annoying as you couldn’t do anything while it was up** |
|  | The app seems to be running more smoothly with fewer bugs. It seems quiet straightforward in the mood questions. |
|  | **The app is more attention-engaging with the push notifications- I don't recall getting these in the first version.** |
|  | It feels more seamless |
| 3 | **There was much better explanation and it was easy enough to navigate**. Still **feels disconnected setting the future events and health goals without some tangible or clear process to meet them?** |
| 2 | It appears to be worse than I remember from the original. It just isn't functioning. |
| 1 | 😫 |
|  | as previously stated I only got so far into the app and the continue button wouldn't work |

| **A lot of people noticed little bugs and issues with the app first time around. How did we go with fixing these?** | |
| --- | --- |
| **Rating** | **Description** |
| 5 |  |
| 4 | I noticed that there was a space between my sentences in the cues I developed and the full stop that was added. |
|  | No additional bugs that I found |
|  | Just the tapping out of the prompt screen |
| 3 | On first download of the new version there was a considerable lag in the app loading for set up (stuck on the flying person animation) |
| 2 | I mentioned there earlier. Basically, it doesn't seem to be functioning to allow me to enter goals or complete the questionnaire. |
| 1 | 😫 |
|  | The continue button wouldn't work, is my phone to old? It worked okay with the last app |

| **Can you tell us about any additional bugs you've found?** |
| --- |
| I mentioned there earlier. Basically, it doesn't seem to be functioning to allow me to enter goals or complete the questionnaire. |
| I noticed that there was a space between my sentences in the cues I developed and the full stop that was added. |
| No additional bugs that I found |
| On first download of the new version there was a considerable lag in the app loading for set up (stuck on the flying person animation) |
| Just the tapping out of the prompt screen |
| The continue button wouldn't work, **is my phone to old?** It worked okay with the last app |

| **We're almost done! But before you go, is there anything else at all that you'd like to share about your experience with the new version of the app?** |
| --- |
| After reading the bug enhancements that were made I remember they weren’t in the first version so yes the experience was smoother but still lacked a bit of context |
| New version is much better, **I do appreciate being able to edit my cue to something that works for me**, but it only allowed me to do this for one of my cues. Allows you to choose a cue that works best for the way your mind works. |
| It feels like it is a significantly improved app. Is part of the co-design process bringing in people who haven’t used the previous version for their input? I can’t quite remember specifically what I didn’t like about the previous version, just the feeling of frustration when using it, and I didn’t have that with this version. But I am now leaving room knowing it is not a finished product, and I wonder if that’s something a fresh set of eyes would be able to address? My main sentiment with the current version is that the frequent pop ups to complete the survey were a little annoying. Including an option to schedule the cues at an appropriate time may help mitigate this perhaps? |
| Hopefully this is a glitch and I'll get to try out a working version of the app. |
| I’m wondering how the wakefulness/sleepiness qs factor in. Does it matter that I’ve just woken up/ had a long day etc? |
| With the mood questions, I found that sometimes if I was tired or rushing I would misinterpret some questions because I was thinking of it from the perspective of the previous question. I would respond but on the sliding bar would select the opposite end of where I actually meant to if I’d have interpreted the question correctly. |
| Writing the future event and health goals still requires some more guidance in ensuring they are effective prompts on a daily basis |
| Pretty straight forward |
| When I go into the app now it just says "sit tight for a moment" and that is all |

## 6. Data from Workshop 2 series

## Explaining future discounting to a friend

The elements below were developed as starting points for explaining future discounting as a concept and could be developed further into introductory materials if desired.

| **Narrative** | **Examples** |
| --- | --- |
| “Make good choices now to enjoy the benefits later” | 1.1.1; 1.1.2; 1.1.3; 2.1.1; 2.1.3; 2.1.4; 3.1.3; 4.1.2; 4.1.4 |
| “Making the connection between present you and future you” | 3.1.1; 2.1.4; 3.1.2 |
| “Shorter term future, small positive changes” | 3.1.5; 4.1.1 |
| “Being able to keep doing the things you enjoy – what makes you happy? | 4.1.1; 4.1.3 |
| “Planting a seed – nurturing” | 3.1.4 |
| "Comparison – two people / scenarios” | 2.1.2 |
| “Building will power” | 1.1.4 |
| “Goal setting” | 4.1.3 |
| **Content Focus** | **Examples** |
| Diet | 2.1.1 |
| Exercise | 3.1.3; 3.1.5 |
| Diet and exercise | 3.1.1; 3.1.4; 4.1.3 |
| Diet, exercise, and personal care | 1.1.1 |
| Diet, exercise and environment | 1.1.2 |
| Diet, exercise and well-being | 3.1.2 |
| Diet, exercise and financial | 4.1.4 |
| Financial, applied to health | 4.1.2; 2.1.1 |

For full stories, see appendix I.

## Barriers to engaging with a future discounting intervention

This activity was conducted in only 3 of the 4 (2nd round) workshops due to time limits. Where barriers were discussed, obstacles and ideas for resolution were offered. Some themes emerged. These are listed below.

|  | Workshop  18 Mar 2024  CBD  (number of references) | Workshop  20 Mar 2024  CBD  (number of references) | Workshop  22 Mar 2024  Christies Downs  (number of references) | Workshop  27 Mar 2024  CBD  (number of references) | Total  (number of references, all workshops) |
| --- | --- | --- | --- | --- | --- |
| Stress and busy schedule | - | 3 | 4 | 0 | 7 |
| Effort, level of personal investment required | - | 1 | 0 | 5 | 6 |
| Aversion to constraint or denying oneself | - | 0 | 6 | 0 | 6 |
| Difficulty navigating unfamiliar or complex tasks | - | 0 | 5 | 0 | 5 |
| Fear of failure and memory of past failures | - | 0 | 4 | 0 | 4 |
| Cost (financial) | - | 1 | 0 | 3 | 4 |
| Personal differences, background | - | 0 | 3 | 0 | 3 |
| Technology | - | 0 | 0 | 3 | 3 |
| Waning interest in fitness apps over time | - | 0 | 0 | 3 | 3 |
| Trauma/adversity | - | 3 | 0 | 0 | 3 |

| **Stress, and juggling conflicting demands** | | |
| --- | --- | --- |
| **What are all the other things that I should be doing?** | I haven’t got the mental space right now and that phone is annoying me, alert, alert | There is a gender difference here too. |
| When my routine is disrupted, I know what I need to do… it just takes forever to get back on track. | Trying to pick from the list of 'shoulds' | I didn’t get a break until 1.30. |
| Being a new mum |  |  |
| **Level of personal investment required** | | |
| This app requires a huge amount of ownership by someone using it. | **Effort, mental effort** | Requires a lot of personal investment. |
| Effort cost in terms of just putting it on the device in the first place. | It is hard constantly going, this is my goal, this is my intention, breaking that down into action steps. | **Time** |
| **Aversion to constraint** | | |
| People who are highly motivated, highly organised, these are not nice people. | It feels like I am denying myself. | **I don’t really want to see myself as a person who is planning like that** |
| You don’t want to be the type of person who denies themselves. | I don’t like thinking that I am this person that wakes up at 6 o’clock in the morning, I don’t think I am a robot. | There is some benefit to living in the now, for mental health and that type of thing. |
| **Difficulty navigating unfamiliar or complex tasks** | | |
| Sometimes you know what you want to do but you can’t envisage the steps to get there. | **it just never happens because you can’t think what you need to do.** | not being able to decide |
| Inability to prioritise can be a big barrier, that overwhelm feeling. | ‘I want to do this’ but you don’t have the mental capacity to do what you need to do to get there. | you have no idea how to get from A to B |
| AI tool called Goblin tools. You put in something you want to achieve, and it gives you a step by step. | **A role a mentor could help with** |  |
| **Fear of failure** | | |
| **It is scary to do things and easier sometimes not to engage.** | Memory of past successes followed by failures | **I think, how long will I last? Will it be one day? One week?** |
| The memory of these experiences hold you back. | you can start the diet but then you give up. | I think about moments when I was really active, but then after that I wasn’t active |
| **Cost - financial barriers** | | |
| **Healthy food is expensive** | if there is a price associated with using the app | **Cost - $** |
| **Personal differences, background** | | |
| Personal differences in motivation, particularly following different upbringings | Eventually I will do it but it is a bigger hurdle for me to cross to get there. | I grew up in a family that didn't really do that. |
| **Technology** | | |
| **Need to have a smartphone** | Lots of elderly people have brick phones. | Some people don’t even know how to install an app. |
| Option to switch off  **Trying hard to reduce phone usage for health** |  | **Notifications – too frequent.** Option to choose – set notification times |
| **Waning interest in fitness apps over time** | | |
| At the start it was a really good motivator. | Get into it and then when I set those habits, I petered off. | **I don’t need it anymore.** |
| **Trauma or adversity** | | |
| traumas or experiences where people find it hard to think about the future. | Domestic violence | if their partner has died, or they can’t have a baby, if they lose their job |
| Not the time for it… when they’ve got pressures of the here and now. | **Too hard to even think about the future** |  |

## Visual metaphors

| **Theme** | **Like**  (number of references) | **Don't Like**  (number of references) |
| --- | --- | --- |
| Grow a tree | 24 | 5 |
| Stepping stones | 22 | 3 |
| Road | 20 | 8 |
| Advent calendar, doors, windows | 18 | 4 |
| List | 11 | 10 |
| Graphs, abstract data visualisation | 11 | 7 |
| Seasons | 9 | 5 |
| Avatars | 8 | 1 |
| Adjusting a date | 7 | 8 |
| Through a portal | 6 | 4 |
| Islands | 2 | 6 |

| **Grow a tree (Like)**  The most popular metaphor across the workshops was growing a tree. This connected with the participants in the following ways:   - **Nurturing/Parallel with personal growth**, to care for something and see it thrive as a positive metaphor for self-care with resulting personal nourishment and growth. - **Time and change in time**. The outward appearance of a growing tree changes slowly but steadily with time. Therefore, the stages of tree growth presented visually imply passage of time. - **Investment of time/Perseverance**. To care for the needs of a growing tree takes time and perseverance. - Nature aesthetic/Reward. The aesthetic of trees and nature is appealing and the observing changes in growth is rewarding. - **Further suggestions**. | | | |
| --- | --- | --- | --- |
| **Nurturing/Parallel with personal growth** | | It’s quite nurturing. | Like nurturing your future self. |
| Plant, water, fertilise it. | Like a Tamagotchi. | If you achieve one of the intentions → growth | If you are the tree that would make sense. |
| **Time and change** | | | |
| A small tree at 3 months, a medium tree at 6 months, and a big tree at 12 months; that would be helpful. | Can also have the seasonal changes embedded in that as well. The changes the tree will go through. | I like it. A tree takes a long time to grow – and that is like there is something bigger in the future. | I like the acorn seedling, progression-in-time. That’s really powerful, I think. |
| **Grow a tree (Don’t like)** | |  |  |
| I would need more frequent rewards – like flowers or a garden. | An extra responsibility, an onerous task. | Trees can only get so high, so if you are continuing this app for some time - beyond the 12 months – your tree can’t get any bigger in the end. | It makes me think of all the plants I have killed. |

| **Stepping Stones (Like)**  Another popular metaphor, the stepping stones resonated with participants in the following ways:   - **Achievable** - **Different pathways, linked but not linear** | | | |
| --- | --- | --- | --- |
| **Achievable** | | | |
| I like stepping stones, its little steps to get there. Little things you can do. | I love it. It is exactly what I am thinking about. You still have the road but it is little steps. | Small steps – makes things achievable. | You can fall off the stepping stones but you can get back on. |
| **Different pathways, linked but not linear** | | Sequence but with detours | Different pathways but there is a general direction. |
| It connects with the windows, because as you move along the stepping stones there are different options. You can skip some. | It is quite a nice metaphor, that there is not necessarily one way to get to the goal. There could be multiple ways. | Journey, more linked than the islands | You can skip a rock, can choose a different path. |
| Rewards along the way | Road but with points. It blends windows with roads. | Some rocks can be bigger than others. | The stepping stones is similar to the road but with more defined ‘blobby’ points. So you can have the 3 month goal, the 6 month goal and the 12 month goal, and beyond that if you want. |
| **Stepping Stones (Don’t like)** | | | |
| But what is connecting them? How do people get there? Are they linked or not linked? | Tiring | With the ‘blobby’ stepping stones, how are you imagining people getting between them. Are they meant to leap? You have nothing in between. | Are they linked? How are they expected to get there? [It depends if the goals are meant to be connected or not]. |

| **Road (Like)**  The road was a popular metaphor, with support falling in roughly two groups:   - **Journey/Adventure**, where the twists, turns and detours of the journey from now to the future are captured/presented. Here, choice and chance are prominent. - **Progress/Direction**, where a more linear path is imagined, tracking progress to a known goal. | | | |
| --- | --- | --- | --- |
| **Journey/Adventure** | | | |
| Capturing the idea of a journey between now and the future. | I like that it is not a straight path and that there are points along the path, like milestones. An opportunity for reflection, how am I going towards this goal? | Maybe have the ability to change (a fork in the road?) because you want to see a straight path but it doesn’t always go that way. | The option to take the ‘wrong’ path, or detour, and then retrace your steps. |
| Choose your own adventure. | I really love roads or journeys that show dips and reversals to remind you that it isn’t necessarily a linear trajectory. | Or have a safe spot? A picnic spot. | With the road, you can always add a longer road, you can veer off. You have the opportunity to keep going. |
| Could have little points along the way, even if it is just one of your goals. | Maybe a pathway through a forest, something a bit less industrialised. A pathway in nature, rather than a road. | The idea of an intersection is quite nice to capture that whole future discounting thing. Like, one road bad path, bad path, one road leads to greatness. | With detours. |
| **Progression/Direction** | | | |
| A good way to track progress if your goals are connected. | For me, it is a powerful metaphor. When you see the road, it has direction, you see where you are going. You feel confident you will get there. You are going the right way. | I see a road going up different summits. So, you’ve got three summits. The tallest one represents the 12 months, that’s the furthest away. Levels, yeah. | Progression, you’re moving. |
| An opportunity to take a snapshot of your health at the start, and then compare at 3, 6, 12 months. | Gamification – building your skills, progressing. | Makes me feel like they need to be connected, that they build on each other. |  |
| **Road (Don’t Like)**  There were also some criticisms of the road metaphor. These were centred around the following:   - **Too connected and linear**, where 3-, 6- and 12-month goals that are not at all connected, or where plans change, the road is a poor fit. - **Never-ending**, with a road suggesting tedious endlessness. | | | |
| **Too connected and linear** | It makes sense if those three goals are all linked but if people have chosen disparate goals, it’s a problem. | 3/6/12 months might not be connected | What if you want to take a detour? Retrace your steps? It’s not always linear, how you feel might change over time. |
| **Never-ending** | I think a road’s got to end, though. Does it just keep going? The never-ending road… | ‘Are we there yet?’, ‘When are we stopping to pee?’ |  |

| **Road** | **WS2.1** | **WS2.2** | **WS2.3** | **WS2.4** |
| --- | --- | --- | --- | --- |
| I like… | Choose your own adventure  Gamification | Makes them seem connected  Dips and reversals - detours | Detours  See progress  Baseline to start, see if changes  Connects – good way to track direction, confident you will get there (you are going the right way)  Moving  Could have little points along the way – like not a straight path  Points on the path | Progression/movement  Levels/elevations  Intersections |
| I don’t like… | Retrace  What if you want to take a detour – maybe have the ability to change  Or have a safe spot  Streak ‘freeze’ |  | 3/6/12 might not be connected | Doesn’t work if not linked  Never-ending |
| Would work for… |  |  |  |  |

| **Advent calendar, doors, windows** | **WS2.1** | **WS2.2** | **WS2.3** | **WS2.4** |
| --- | --- | --- | --- | --- |
| I like… | But also a path to see where you’ve been – combined calendar – visual  3, 6, 12 months | Can be more discrete  To have all three motivating  Choose your own adventure  Lots of doors/windows  Random | Other things than just 3, 6, 12  Choice of different decision, don’t have to do it in order  Could generate with gen ai but with the option to edit  Reward but just today, not road | Reveal/conceal  Building/growth |
| I don’t like… |  | Maybe harder to navigate  Prefer to just have one (not 3, 6, 12) |  | Chocolate association |
| Would work for… |  |  |  |  |

| **Islands** | **WS2.1** | **WS2.2** | **WS2.3** | **WS2.4** |
| --- | --- | --- | --- | --- |
| I like… |  |  |  | Maybe dotted lines |
| I don’t like… | I don’t like this so much  Too separate | No. Doesn’t resonate. Isolating. Not connected. | Crazy |  |
| Would work for… |  |  |  |  |

| **Seasons** | **WS2.1** | **WS2.2** | **WS2.3** | **WS2.4** |
| --- | --- | --- | --- | --- |
| I like… | I don’t mind this because maybe you would change your goal | Seasons of life  Cyclical  Seasonal playlists – my year segmented |  | Visual distinction  Might be easier to identify barriers  Change – weather makes an impact |
| I don’t like… | Might be more divisive  Maybe not as motivating  I don’t think about the temperature when thinking about the future | My life compartmentalised by seasons |  | Locks it into a time period |
| Would work for… |  |  |  |  |

| **Portal** | **WS2.1** | **WS2.2** | **WS2.3** | **WS2.4** |
| --- | --- | --- | --- | --- |
| I like… | Games  You could be anywhere  Time and space travel |  | Merge with window  Vision of the future  Zoom into screen |  |
| I don’t like… |  | Jumping time - disconnected | Too scientific  Disconnected – skips the journey | Disconnection |
| Would work for… |  |  |  |  |

| **Stepping stones** | **WS2.1** | **WS2.2** | **WS2.3** | **WS2.4** |
| --- | --- | --- | --- | --- |
| I like… | Feels like progress  Going forward  Nature – nice | Small – makes things achievable  Journey  More linked than the islands  Sequence  Detours  2^nd^ large stone, right from the ocean  Prefer natural  (compare with stairs and ladders – too organised) | Rewards along the way  Road but with points  Blends windows with roads  You can skip a rock, can choose a path | Little steps, things you can do  Combine with islands  Doesn’t have to be linear  Multiple ways |
| I don’t like… | Tiring |  |  |  |
| Would work for… |  |  |  |  |

| **Grow a tree** | **WS2.1** | **WS2.2** | **WS2.3** | **WS2.4** |
| --- | --- | --- | --- | --- |
| I like… | Nice – flowers  A tree takes a long time to grow  Nurturing  Plant, water, fertiliser | If you achieve one of the intentions -> growth | Respond to cue → water plant | Resonates as a metaphor for bahaviour change – patience  Investment up front but gets easier with time  Changing colour with mood |
| I don’t like… |  | Too connected for the concept | Would need more frequent rewards – flowers/garden  Extra responsibility |  |
| Would work for… |  |  |  |  |

| **List** | **WS2.1** | **WS2.2** | **WS2.3** | **WS2.4** |
| --- | --- | --- | --- | --- |
| I like… | I love crossing things off  Could combine with e.g. portal  Marking off  Accomplishment |  | As an optional extra | Checking off, satisfying  Record  Motivator |
| I don’t like… | More clinical | Too formal/clinical  Not fun/engaging  Need images  Doesn’t help visualise | Turns into an onerous task  I prefer visual – pictures | What of? Future events? |
| Would work for… | Achievement oriented people |  |  |  |

| **Graphs, abstract data visualisation** | **WS2.1** | **WS2.2** | **WS2.3** | **WS2.4** |
| --- | --- | --- | --- | --- |
| I like… | I love stats  Progress comparison – have a person walking up the graph | Barometer – how you’re feeling  A floor on it though – not down below | Visual data  Progress over time, e.g. health measures |  |
| I don’t like… | Not sure how it would work here | Emotional connection lacking  Love a graph but can’t see how it links here  Too much concentrates on path | Not really for non-measurable goals  Other apps do that and this is more focused on mental rather than tracking actions | What of? Mood data? Engagement?  Passive |
| Would work for… |  |  |  |  |

| **Adjusting a date** | **WS2.1** | **WS2.2** | **WS2.3** | **WS2.4** |
| --- | --- | --- | --- | --- |
| I like… | What month is 3, 6, 12 |  | I prefer to have a date  If there isn’t a date how will I know when entering the app down the track |  |
| I don’t like… |  | Countdown effect  Pressure  Too many variables out of our control  Temporality that you have no control over | Makes it more scary  Too definitive  Might feel like failure, if get to date and it hasn’t happened | Sense of failure if don’t meet it |
| Would work for… |  |  |  | Deadline preferers |

| **Avatars** | **WS2.1** | **WS2.2** | **WS2.3** | **WS2.4** |
| --- | --- | --- | --- | --- |
| I like… | Seasons – fun, animate for fun  Mascot | More wrinkles  Different outfits – dressing her for her activity  c.f. ‘not a nail biter’ |  |  |
| I don’t like… |  |  |  | Never understood |
| Would work for… |  |  |  | Younger demographic  Online community - sharing |

## Icon Sorting

Nine worksheets were completed, with 72 icons in each pack sorted to either Future Reward or Immediate Reward. (See appendix). Although participants moved icons that were ambiguous or did not resonate, all icons were used at least once across the nine worksheets.

| Icons sorted to Immediate Reward only or Future Reward only | | | |
| --- | --- | --- | --- |
| Assigned to Immediate Reward only | Number of times | Assigned to Future Reward only | Number of times |
| 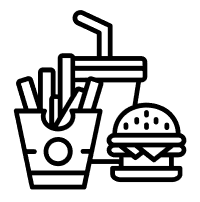 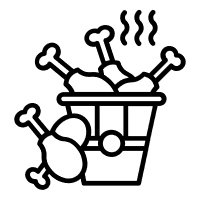 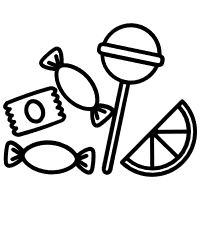 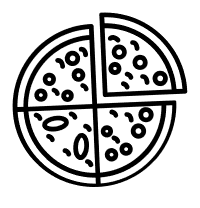 | 9 | 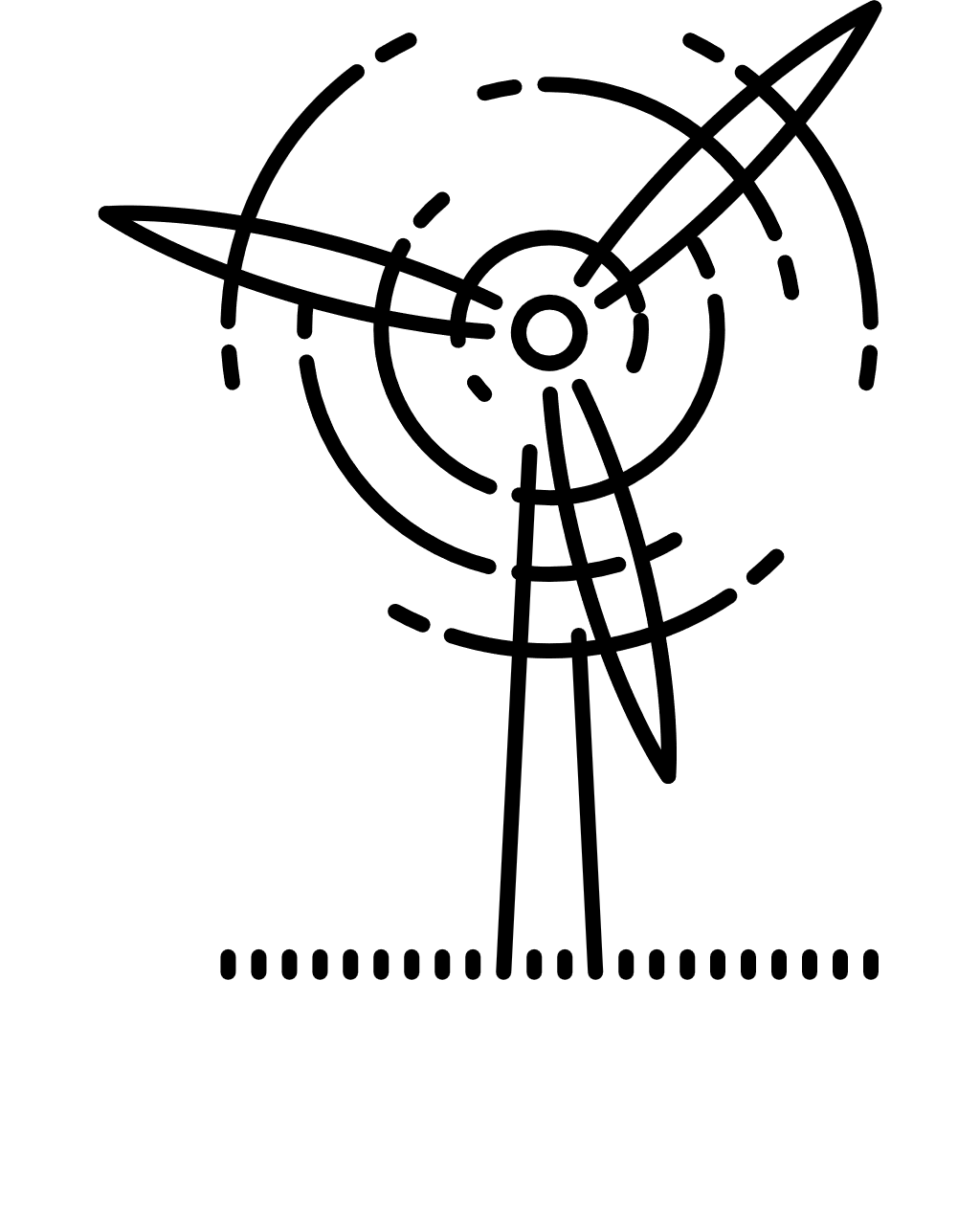 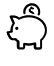 | 9 |
| 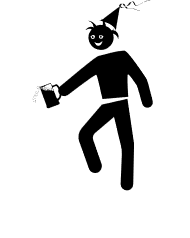 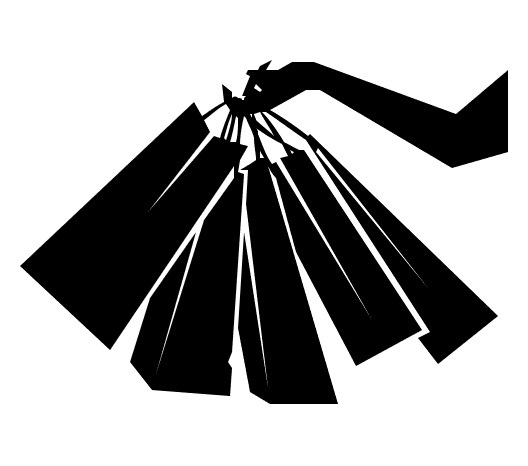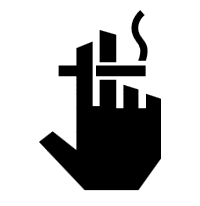 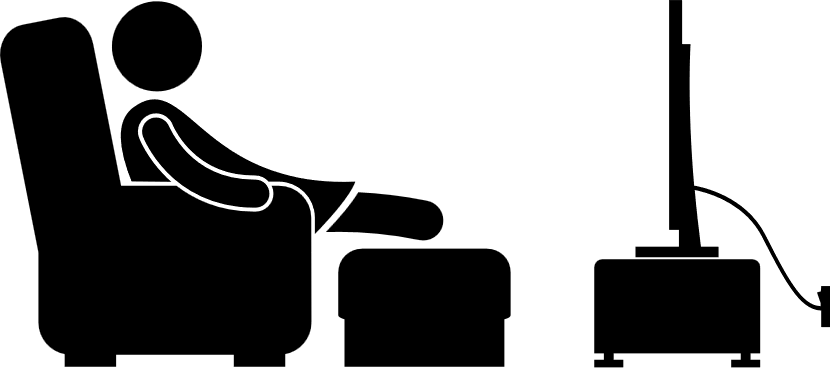 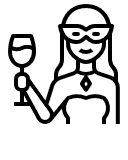 | 8 | 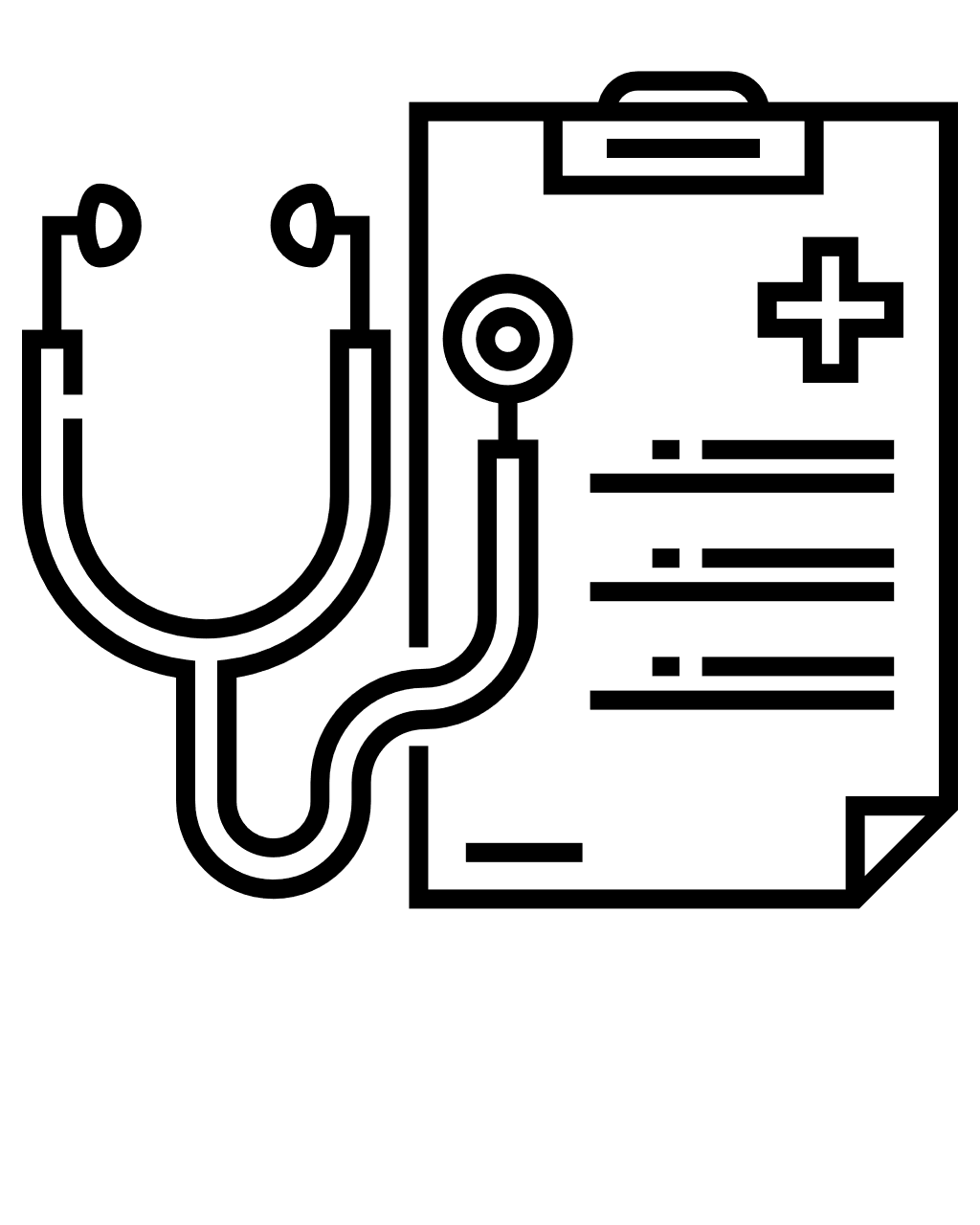 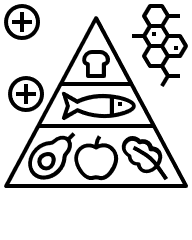 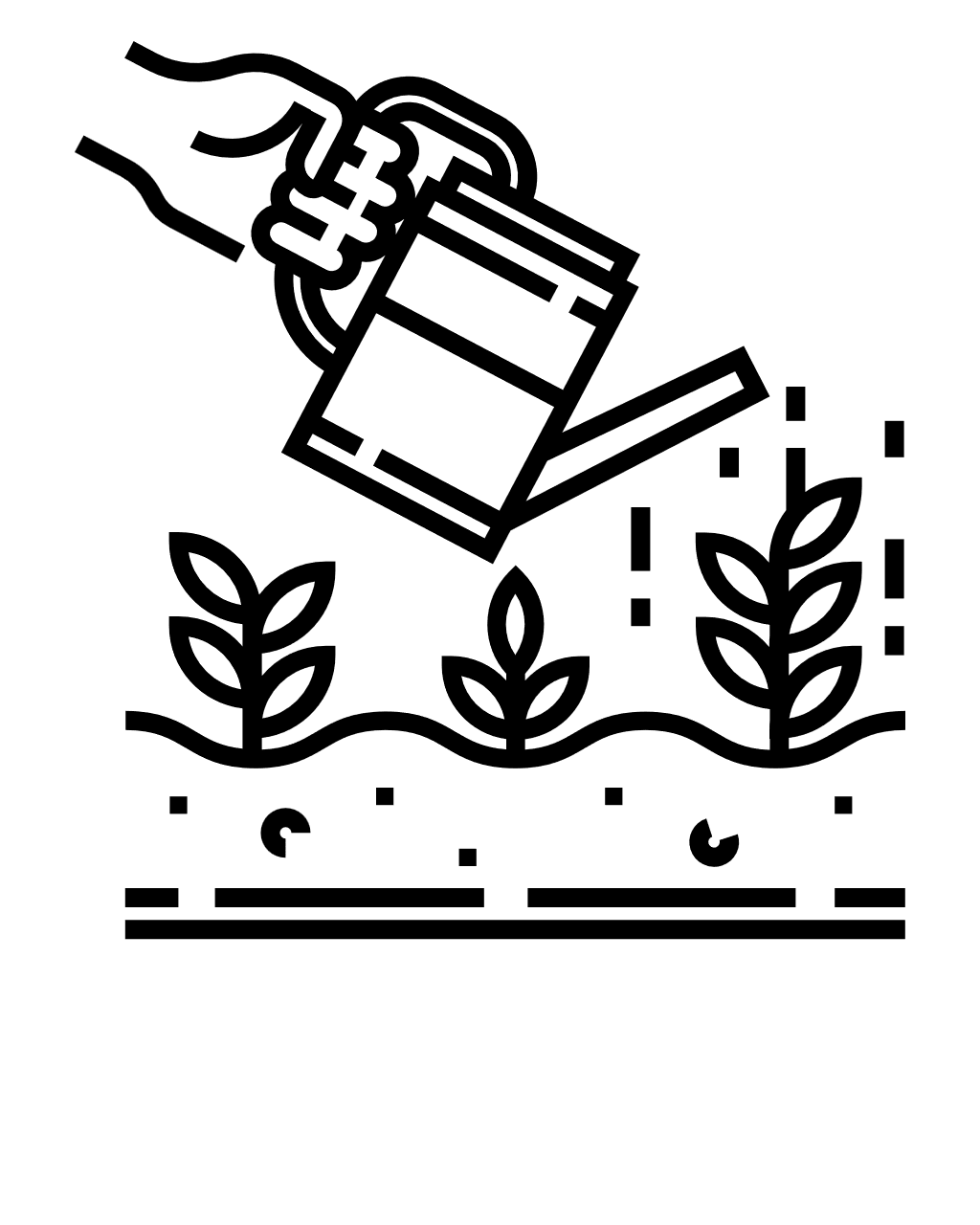 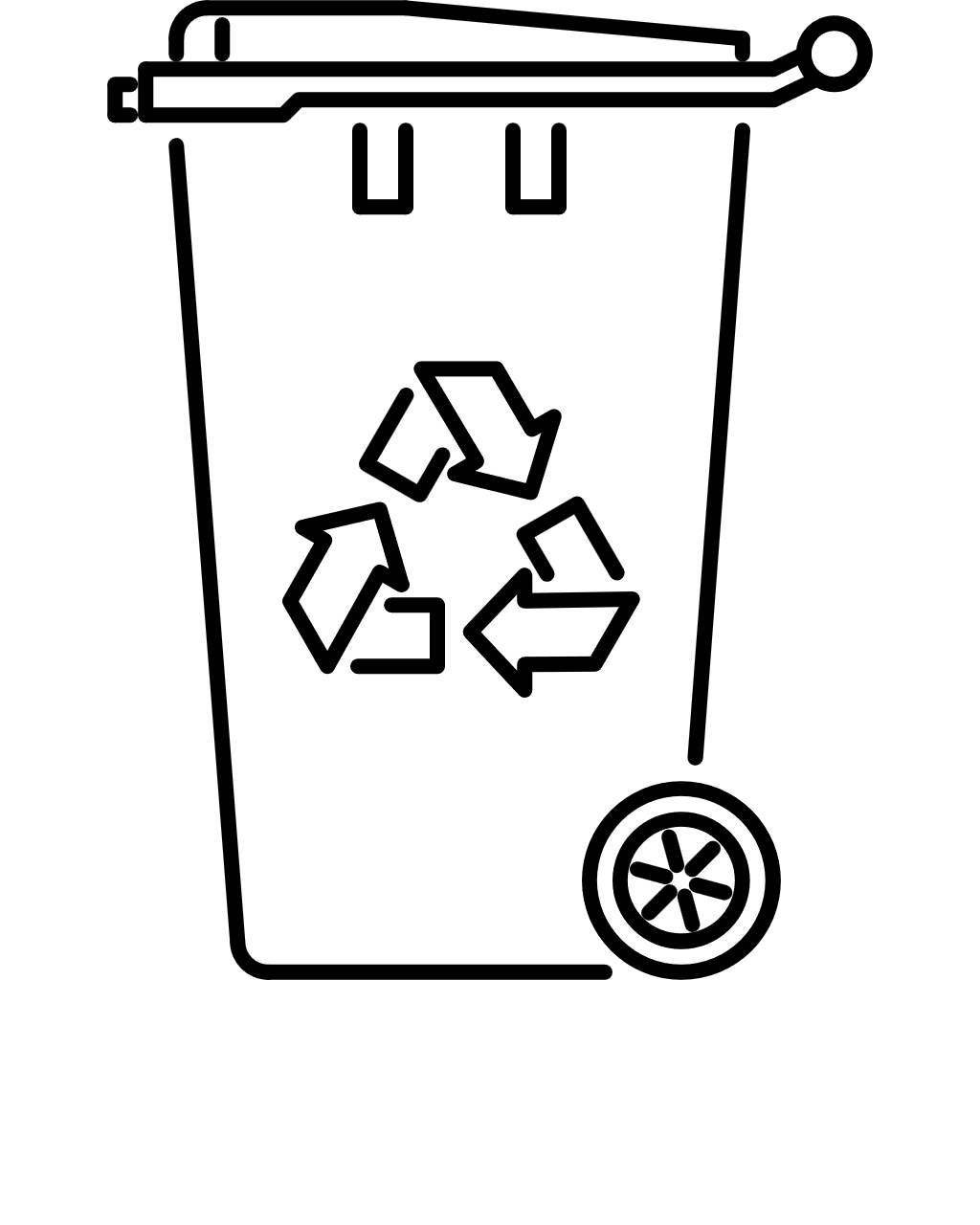 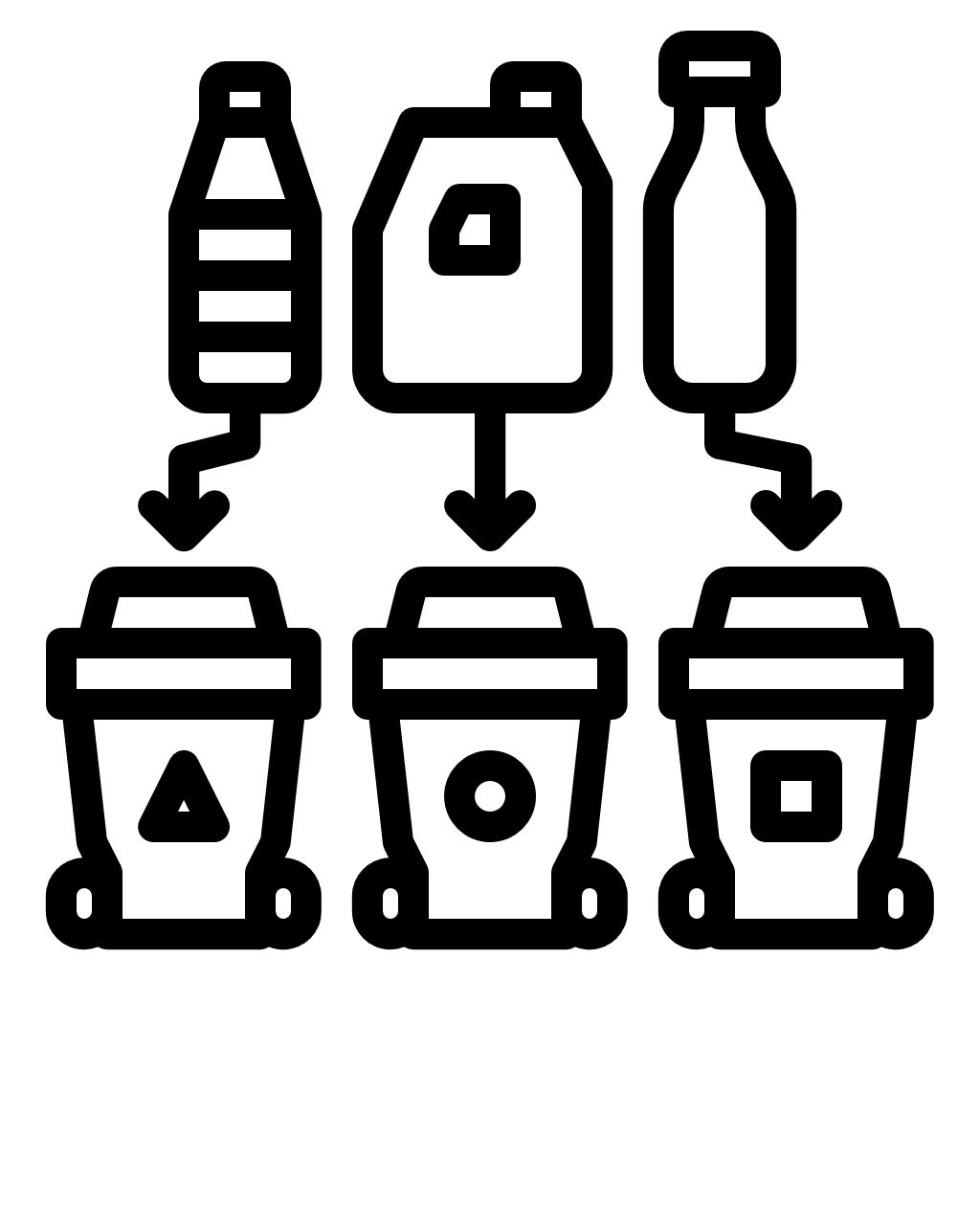 | 8 |
| 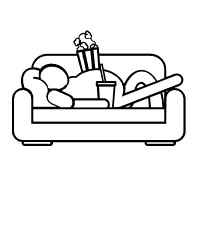 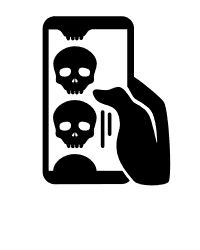 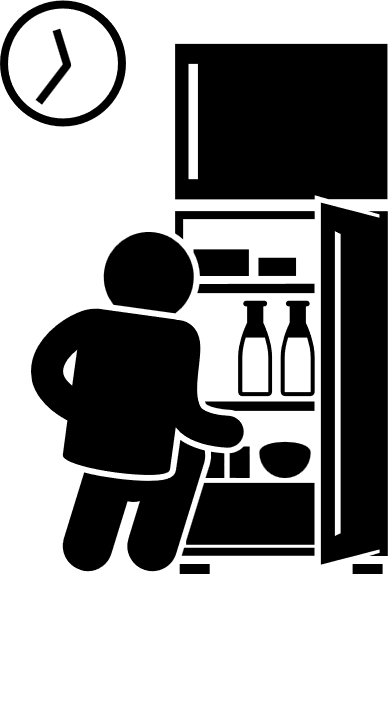 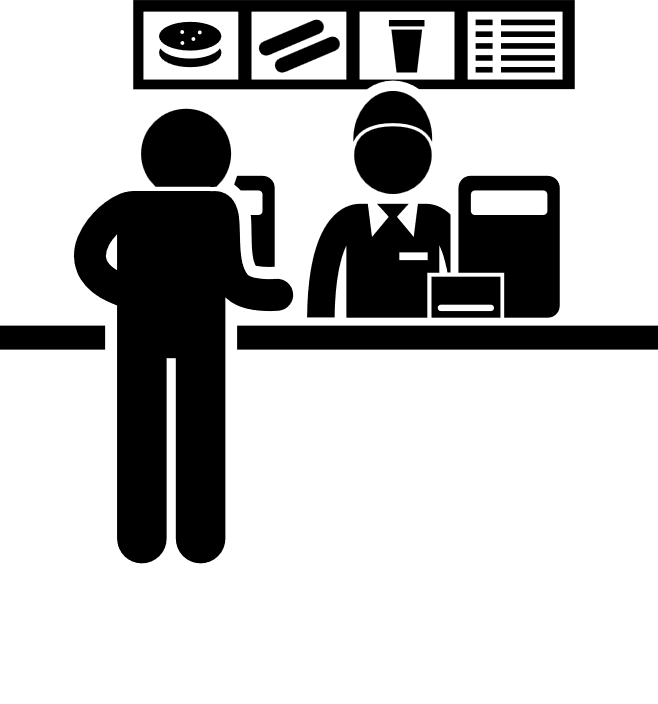 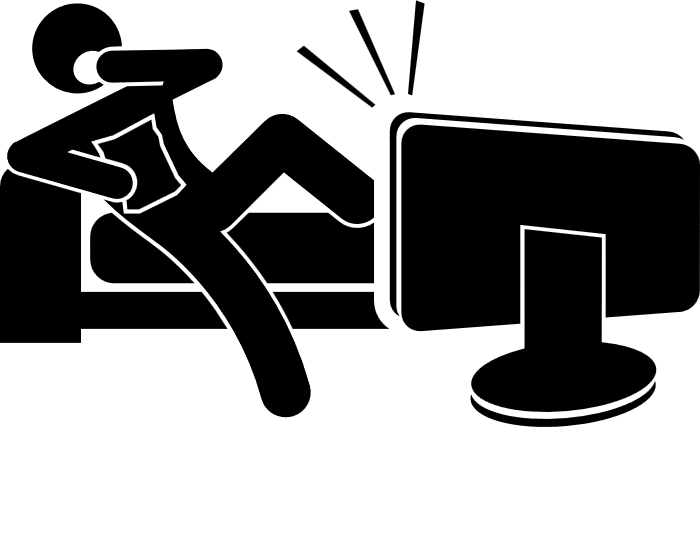 | 7 | 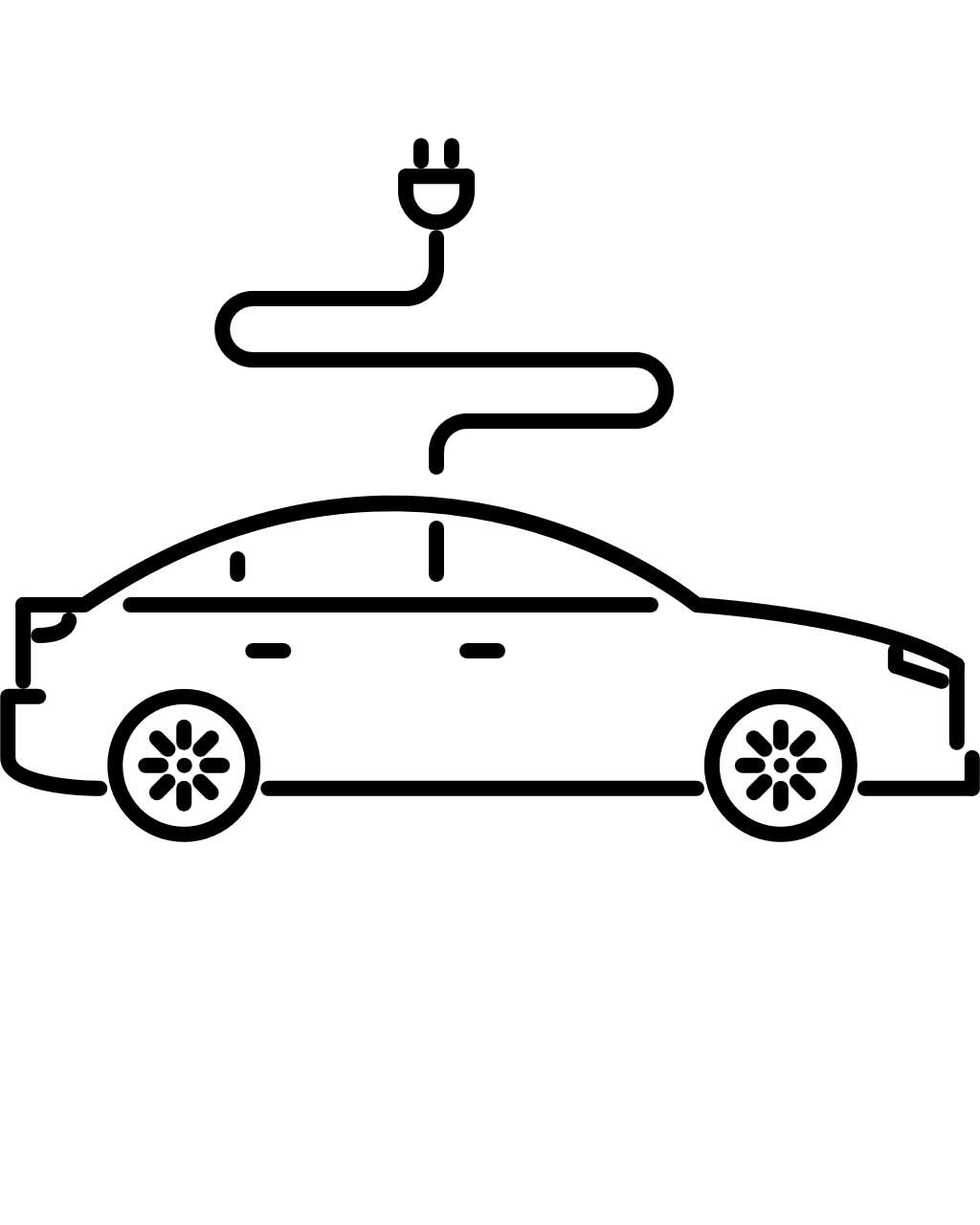 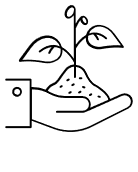 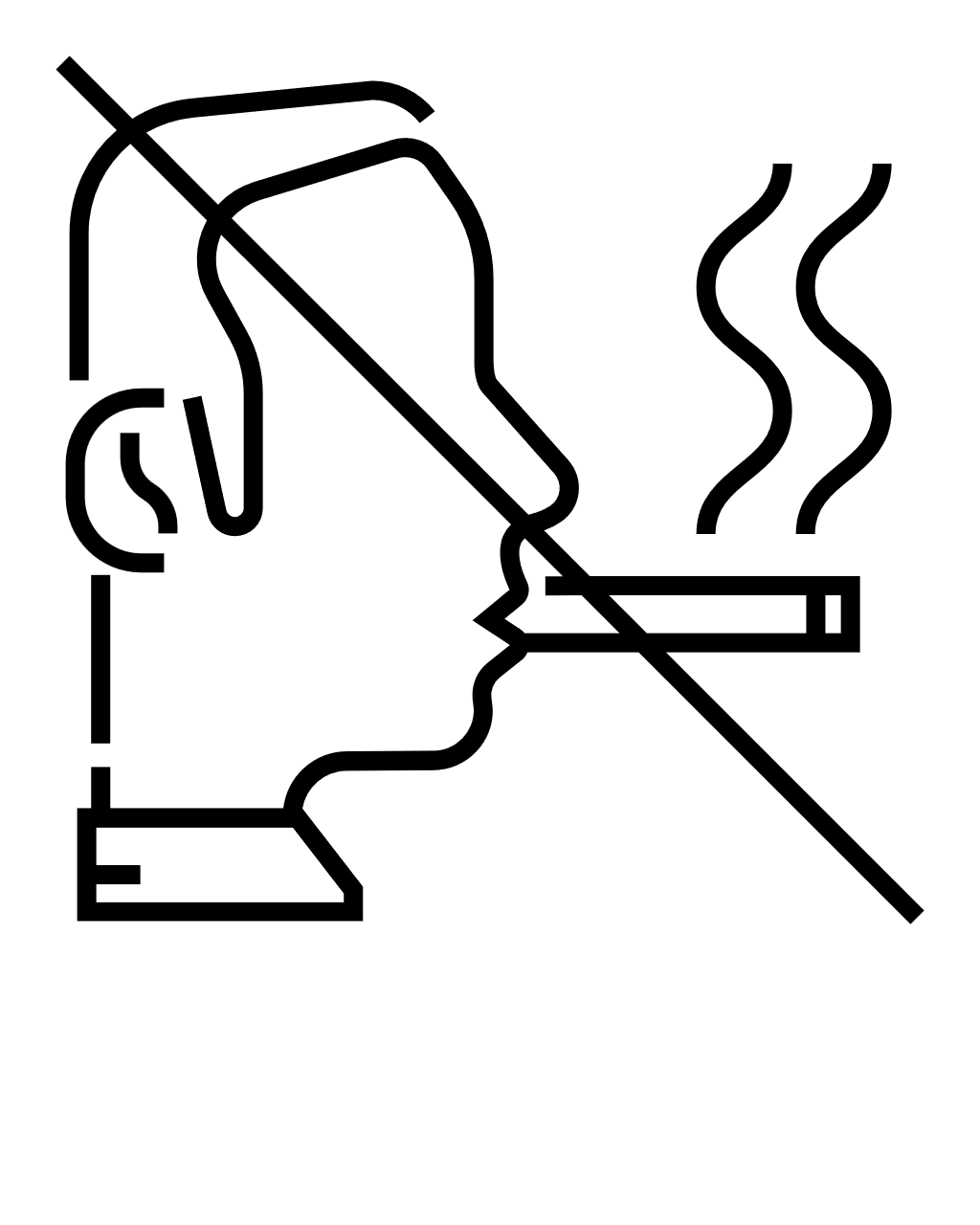 | 7 |
| 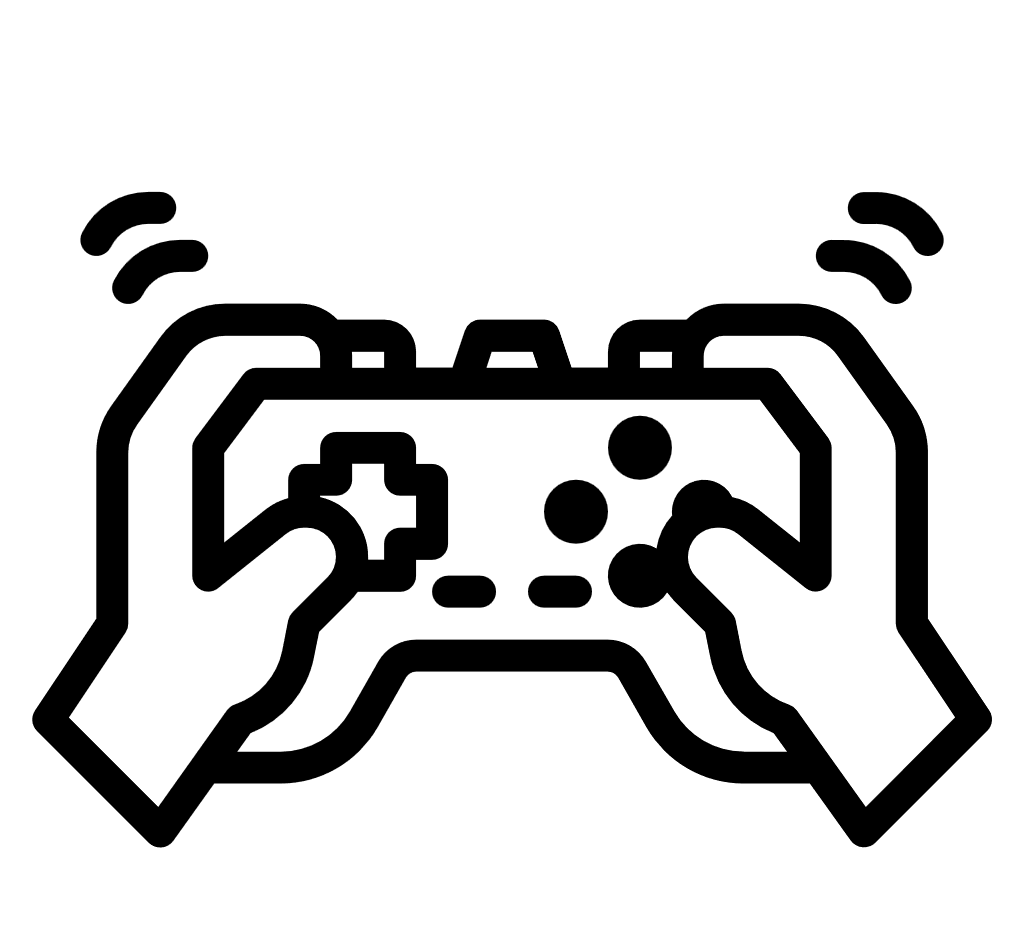 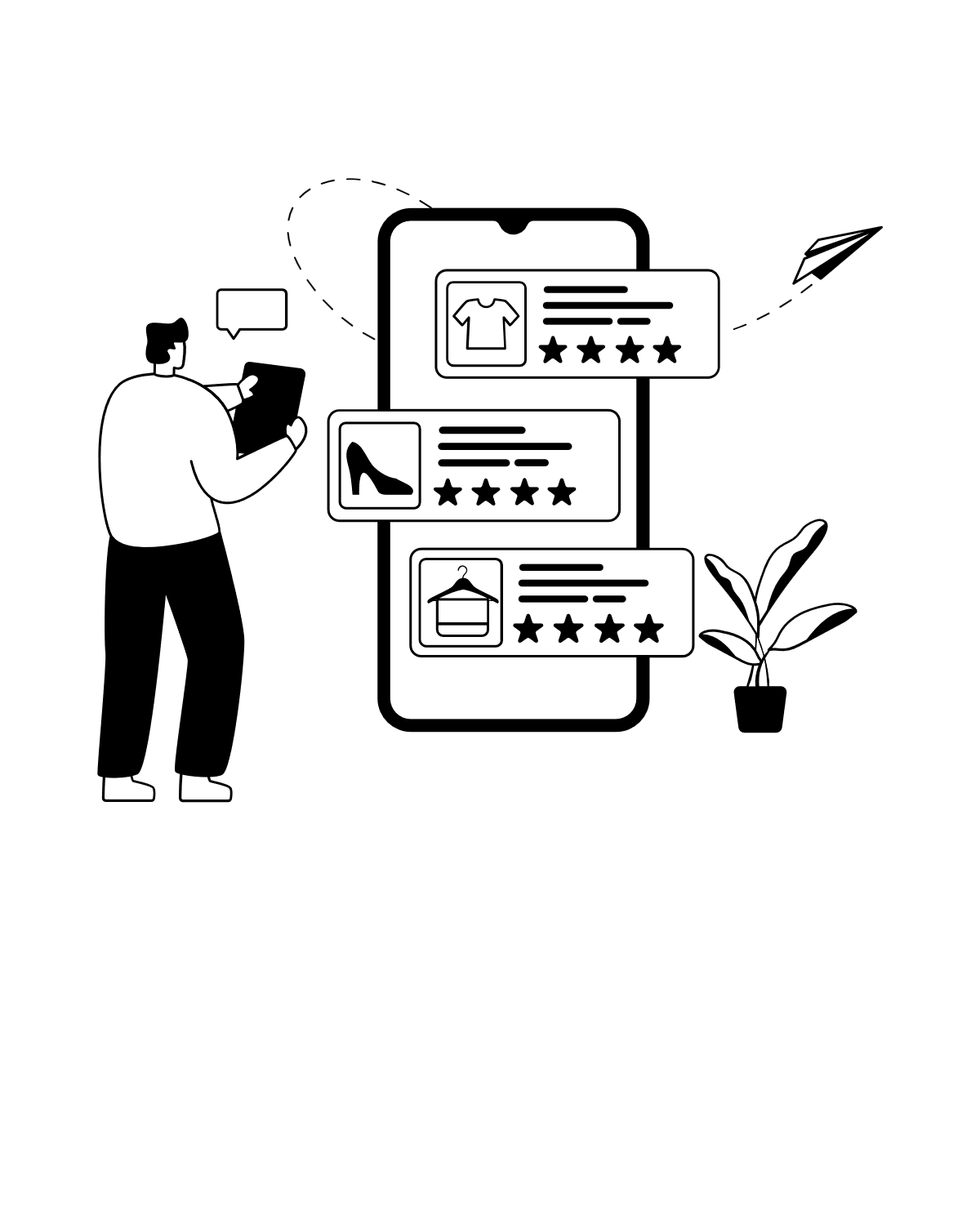 | 6 | 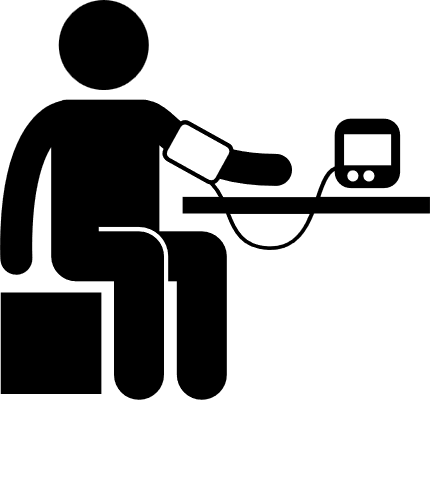 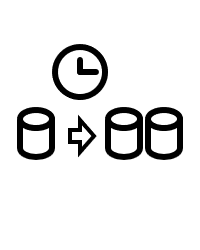 | 6 |
| 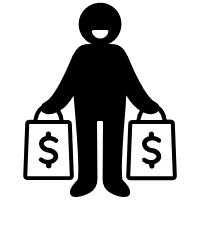 | 5 | 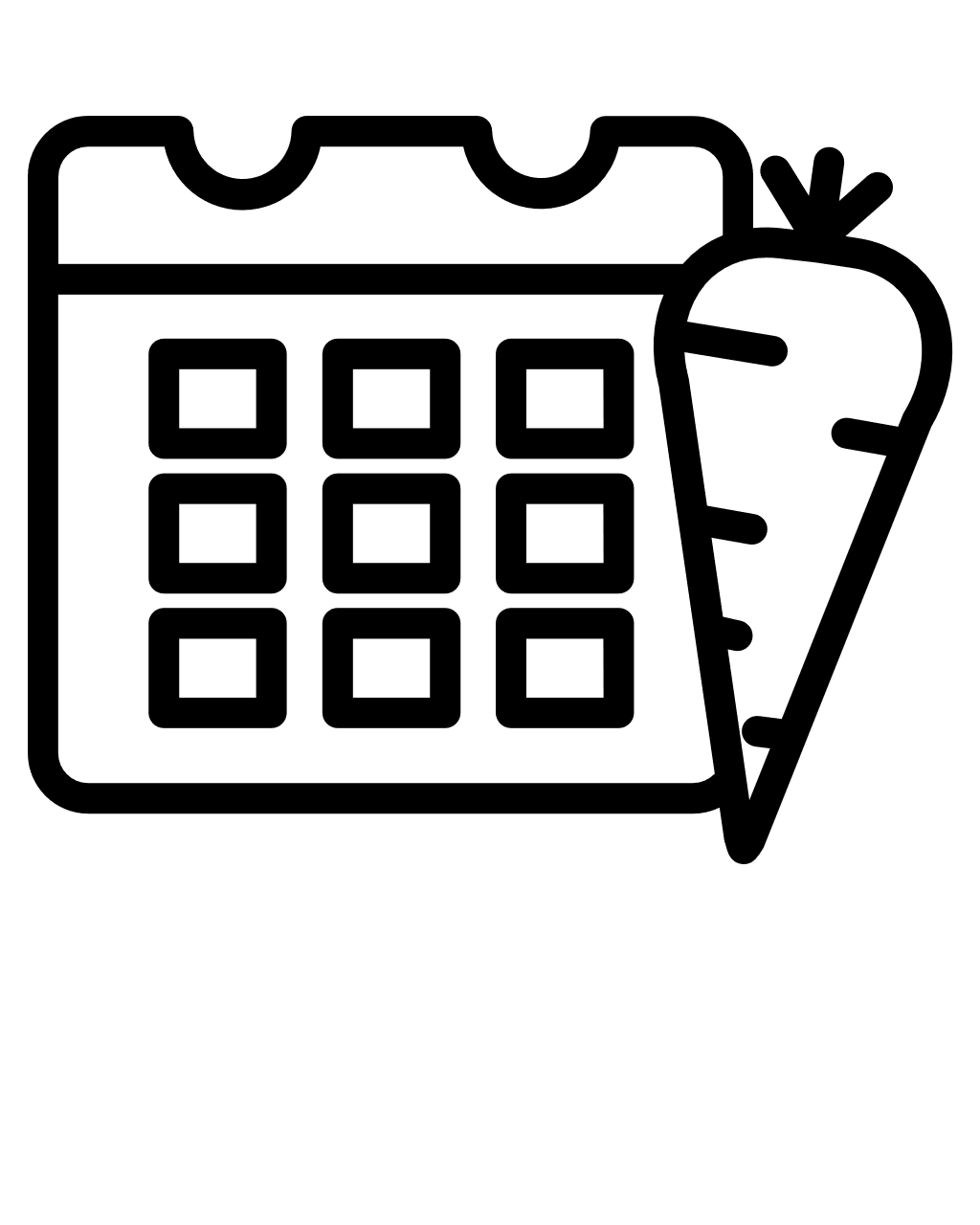 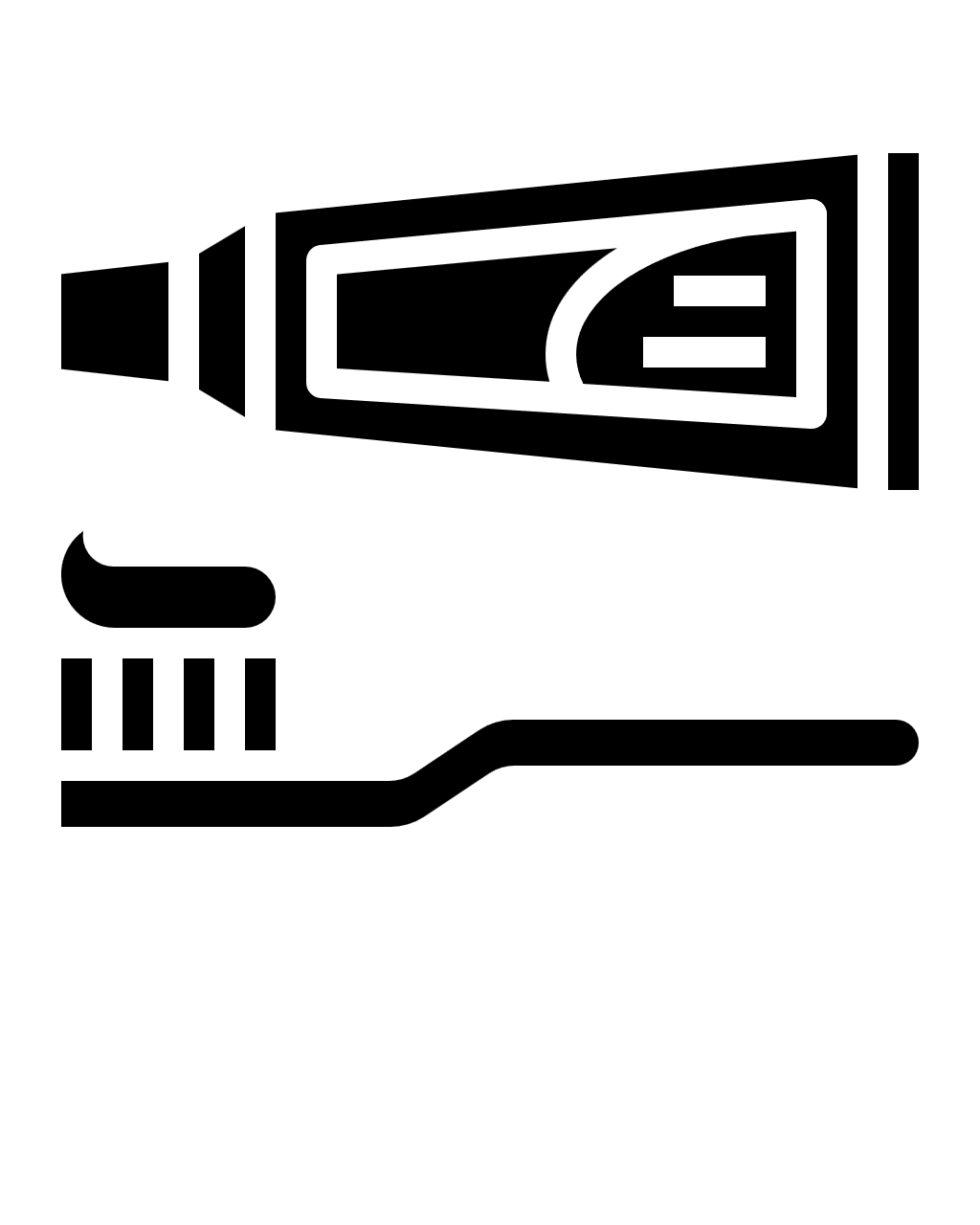 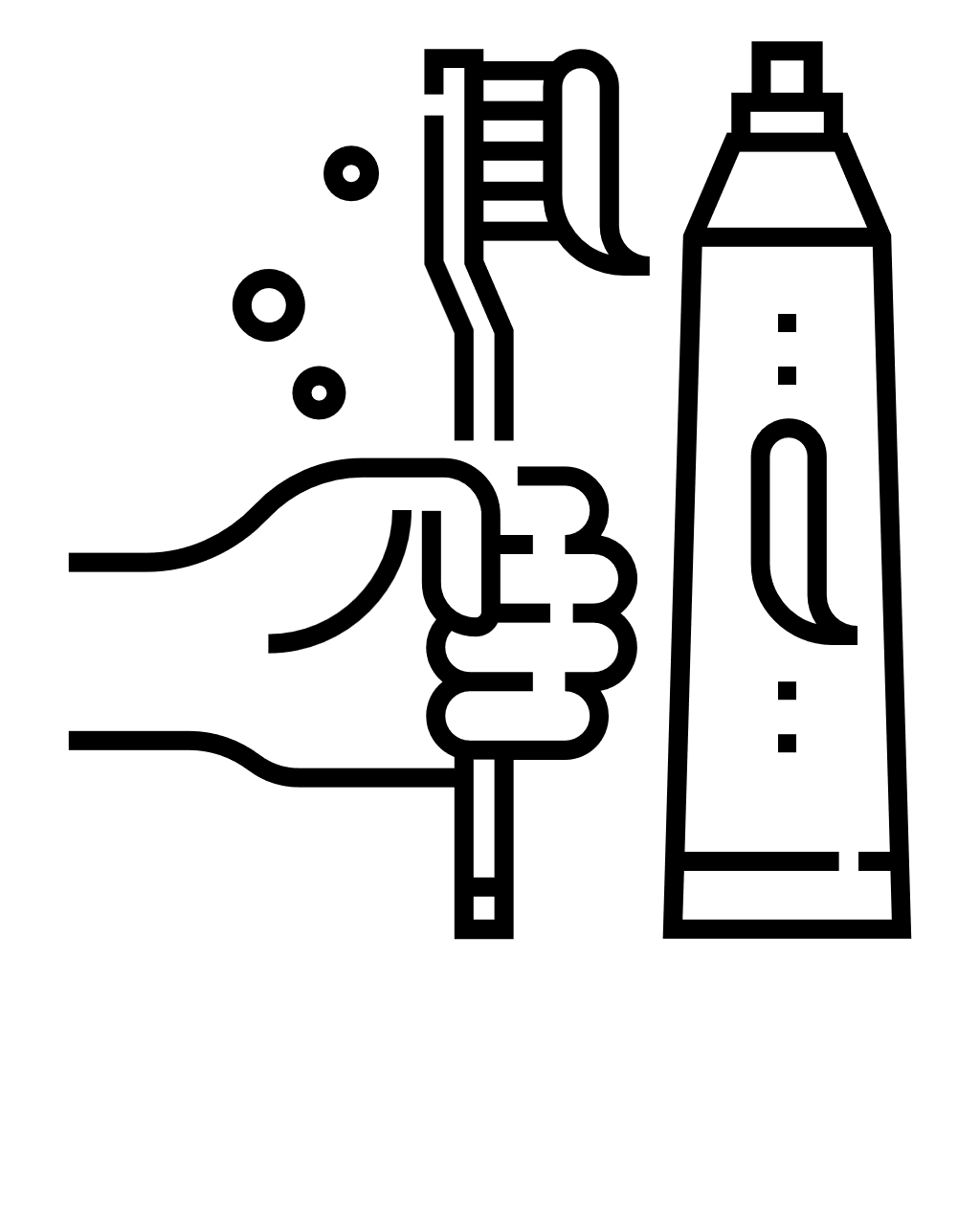 | 5 |
| 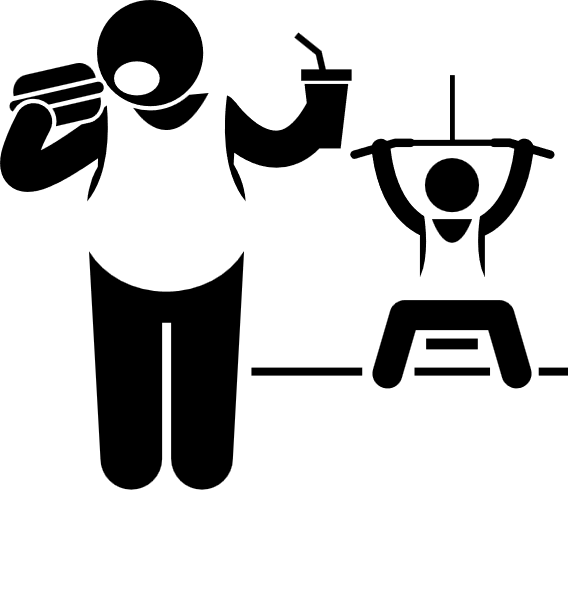 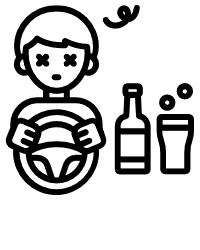 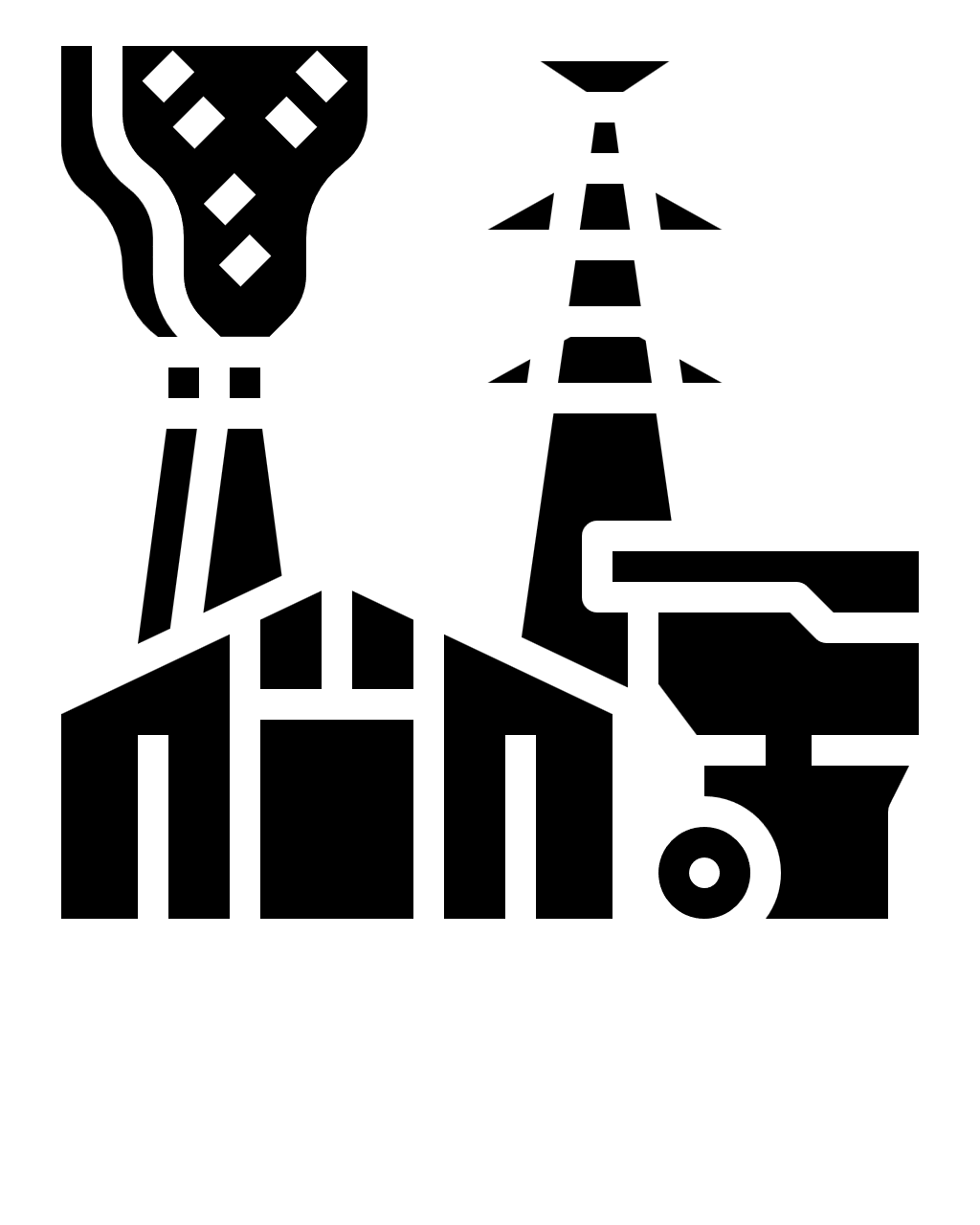 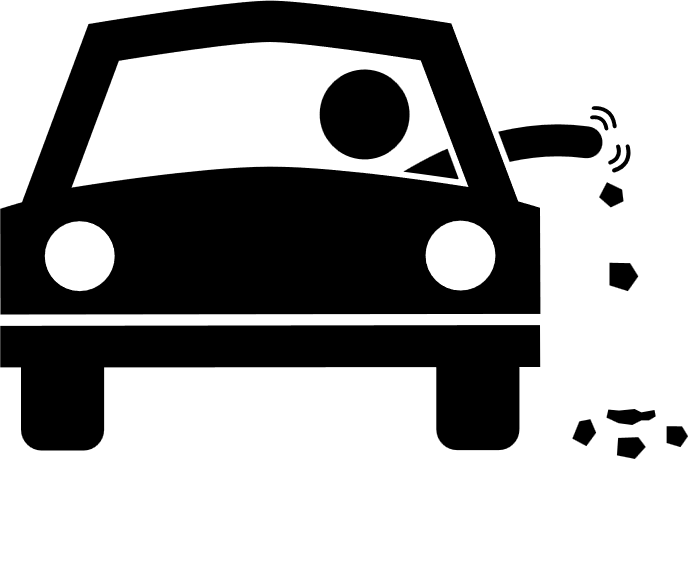 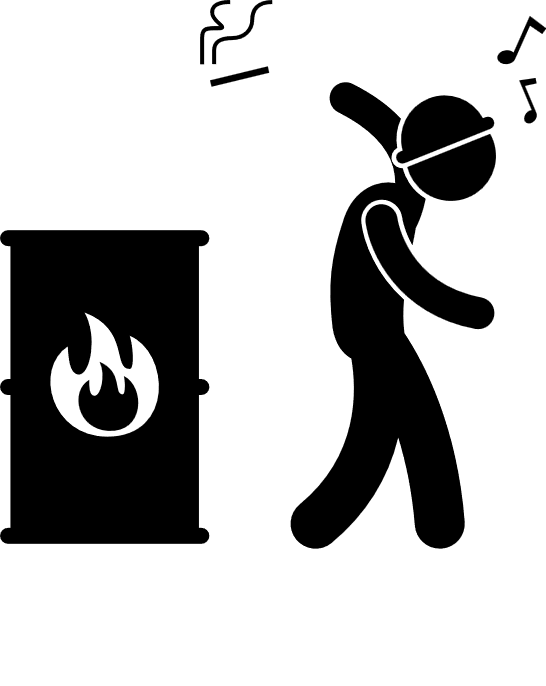 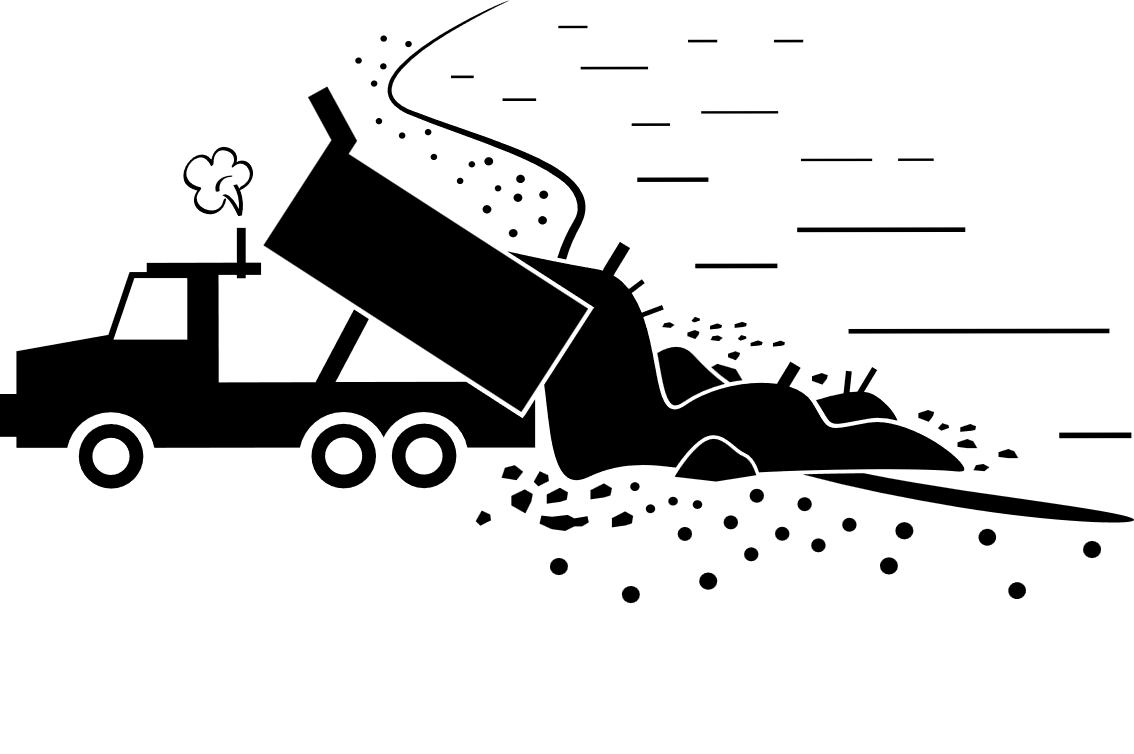 | 4 | 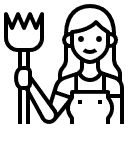 | 4 |
| 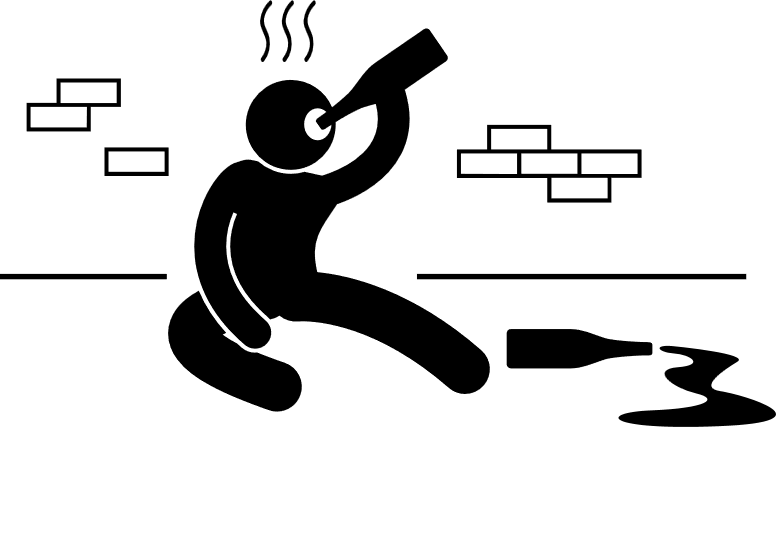 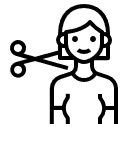 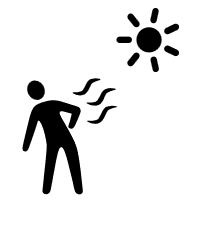 | 3 | 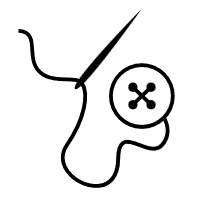 | 3 |
| 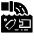 | 2 |  | 7 |
| 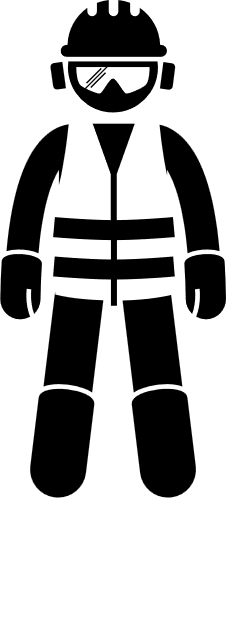 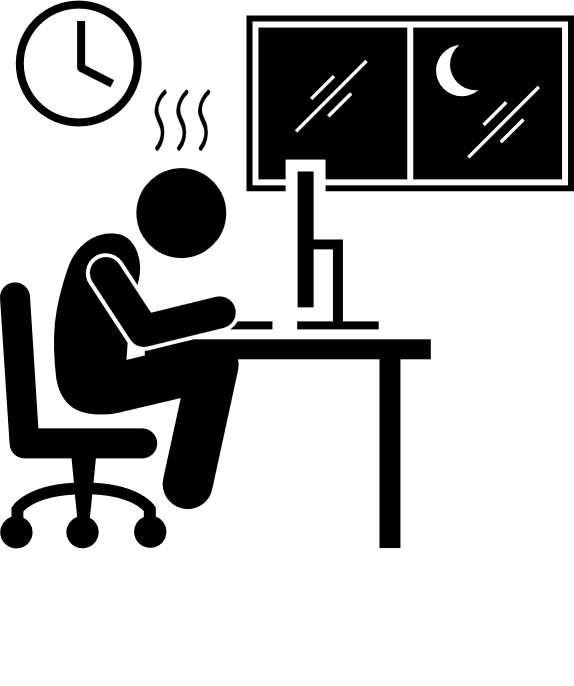 | 1 |  | 7 |

Grouping the icons into categories yielded the following.

|  | Future Reward | Immediate Reward | Total |
| --- | --- | --- | --- |
| Diet | 27 | 52 | 79 |
| Activity/Inactivity | 36 | 30 | 66 |
| Environment | 28 | 14 | 42 |
| Self-care | 24 | 17 | 41 |
| Financial | 10 | 23 | 33 |
| Gardening | 30 | 2 | 32 |
| Drinking/Abstinence | 7 | 25 | 32 |
| Housework | 12 | 10 | 22 |
| Health check-ups | 19 | 1 | 20 |
| Technology | 4 | 15 | 19 |
| Smoking/No smoking | 7 | 11 | 18 |

Only one of each icon was provided in the set given to each participant or pair of participants. However, when were combined, several of the icons had been sorted into future by some participants and immediate by others. Many of these were activities that have future benefit but can also be pleasurable in the moment. This is perhaps further increased where a behaviour is socially endorsed, thereby generating or enhancing a sensation of reward in the present moment. These icons are presented below.

| **Included in both Immediate Reward and Future Reward** |  | Future Reward | Immediate Reward | Ratio |
| --- | --- | --- | --- | --- |
| **Predominantly Future Reward** | 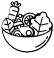 | 7 | 1 | 0.14 |
|  | 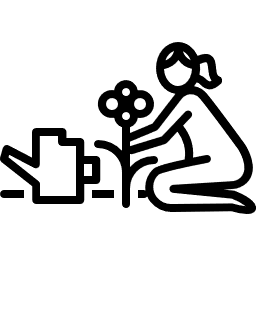 | 7 | 1 | 0.14 |
|  | 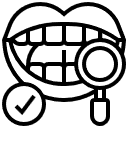 | 6 | 1 | 0.17 |
|  | 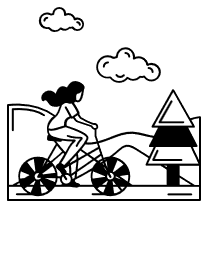 | 6 | 1 | 0.17 |
|  | 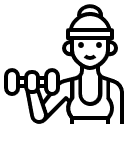 | 6 | 1 | 0.17 |
|  | 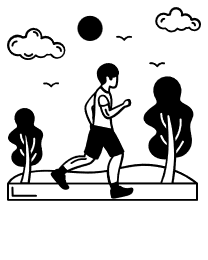 | 6 | 1 | 0.17 |
|  | 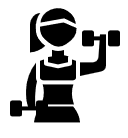 | 5 | 1 | 0.20 |
|  | 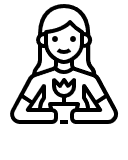 | 5 | 1 | 0.20 |
|  | 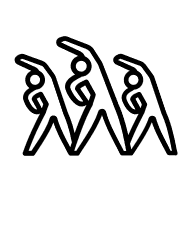 | 4 | 1 | 0.25 |
|  | 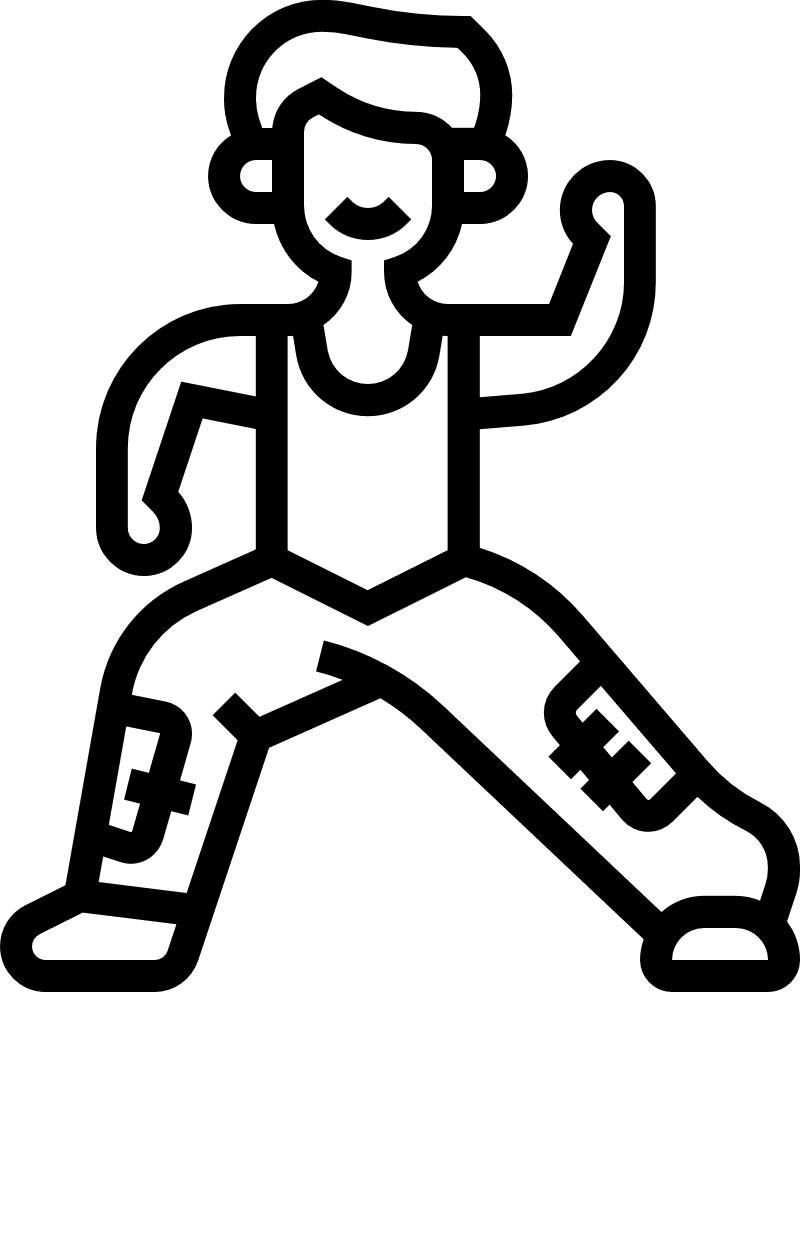 | 4 | 1 | 0.25 |
|  |  | 4 | 1 | 0.25 |
|  | 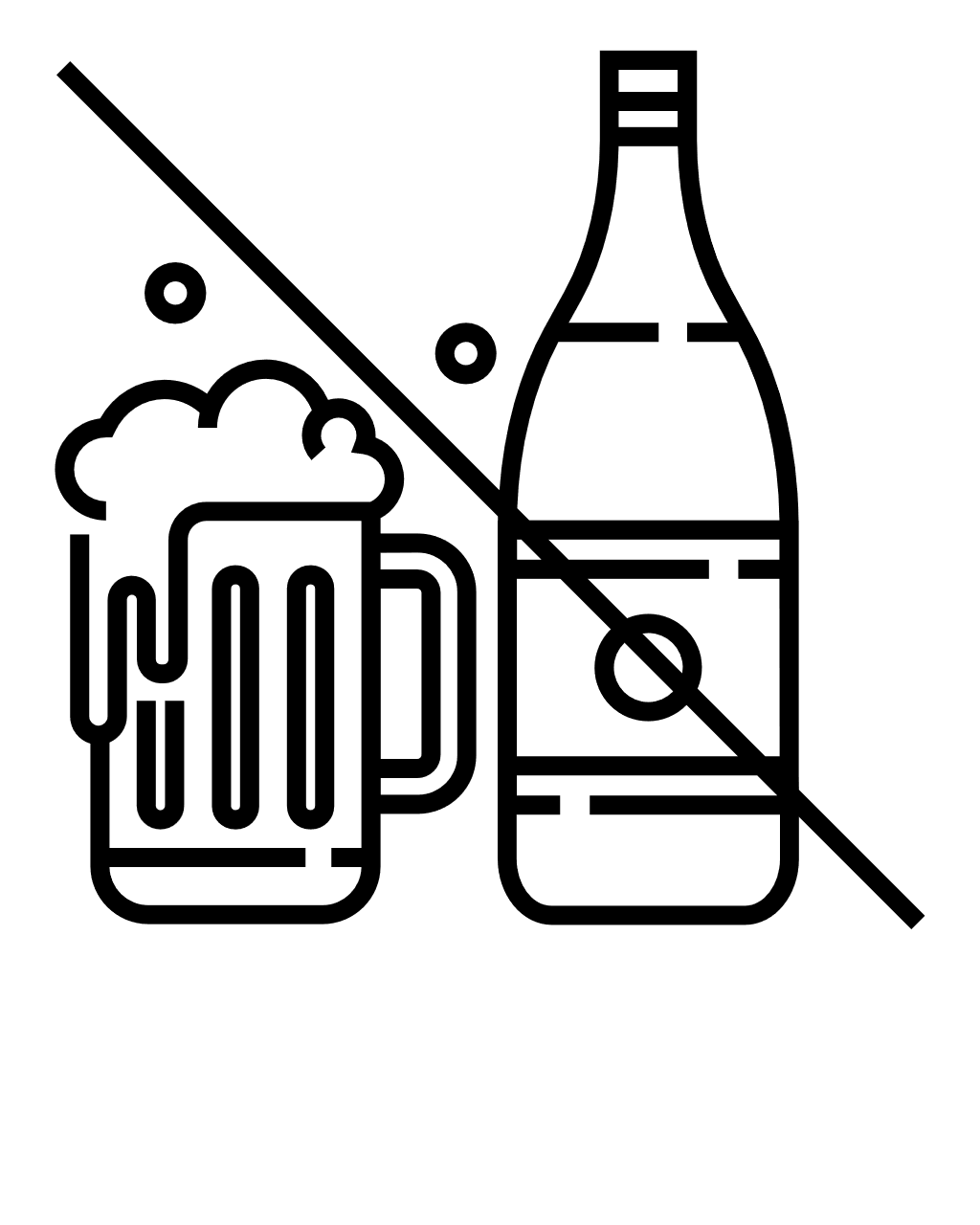 | 7 | 2 | 0.29 |
|  | 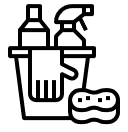 | 3 | 1 | 0.33 |
|  | 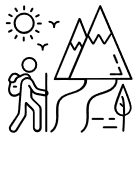 | 3 | 1 | 0.33 |
|  | 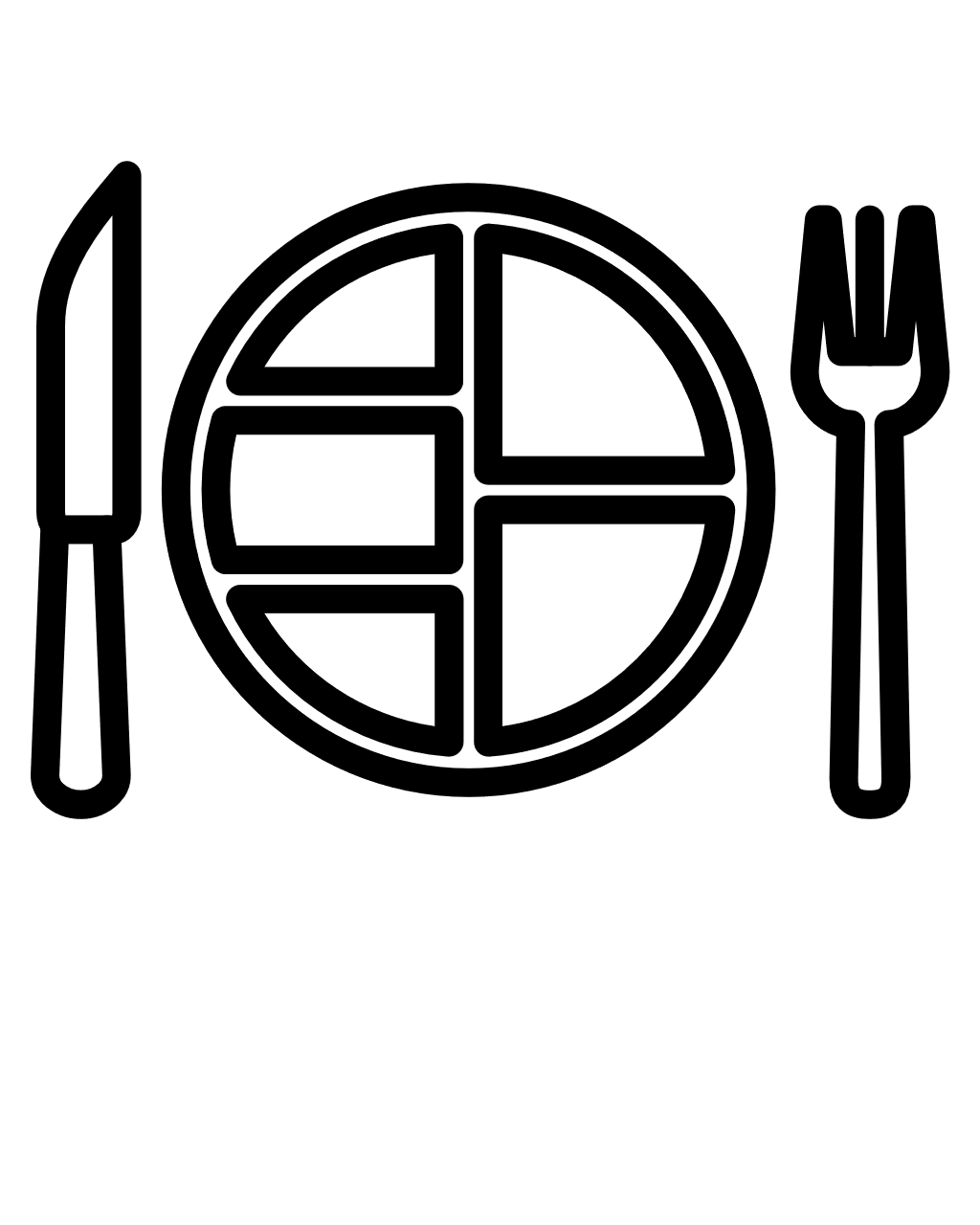 | 3 | 1 | 0.33 |
|  | 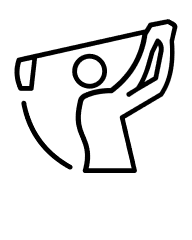 | 4 | 2 | 0.50 |
|  | 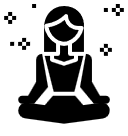 | 4 | 2 | 0.50 |
|  | 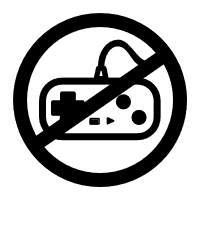 | 4 | 2 | 0.50 |
|  | 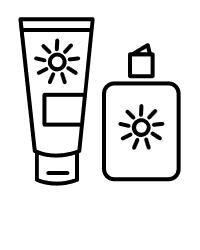 | 5 | 3 | 0.60 |
|  | 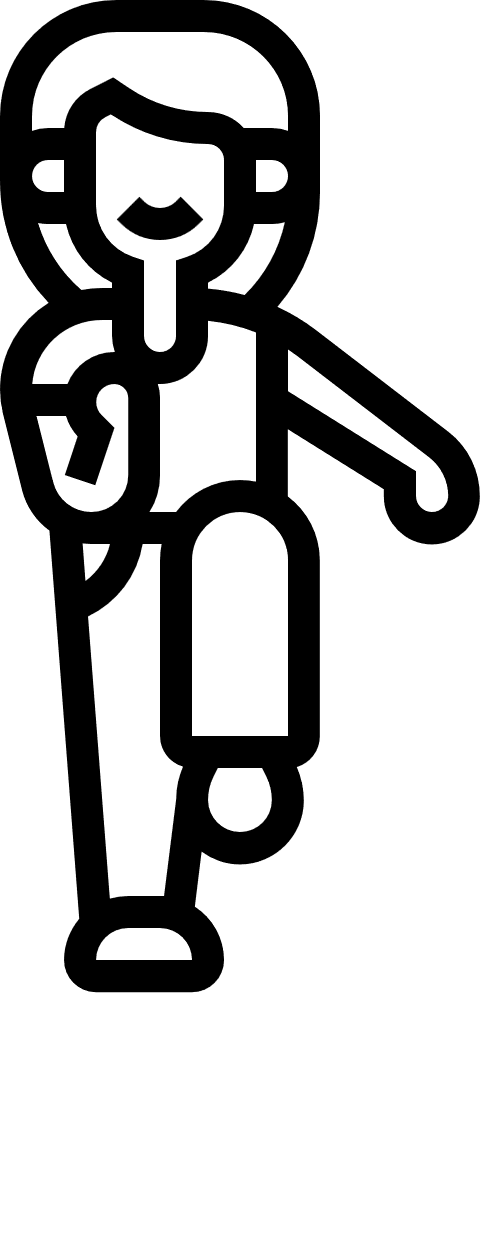 | 3 | 2 | 0.67 |
| **Immediate and Future Reward even** | 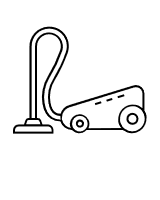 | 2 | 2 | 1.00 |
|  | 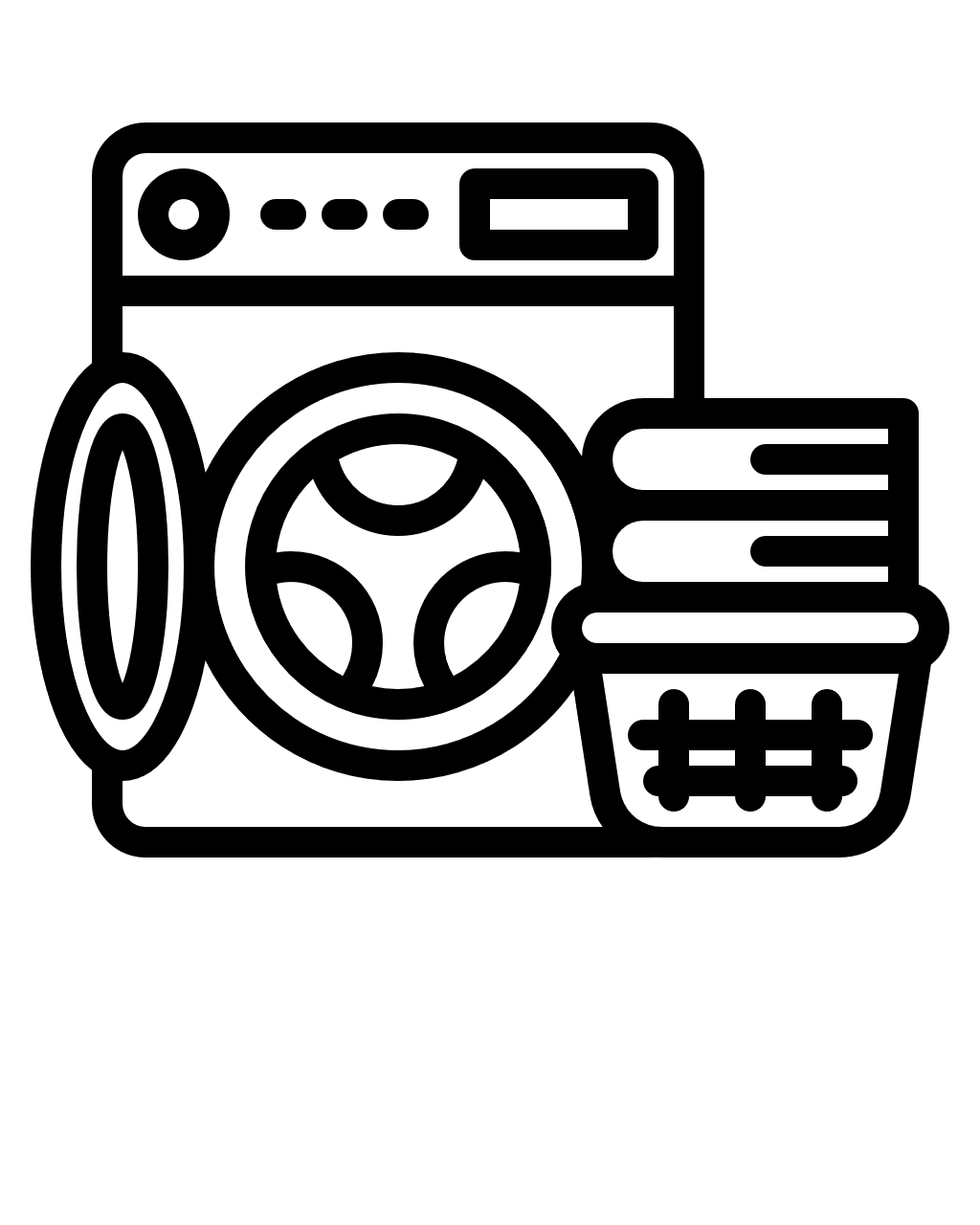 | 2 | 2 | 1.00 |
| **Predominantly Immediate Reward** | 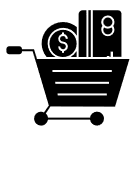 | 1 | 5 | 5.00 |
|  | 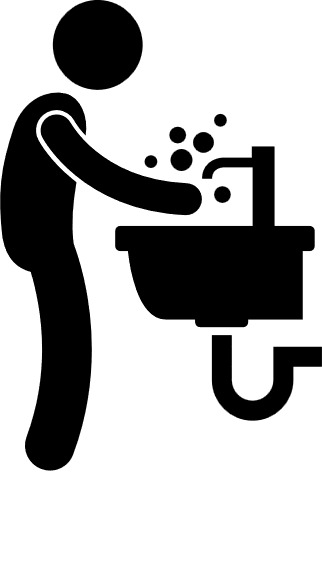 | 1 | 4 | 4.00 |
|  |  | 2 | 5 | 2.50 |
|  | 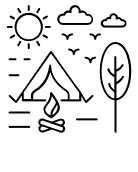 | 1 | 2 | 2.00 |

The same set of icons was also used in the Explain Future Discounting to a friend activity. Their use in this activity is as follows.

| Icons used in Explaining FD to a Friend Activity | | | | | |
| --- | --- | --- | --- | --- | --- |
|  | All workshops combined | Workshop1 (CBD) | Workshop2 (CBD) | Workshop3 (Christies Downs) | Workshop4 (CBD) |
| Activity/Inactivity | 29 | 11 | 3 | 5 | 10 |
| Diet | 19 | 8 | 3 | 1 | 7 |
| Garden | 15 | 2 | 1 | 4 | 8 |
| Self-care | 12 | 7 | 1 | 1 | 3 |
| Financial | 6 | 0 | 0 | 0 | 6 |
| Health check-ups | 3 | 1 | 0 | 1 | 1 |
| Drinking/Abstinence | 2 | 0 | 0 | 0 | 2 |
| Environment | 2 | 2 | 0 | 0 | 0 |
| Smoking | 1 | 0 | 0 | 0 | 1 |
| Technology | 1 | 0 | 0 | 0 | 1 |
| Housework | 0 | 0 | 0 | 0 | 0 |

## App changes feedback

| **General** | | |
| --- | --- | --- |
| Too many notifications | Can’t get past ‘sit tight’ | Scrolling screens out of order… when you scrolled down, you might see the last screen first instead of the first. |
| ‘Event 1’, ‘Event 2’…the events needed names. | I struggled with the pop-up navigation. I couldn’t get out. | Is the survey asking me about now or future me? |
| **Sliders** | | |
| It would be good to have maybe two more in between not at all/extremely. | It would be great if the graphic changed as you slide along, from less to more sleepy. | If it was like the survey with the smiley face. Then it would be a visual connection. |
| How fatigued are you feeling – slider seems opposed. Trips me up. How can I go back to correct? | There are two separate questions about sleepy and fatigued. Those I struggled with. Those are very overlappy. |  |
| **Goal setting** | | |
| Live composition of cue so you can see it as you enter it. | It would be good to see what your goals look like as a whole thing, as you’re putting them in there. | It would have been good to have some examples or ideas to make them different. |
| Some guidance around wellbeing, diet, exercise, or whatever it is, because they said they had to be different and I thought, these are quite similar. | I wonder if [icons] might be more intuitive for people and you might get less bias if they are attaching their own interpretations to just icons. | Icons are more representative of behaviour rather than the goal you are aiming for. |
| I found it was perhaps too open-ended in terms of setting the goals. |  |  |
| **Badges/Achievements** | | |
| **Behaviours to reward** | **Examples, comments** | |
| **Your own accomplishments** | ‘This week I ate an extra vegetable.’ | How many times have you done [something] towards your goal. Steps on a continuous path towards your goal. |
|  | ‘What is one thing you could do today towards your goal?’ with the option to prompt/track this over time. | Even, ‘I thought about my goal today’, or ‘I did [this] towards my goal’, could earn achievements. |
|  | ‘Not eating that pizza’ ‘ | I did [this] towards my goal…’ |
|  | ‘One positive thing I did today’ photos, shown in a gallery. | Checking off steps towards goals, E.g. I went to the gym three times this week, or I walked around the block. |
| **Completing EMA/viewing cues** | If you are planning on integrating the constant mood assessments, maybe you completed x percent of the mood assessments. | A badge for viewing cues, e.g. number of views per week. |
| **Future thinking**  **Reflect on process** | Think about the future/reflect on something you did today with the option to make a note. | ‘Do you have something to celebrate today?’ Even if it is just a reflective question. |
|  | ‘How are you feeling?’ | If it is specifically checking the goals that is potentially valuable, then you might want to reinforce that. |
| **Setting goals** | I don’t mind the idea of a badge for just setting your goals, early on in the process. | Setting 1st goal |
| **Social** | Social connections – if going social. For example, a badge for high fiving a friend. |  |
| **Rewards** | **Examples, comments** | |
|  | …with the tree, it would be nice if there was a flower. | A list would be enough. I like a checklist. |
|  | Badges | An avatar doing that activity |
|  | If you take photos of things you are doing, they could be shown as thumbnails on a timeline. | Photos, shown in a gallery. |
|  | Positive language [of the visualisations – e.g. you are spending a day at the beach]– is itself a reward. Maybe you don’t need [other] rewards. | I would love that to be a part of a gallery, to look back on and see what is progressing. |
|  | Show in a list, with all the badges you could achieve (but haven’t yet), greyed out. | Streaks (and ‘freezies’) |
| **What would it take to recommend the app to a friend?** | | |
| If it makes me think in a more positive way (and why) | Scientific evidence | If I see myself achieving my goals |
| Results | If it works for me. I would need to see it finished and use it and then feel like it is working for me. | At the very least it needs to be user friendly and engaging, not a burden to your everyday life. |
| Connecting with friends is a big one. Like duolingo. Encouraging and inspiring to see other people – including family/friends. | I like that functionality, where you can give someone a virtual high five. Celebrate the fact that they reached the 100 day streak or whatever. | I would rather see pictures than cartoon characters. It seems like some kids’ app. |
| I have to see the complete product, finished, use it for a bit and see that it is working for me. | Tracking. A way of showing in the app, this is where I started and this is where I got to, that would help with that recommendation. | In addition to the result, did I enjoy the process of using the app is important. |
| **Other suggestions** | | |
| Use generative AI to create cues or create a visualisation of your cue. | Could AI give you strategies for how to achieve your aim. | Maybe a daily tip, or something. |
| The initial goal setting could be fun. | Goal setting is so broad. It could use examples – or an ‘I’m unsure’ button. | Or half-finished examples or a sentence starter. |

## Appendix I: Workshop templates and resources

**WS1: Icebreaker nametags**


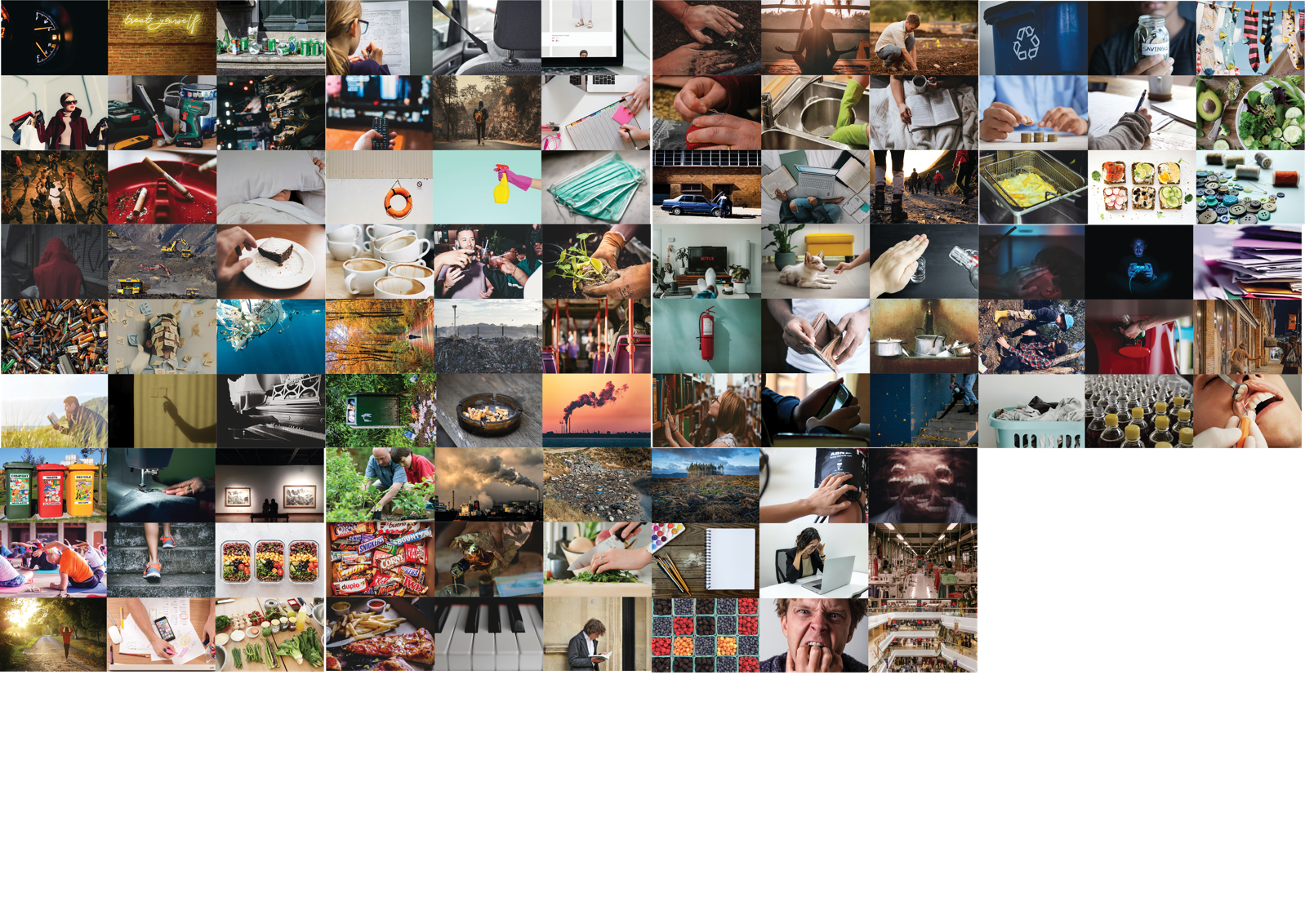


**WS1: Card sort**

**WS1: App Inventory**

**WS1: Job story template**

**WS1: Discussion prompt cards**

**WS2: Storyboard template and icon set**

**WS2: Icon sorting template**

**
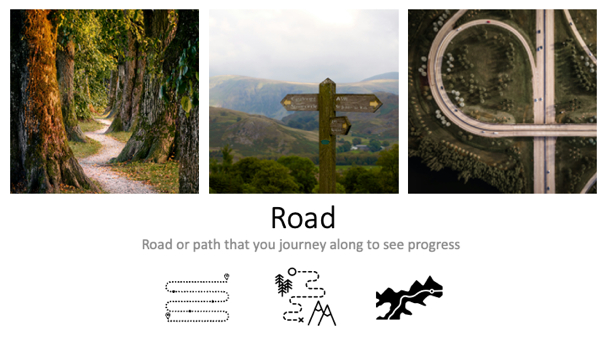

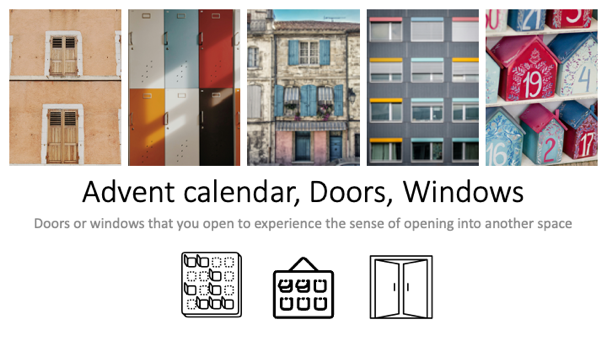

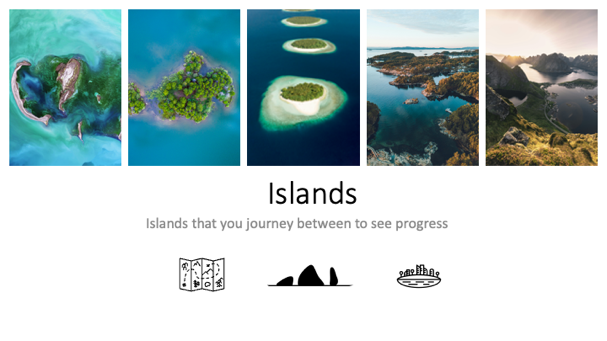

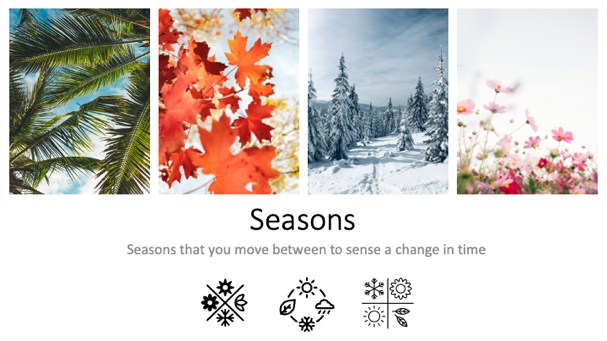

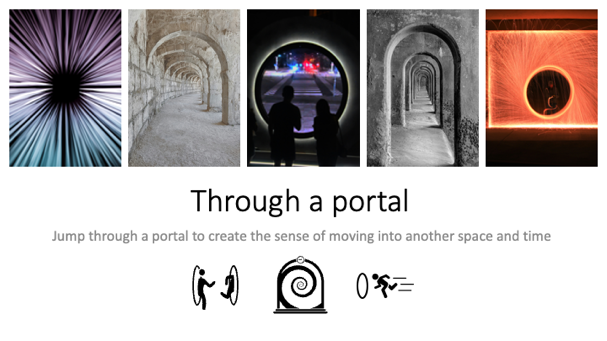

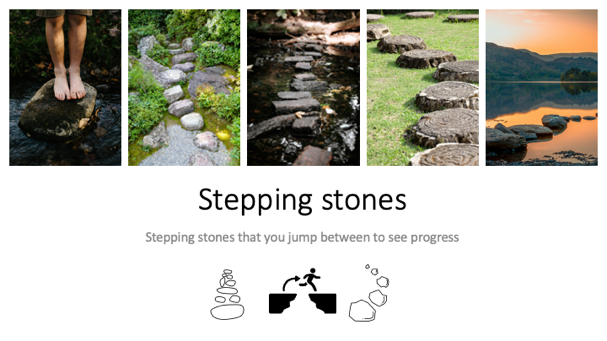

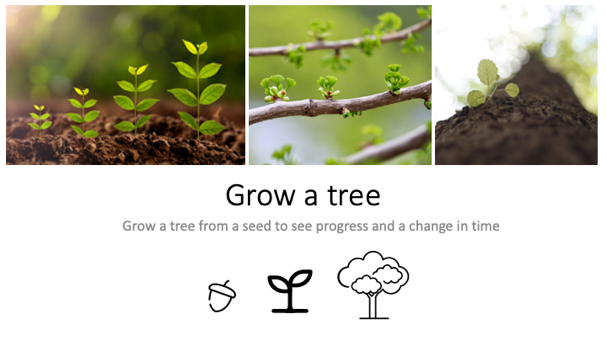

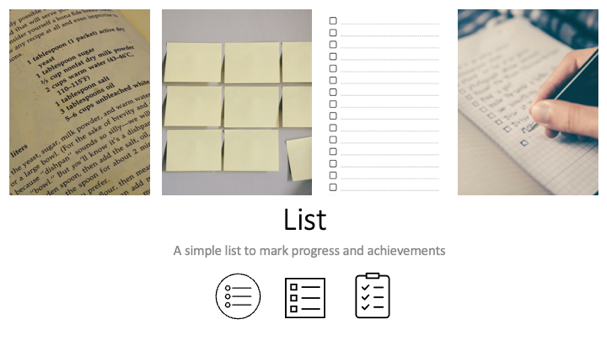

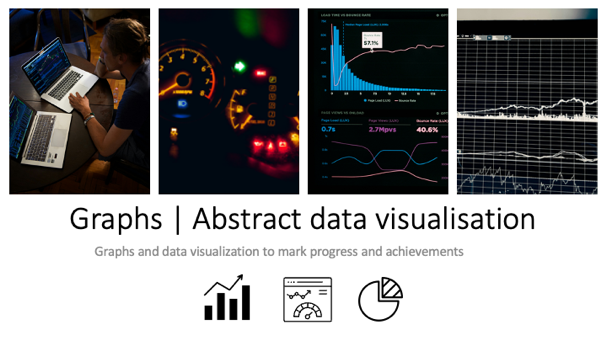

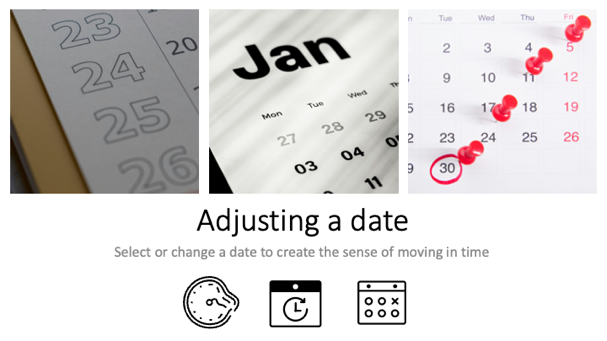
**

**
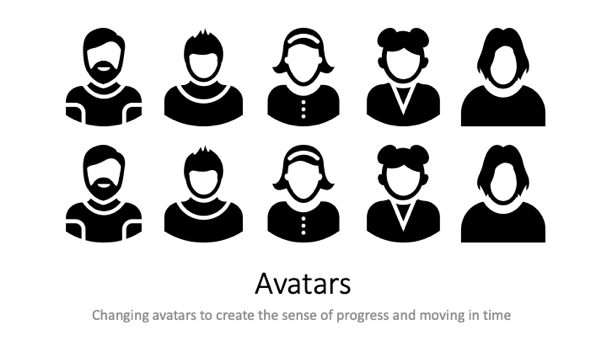
**

**WS2: Dashboard metaphors**

## Appendix II: Explaining Future Discounting to a Friend


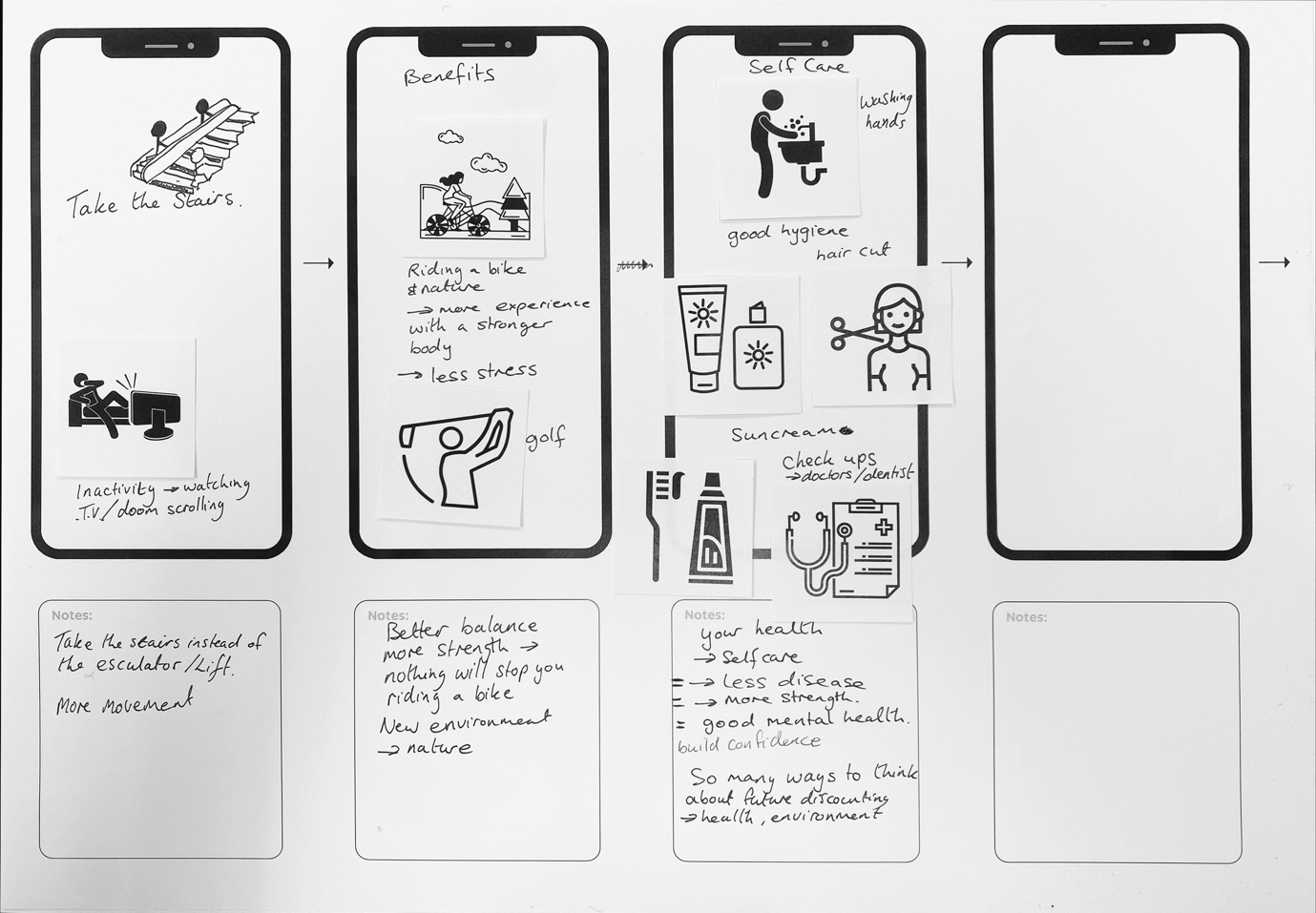


1.1.1

You don’t have to be at the gym, you can run for that bus.

Towards the benefits – you can go and ride a bike because you have the strength and the balance. You’ll have less stress and you can be in nature doing that.

More movement.

Then I did self-care – getting a haircut, good hygiene, looking after your teeth, going to have checkups. Leads to less disease, more strength, good mental health.


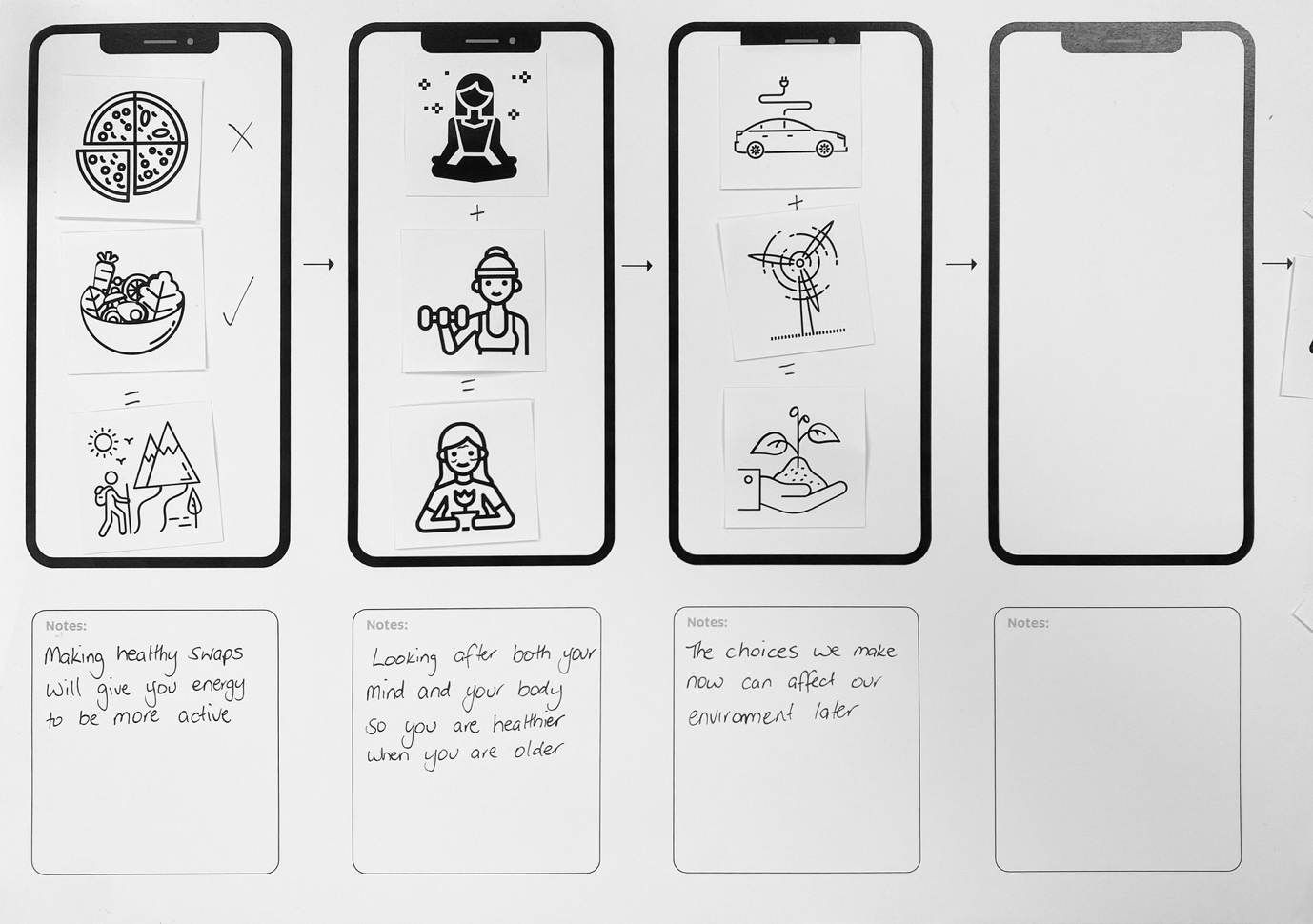


1.1.2

I’ve done similar but more specific - I’ve done a food one, I’ve done a physical one, I’ve done an environment one. The choices that we make with our food – so if we make some healthier swaps – we may have more energy to then go out and do what we like.

I heard the reason people are in aged care homes, the reason they struggle to get off the toilet, is because when they are younger they don’t move, they don’t keep themselves strong. So when they finally get to a certain age, they actually lose their muscle rather than retaining it and lose the ability to do everyday things.

So my thing here was the self care. Try not to stress because that also stops us from doing things. Keeping the body strong and that doesn’t necessarily mean going to the gym and lifting heavy weights, it can be going for a walk, etc.

I’ve put wrinkles on that person to show that when they are older they can the things that they want. People might not need to go into a home if they keep their body strong now.

And I did an environment one… the choices that we make fossil fuels, rubbish, things like that, will affect our environment later. Leaving legacies for children and grandchildren, we want them to have a good environment. Good choices now to enjoy the world later.


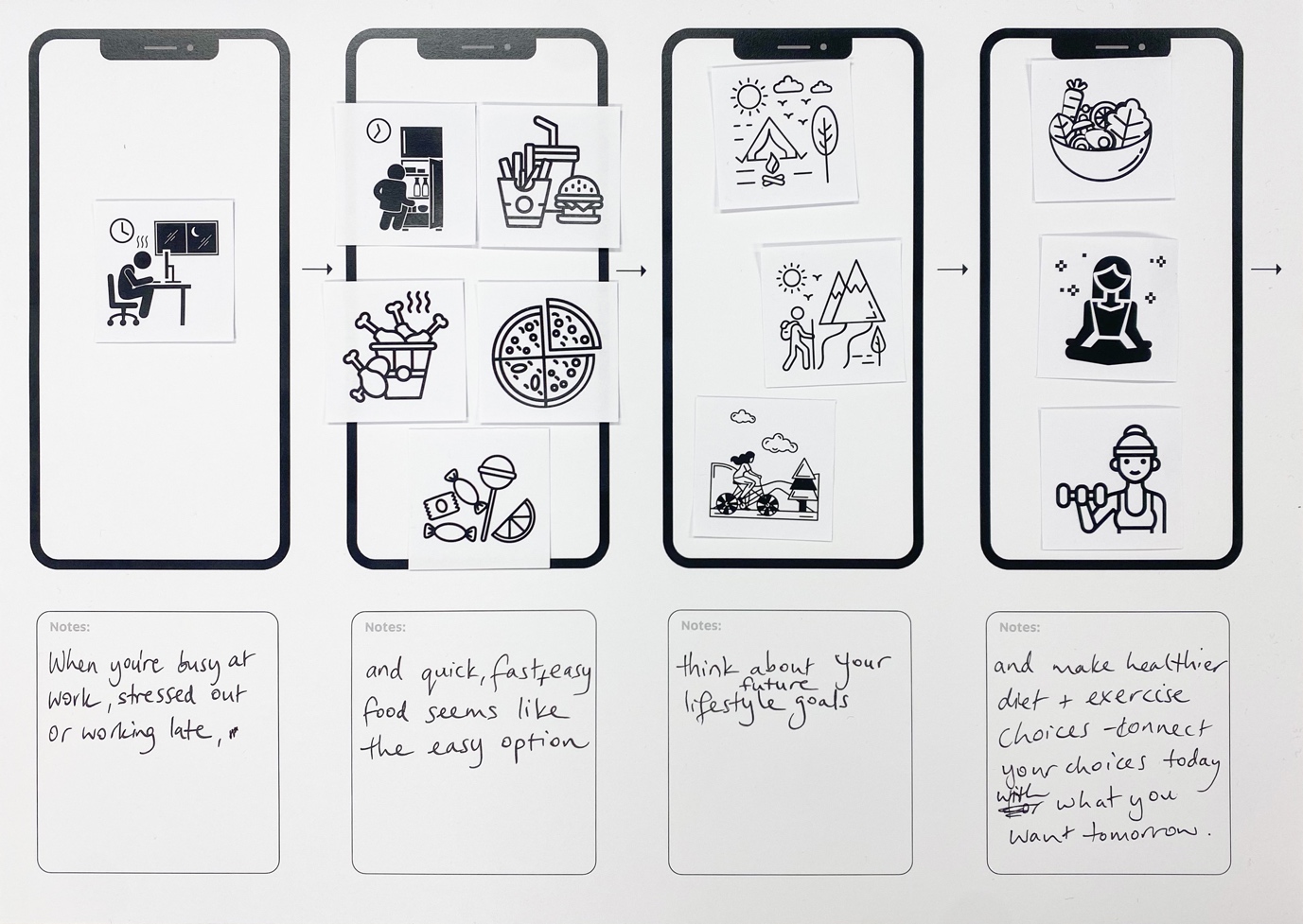


1.1.3

I picked a moment like when you’re really stressed at work, have had a busy day, working late. And the last thing you think about is getting something healthy. You just want something fast, easy. Probably not so healthy for you. The call to action is try to think about the lifestyle that you want. So going for walks, hiking, being out in nature, having a healthy body – and then make those healthier choices, to connect your choices today to what you want for tomorrow.


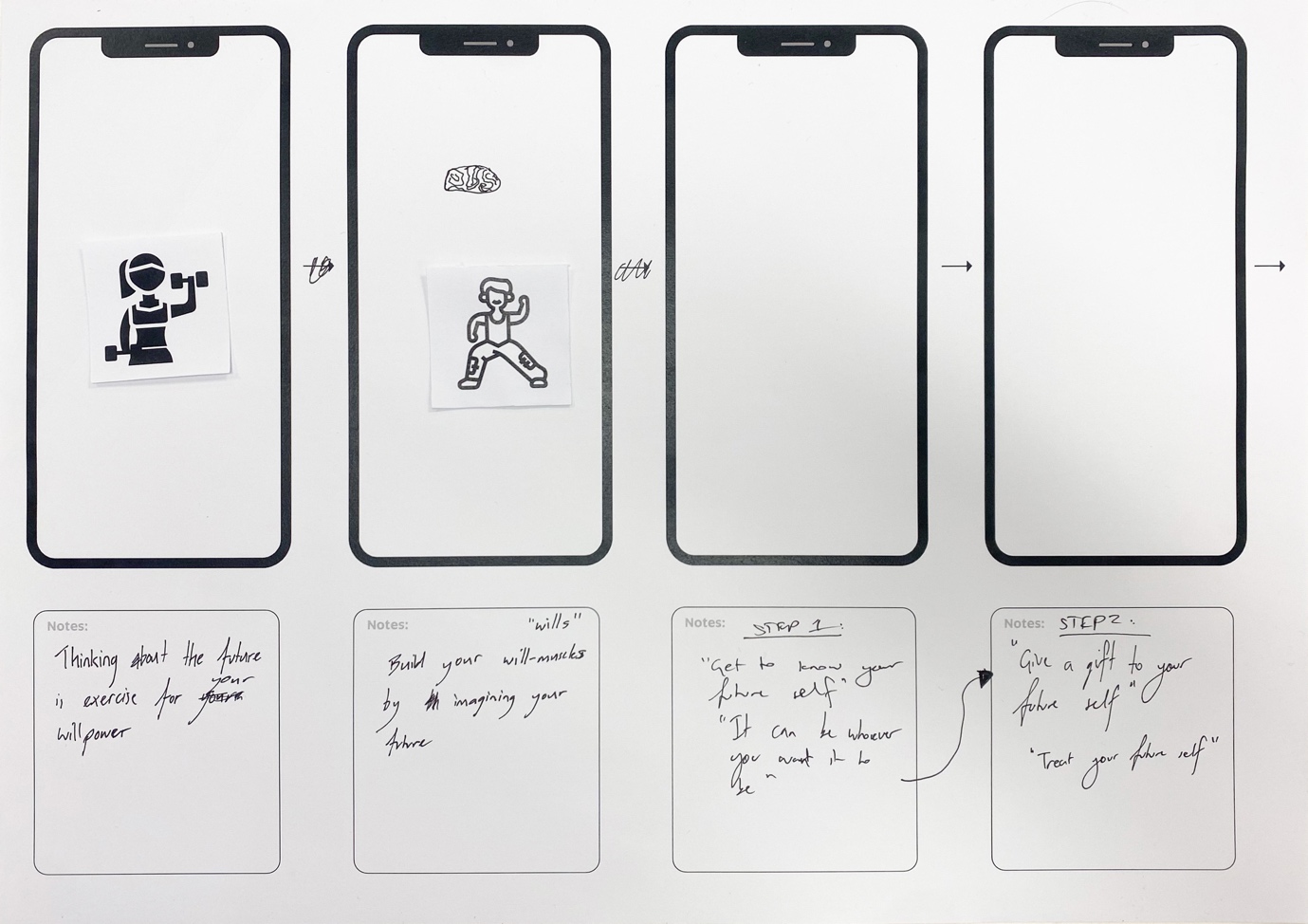


1.1.4

Thinking about the future is exercise for your willpower.

Build your will-muscles by imagining your future.

Change from ‘I have to…’ to ‘I get to…’ Remove the element of sacrifice. Add something in, not take away. A gift to your future self.

It’s more like building self-confidence with your choices – I am making this choice for myself. Doing it and seeing improvements, little improvements but it is something that you did. That’s confidence building and positive.

Get to know your future self. It can be whoever you want it to be and then once you get to know your future self, give a gift to your future self or treat your future self.


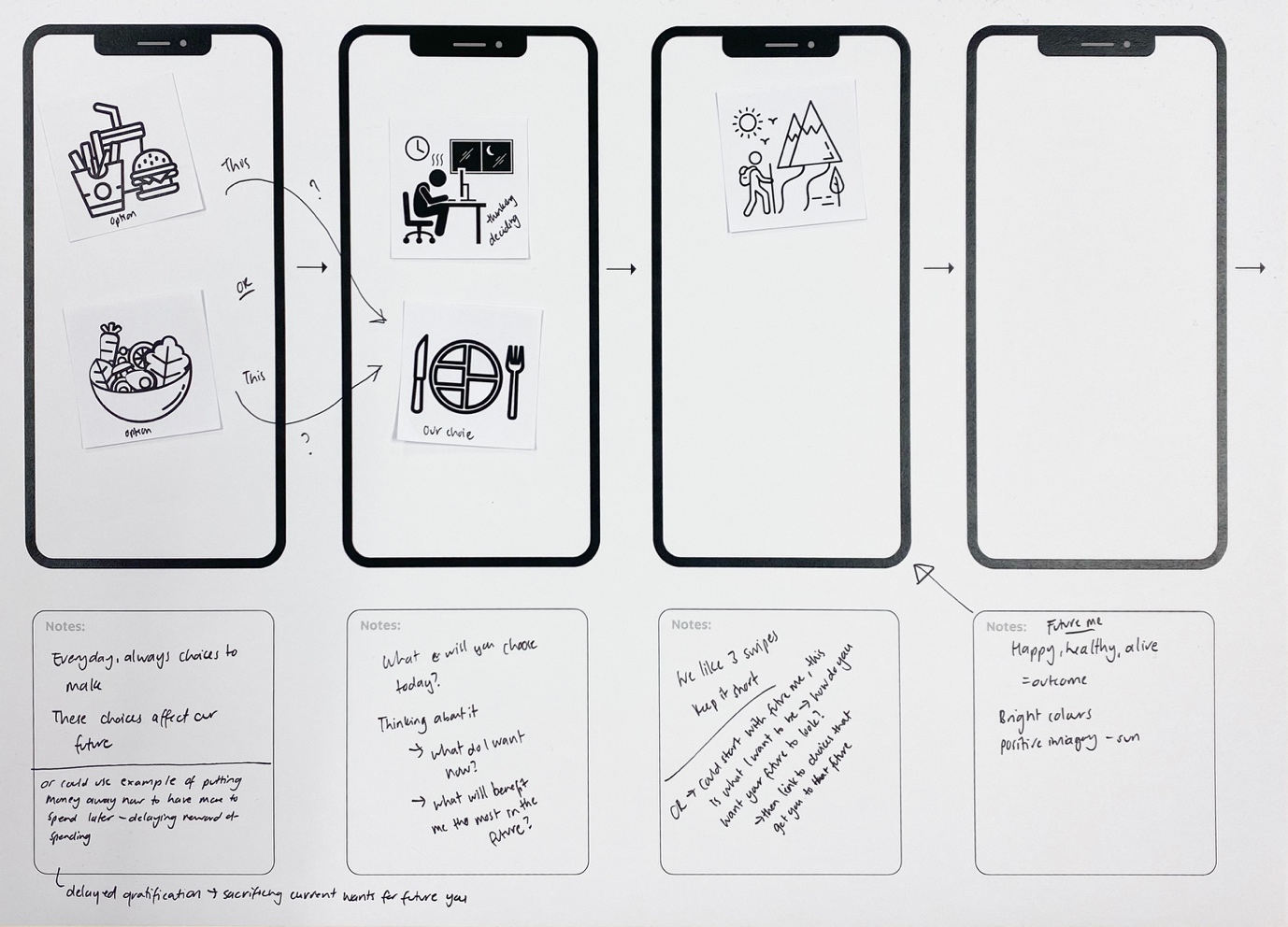


2.1.1

We first talked about how, reiterating that everyday there are choices that we have to make at the time can affect our future. And an example of that – we gave a food example – with fried food vs healthy food – and plate. You have to decide what goes on that plate. Depending on that choice there will be outcomes, down the track. One is happy, shiny, healthy person and the other is a less positive outcome. We also talked about the possibility of starting with the future me, the life you want, the choices you make now are going to influence your ability to get there and then kind of going back from there. And then we talked about a money example as well - about putting away money now so you have more in the future and then described future discounting as delayed gratification, so sacrificing current wants for future you.


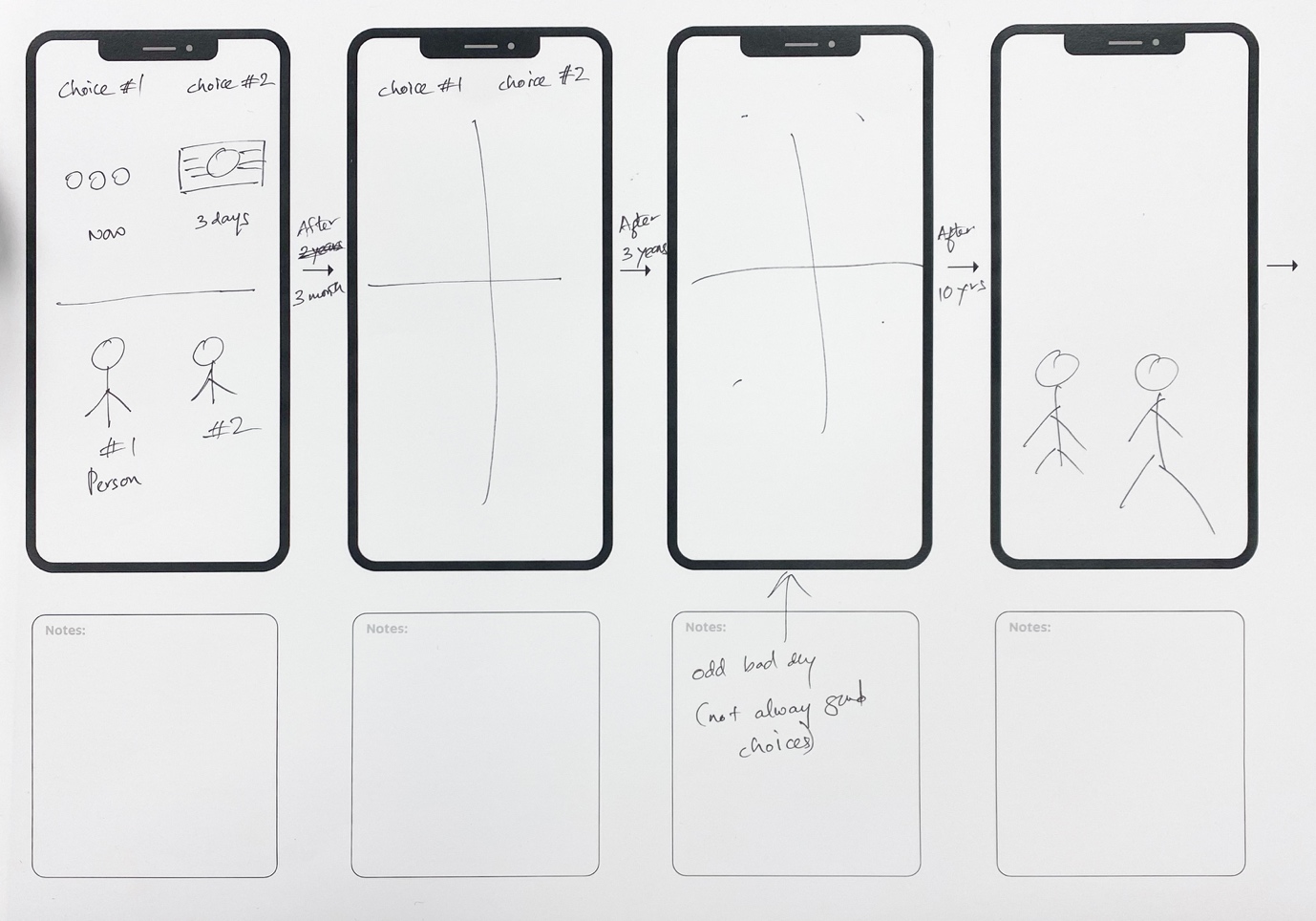


2.1.2

My idea was something similar to the slideshow that you showed. It’s a series of two choices. Just like, now and in 3 days. And there are 2 people, making those 2 choices. And they are gradually progressing – 3 months, 3 years, 10 years – and I want to show where they end up but at the same time, I want to show them doing the opposite choices, in the middle, so they are not only stuck to the good choices the whole lifetime and not bound to do only that. They can do other course. But that would need more screens. So would need to decide how many screens you need to give that a realistic approach.

So initially, the ‘good’ choice might not have any instant benefit, that can also be shown here. I am enjoying my food but I am not getting anything.

Like those super fund ads that have the comparison between two people over time and show how much money has accumulated.


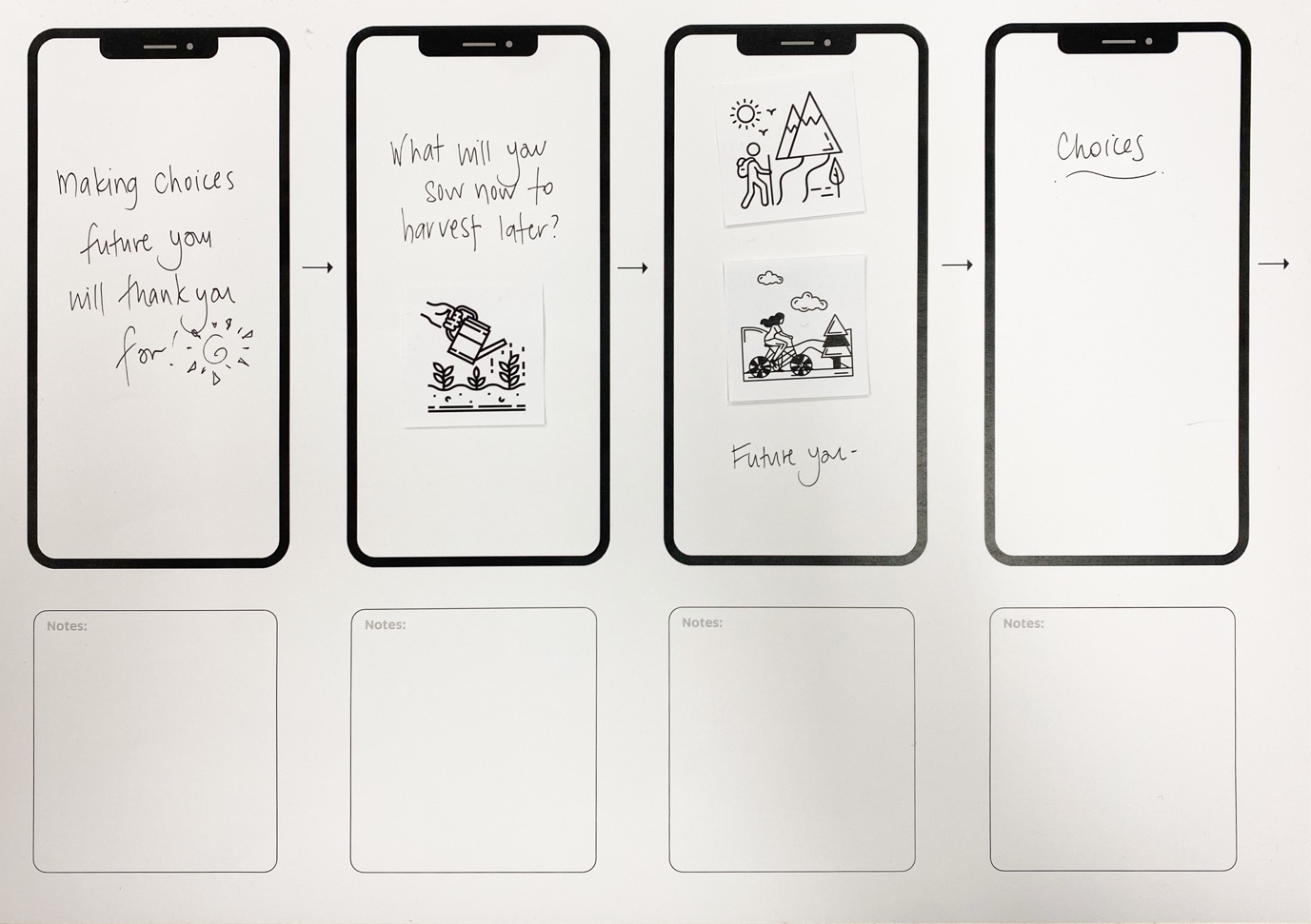


2.1.3

I didn’t really have a good story. I was thinking, I guess, more about tag lines.

*“Making choices future you will thank you for!”*

Less about deprivation, more about doing things that you know you can benefit from. Positive.

*“What will you sow now to harvest later?”*

What are you investing yourself – your time, your money and your health in – your energies in – for later use.


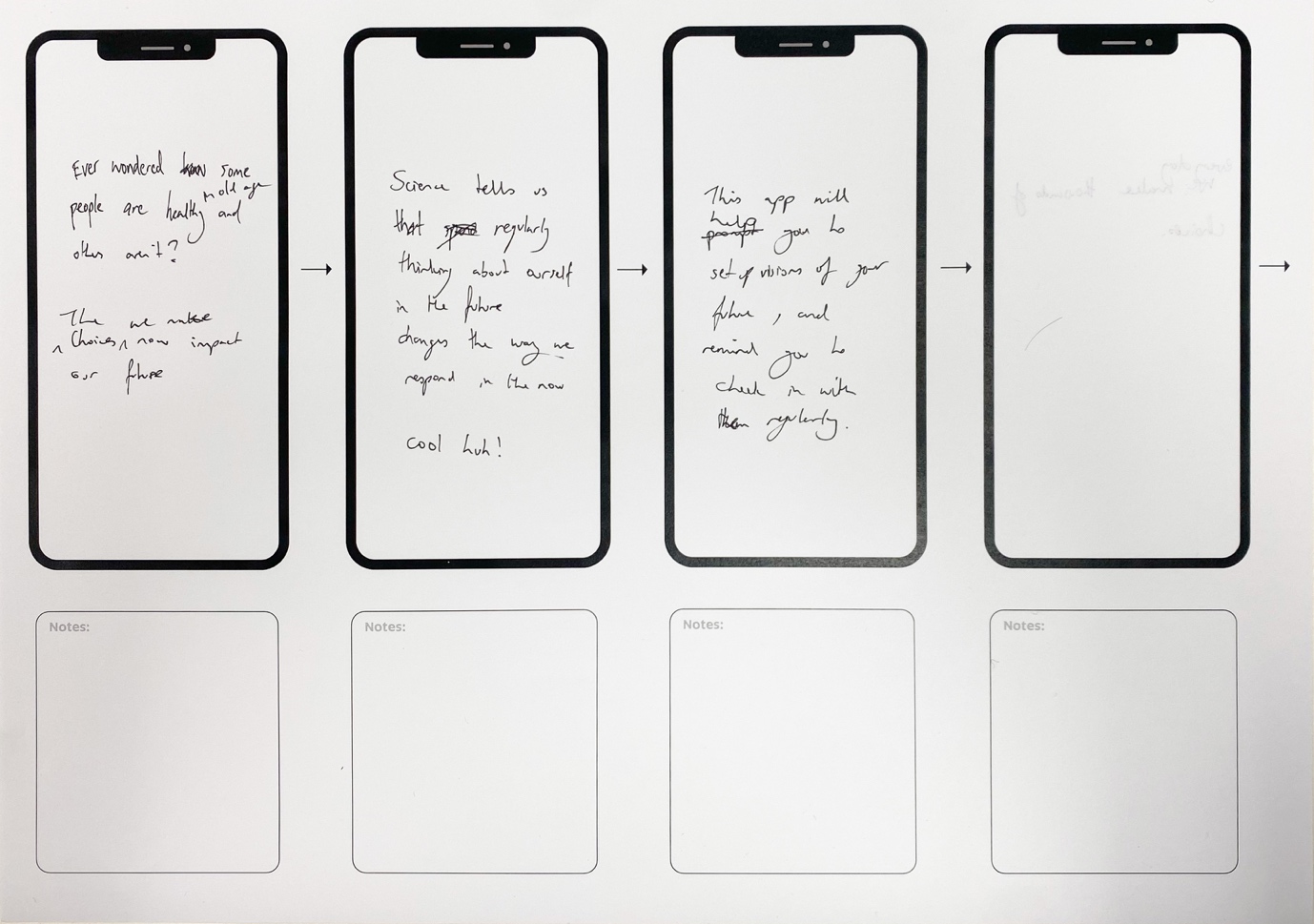


2.1.4

Ever wondered how some people are healthy in old age and others aren’t? The choices we make now impact our future.

Science tells us that regularly thinking about ourself in the future changes the way we respond in the now. Cool, huh!

This app will help you setup visions of the future and remind you to check in with them regularly.


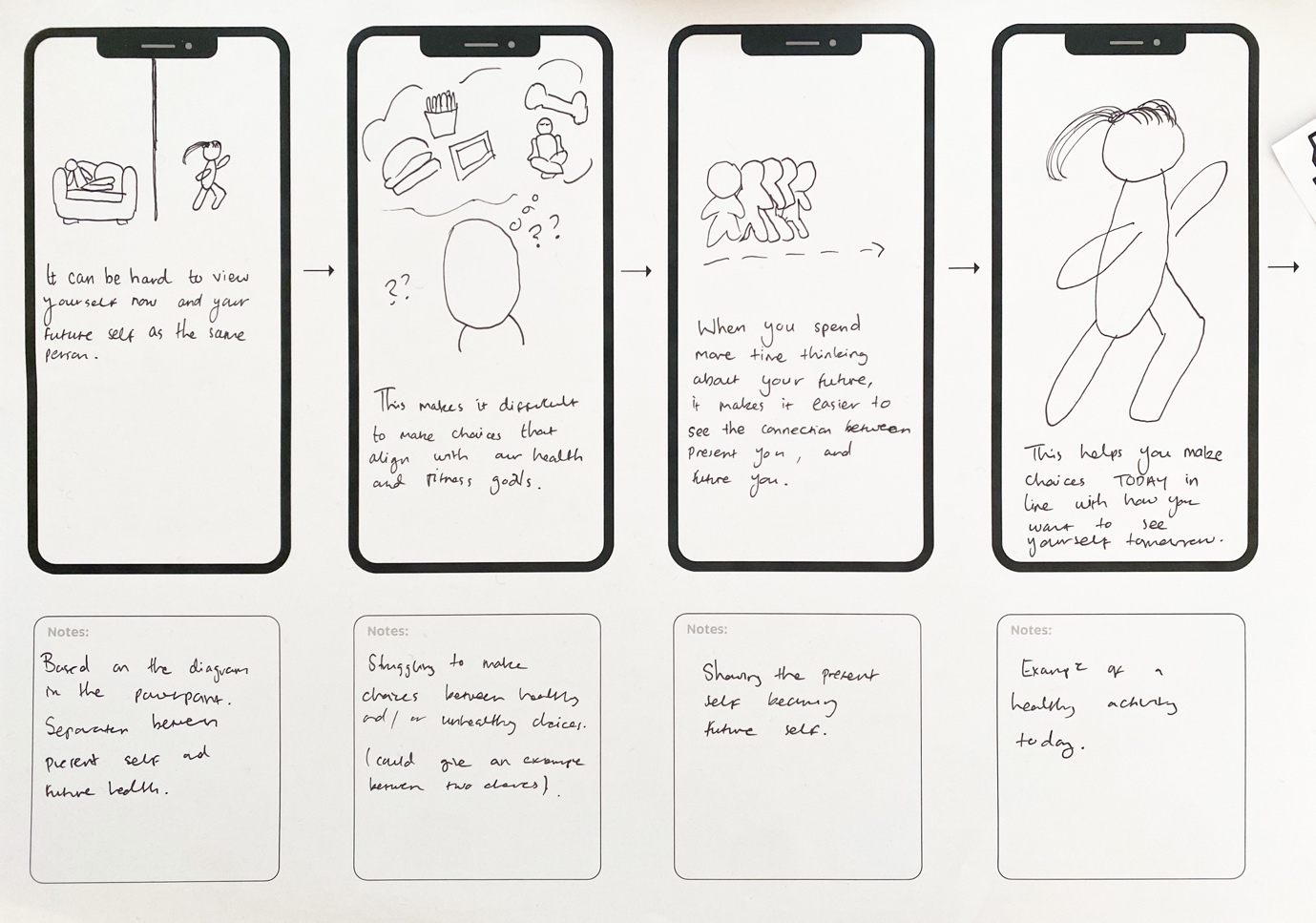


3.1.1

The thing that stuck with me from the previous workshop was that little diagram of the present self and the future self being separate and the picture underneath of them all being along the same continuum. So I based mine on that.

*‘It can be hard to view yourself now, and your future self as the same person.’*

Why does that matter?

*‘This makes it difficult to make choices that align with your health and fitness goals.’*

Decide…do I get McDonalds do I go for a run?

Then I used the same image of the bodies all being in a line so..

*‘When you spend more time thinking about your future, it makes it easier to see the connection between present you, and future you.’*

So, what does that mean?

*‘This helps you make choices TODAY in line with how you want to see yourself tomorrow.’*

And it’s got the person from today actually going out for a run because they’ve been thinking about their future and they’ve made the decision to go out and actually have a run today, which is sort of their future self that they felt disconnected from before.


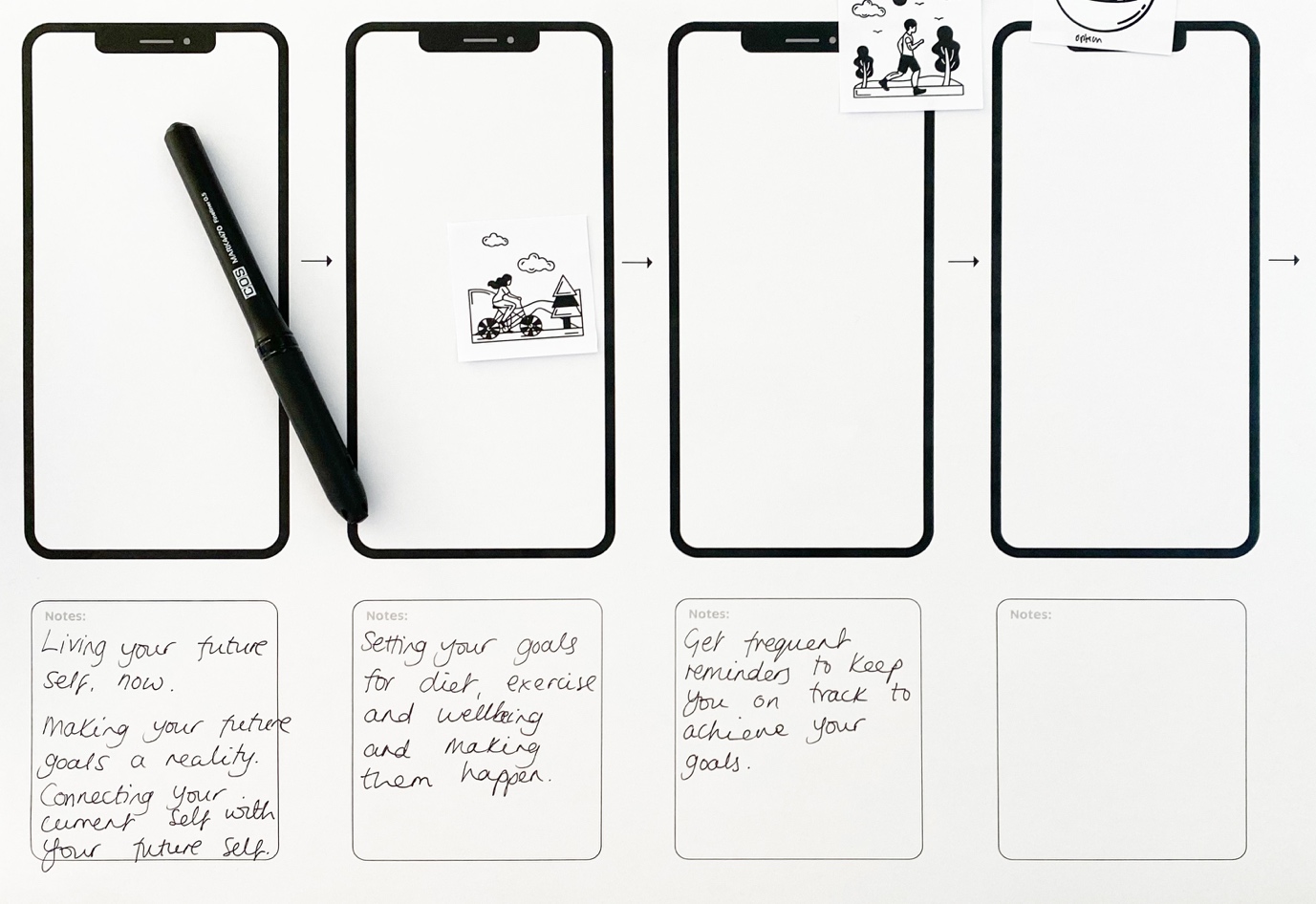


3.1.2

*‘Living your future self now. Making your future goals a reality. Connecting your current self with your future self.’*

And then moving on making it a little bit more explicit about…

*‘Setting your goals for diet, exercise, well-being…’*

I included ‘well-being’ because I like that.

*‘…and making them happen.’*

So not just as the abstract, separate goals. And then…

*‘Get frequent reminders to keep you on track to achieve your goals.’*

(Would it help to have well-being as a third category?)

I would see it as sitting above them though. What you achieve through diet and exercise, has an overall impact on well-being. There are other practices I would like to achieve, like mindfulness, that I wouldn’t necessarily categorise as exercise or diet. Also, things like sleep hygiene.

It does feel very binary on the app.


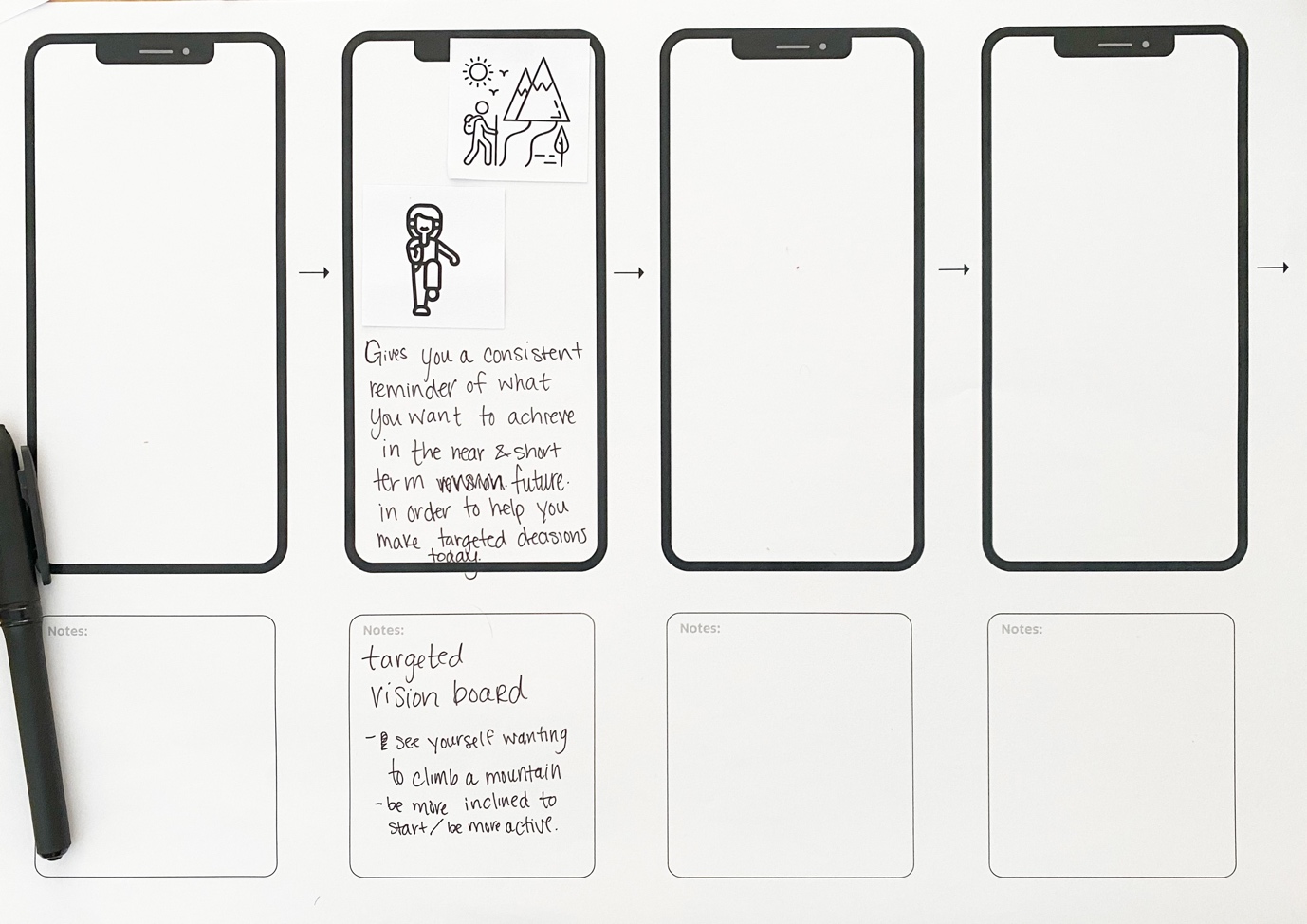


3.1.3

I saw it as a targeted vision board – things you see yourself having, or wanting for the future – but here is a little more specific, a little more now.

*‘Gives you a consistent reminder of what you want to achieve in the near and short term future in order to make targeted decisions today.’*

I think it is great to have it as a vision. I think it would be great, on the app, to have a thing to be able to send yourself a pdf to print, and you could stick up those future cues - like on your fridge, or by where you study - so you can use the app to do it but also if you’re not picking up your phone you can still look and go, yeah, I’m imagining. That could be useful too.

A print button.


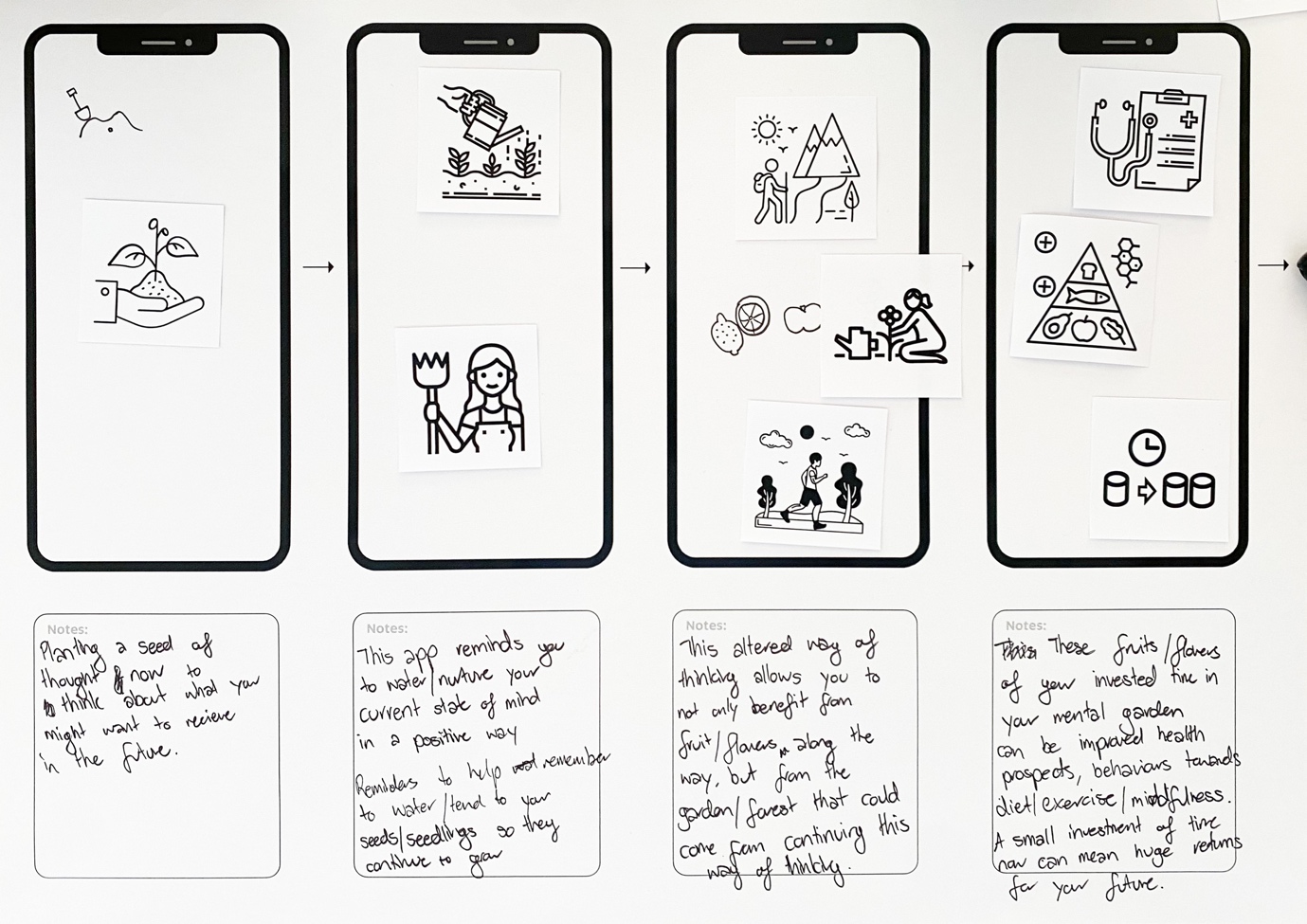


3.1.4

I connected back to what we talked about before we started, planting a seed…

…planting seeds, writing out your cues, making a plan.

Then the app reminds you to water, reminds you to look after, those seeds grow, to help you to get to the end goal of having the fruit or the flowers or walking through a forest at the end…

… and then tying that back to that forest is your health. Your diet, your exercise, your long-term enjoyment in the future.

(Planting seeds for your future, then helping to care, nurture)

And you can enjoy the fruit along the way too, before you get to the big [forest]. It’s not a long-term, never-going-to-happen thing.

(A garden is never finished)

Isn’t that what it’s like though? It’s not as if you get to retirement and that’s it, done.


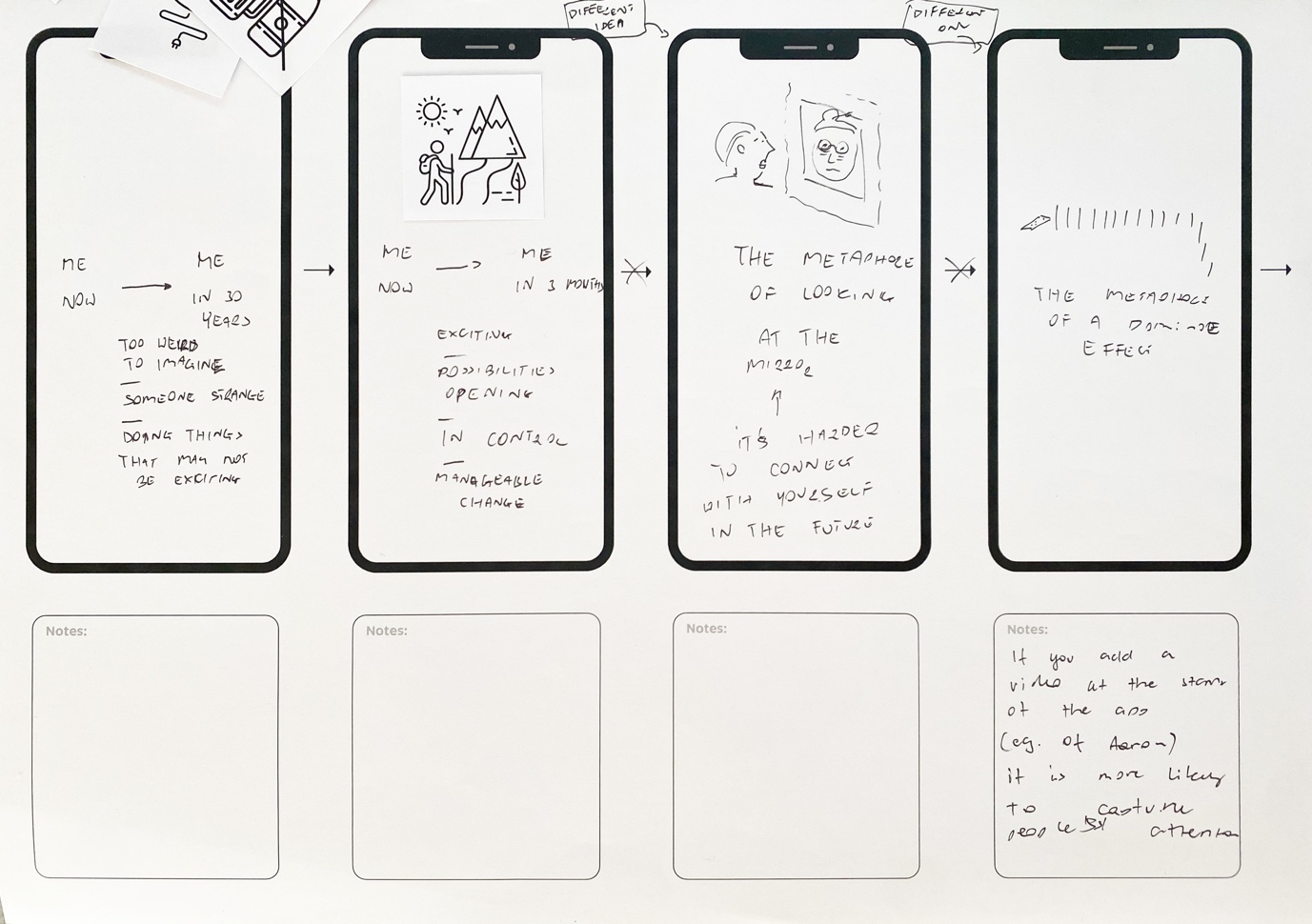


3.1.5

I was trying to put something that would look good in the app and my thoughts were going in completely different directions…

When I think about exercise, I think, no matter what I do, the person 30 years from now will be kind of weaker, it’s not going to be strong person that is running marathons. It’s unattractive, I don’t want to really think about it. Unpleasant future. When I think about myself in three months or a year, I feel like this is going up. I can do something, the possibilities are open because I feel like it is going to go up.

When I think about financial future it is easier to think 30 years from now, it is exciting. It might be really cool. But with the health and physical it feels the other way. So that was difficult for me to show it in the app because I was thinking looking at yourself in the mirror and you see old person in the mirror, that’s not nice, that’s not something that you want to do. You don’t want to do that exercise in the app. For me, the best part of the app was that initial setup, when you set your goal and makes you really imagine it. I really enjoyed it. But I wouldn’t enjoy it if I was setting it for 30 years from now.

Imagining that small manageable thing that you can do today.

It’s not about you being completely different, it’s about every single choice being an opportunity.


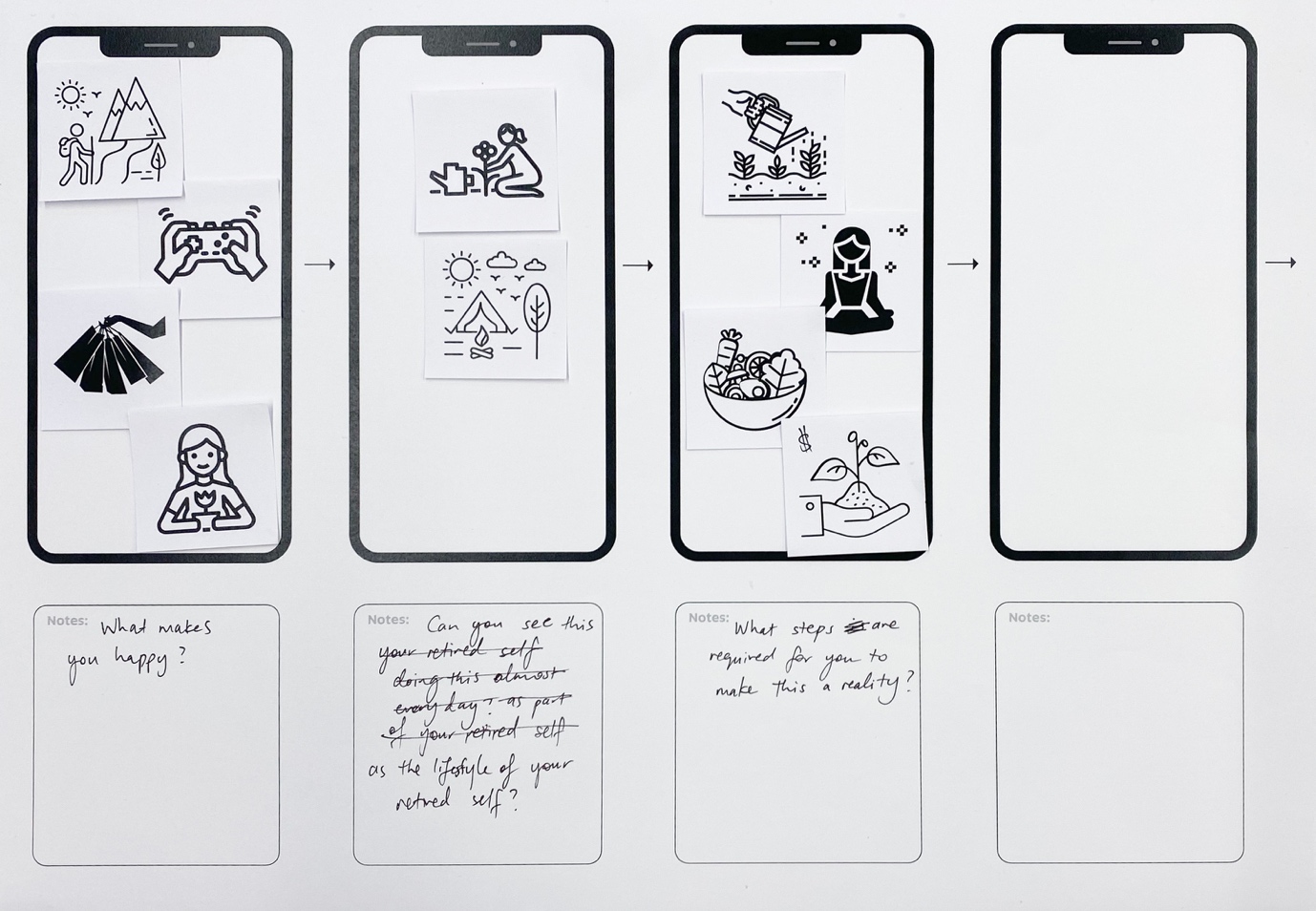


4.1.1

What am I doing now that I enjoy that I would like to keep doing? A little bit less of that to get more of this.

It’s easier to continue doing what you’re doing and then just cutting back on that.

The more healthier choices you make, the easier it is. And then you start making habits. I found this was really timely – I have just joined a gym challenge. I’ve never done one before and I thought it was all based on weight. They asked, what are your goals? Just to be here 4 times a week, basically. But then they had a really good app, and it had really good recipes and I thought, well, if I am going to do this, I’ll see what I can do, cut back on and do that. So, going 4 times a week meant going to the really early sessions. I am not a morning person at all. But I found going to that, the hardest part was just to get up. Once I was up, I just went and did it. Now, I have changed all my lessons to the morning ones because it is just habit now. It took 4 weeks to do it but I just want to keep doing that.

Small changes, but then it didn’t take long to become habit.

(Was that reinforced because you saw results from what you were doing?)

I just started feeling really energised. And my husband was like, ‘You smiled at me the other day. This is new!’ I have become a morning person.

So it was timely, I was entering into the app - how energetic do you feel? – well, I’m actually increasing there. It is making me want to do less drinking during the week and when I go out I think, oh, I have to get up tomorrow, I won’t have this next drink.

(It sounds like you’ve overcome future discounting by visualising tomorrow.)

(Even if you begin to see yourself as someone booking those classes that you’re going to attend in the future, so I wonder what this looks like if you just ditch that entirely and just look at that this app can help you think about the future as a way of keeping on doing what you are already doing and just letting those other health habits pile on naturally. Because it will make you not want to eat that junk food because you’ll know how it makes you feel.)


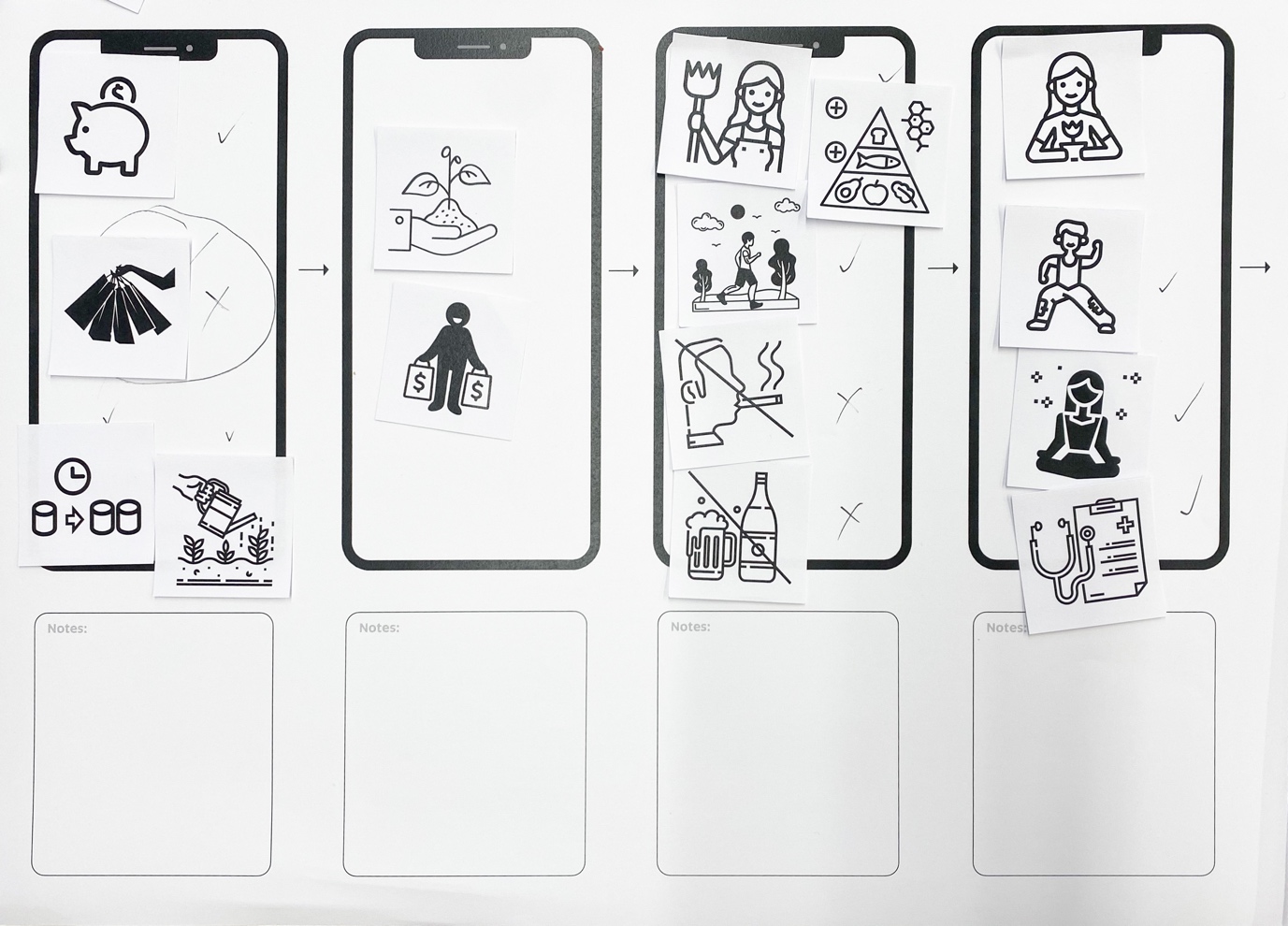


4.1.2

I had financial planning for retirement which seems like it is a pretty well-known concept. Where people are looking at their super and putting a little bit of money aside, trying not to spend excessively and prioritise saving. And then applying this to thinking about health, planning for retirement. Trying to be healthy, cutting out the bad stuff, doing exercise, healthy eating and then that’s going to benefit you when you’re at retirement age that you’ll still be active and mindful and have a clean bill of health. That’s how I’ve mapped it out here.

(What about a shorter time scale, say, a year? Do you think it would make as much sense? Putting money away to do something in a year’s time, 6 months time?)

Yeah, I guess so. If you’re saving money for a holiday, or something like that. What I am currently going through right now, we have signed up to the city to bay and we’ve said we are going to run it but if it was tomorrow, we wouldn’t run it – we would probably collapse. I haven’t really started going through that thinking or training yet, but I know through this that that is how I need to start thinking about it.

(Visualising yourself actually running it.)

That’s one of the prompts I put in. That’s happening in September, so that is my 6 month prompt.

It’s easy when you get your pay to put some money aside but what am I actually doing to make the city to bay outcome happen. That’s the way I need to start thinking about it. Healthy options rather than unhealthy, and making sure I make time for exercise.


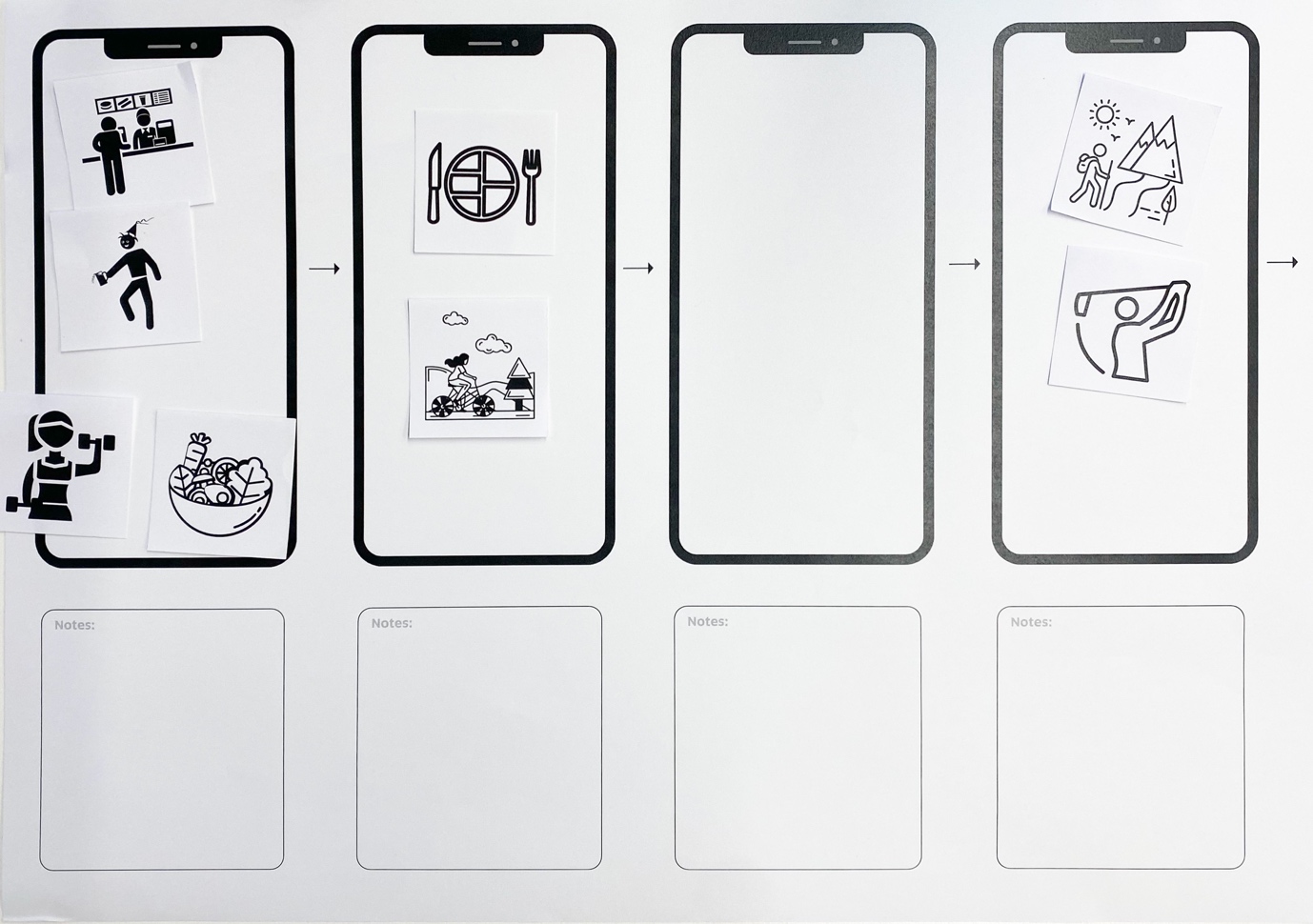


4.1.3

Mine is just three screens. End goal, what makes you happy, trying to identify a goal that you want to work towards. And, can you see yourself doing that at the end? Then taking a step and going in the middle and figuring out the middle steps to connect. It’s just my example with my farm, my childhood, that made me happy. Something I probably want to do when I am older. So what are the middle steps? The middle bit is the hard part to work out, the meat in the sandwich. If you’ve got a goal, that goal you have to figure out at the beginning what that goal is, and if it is for retirement, you want to have a good quality of life. What do you enjoy? What makes you happy?

The light on the hill concept.


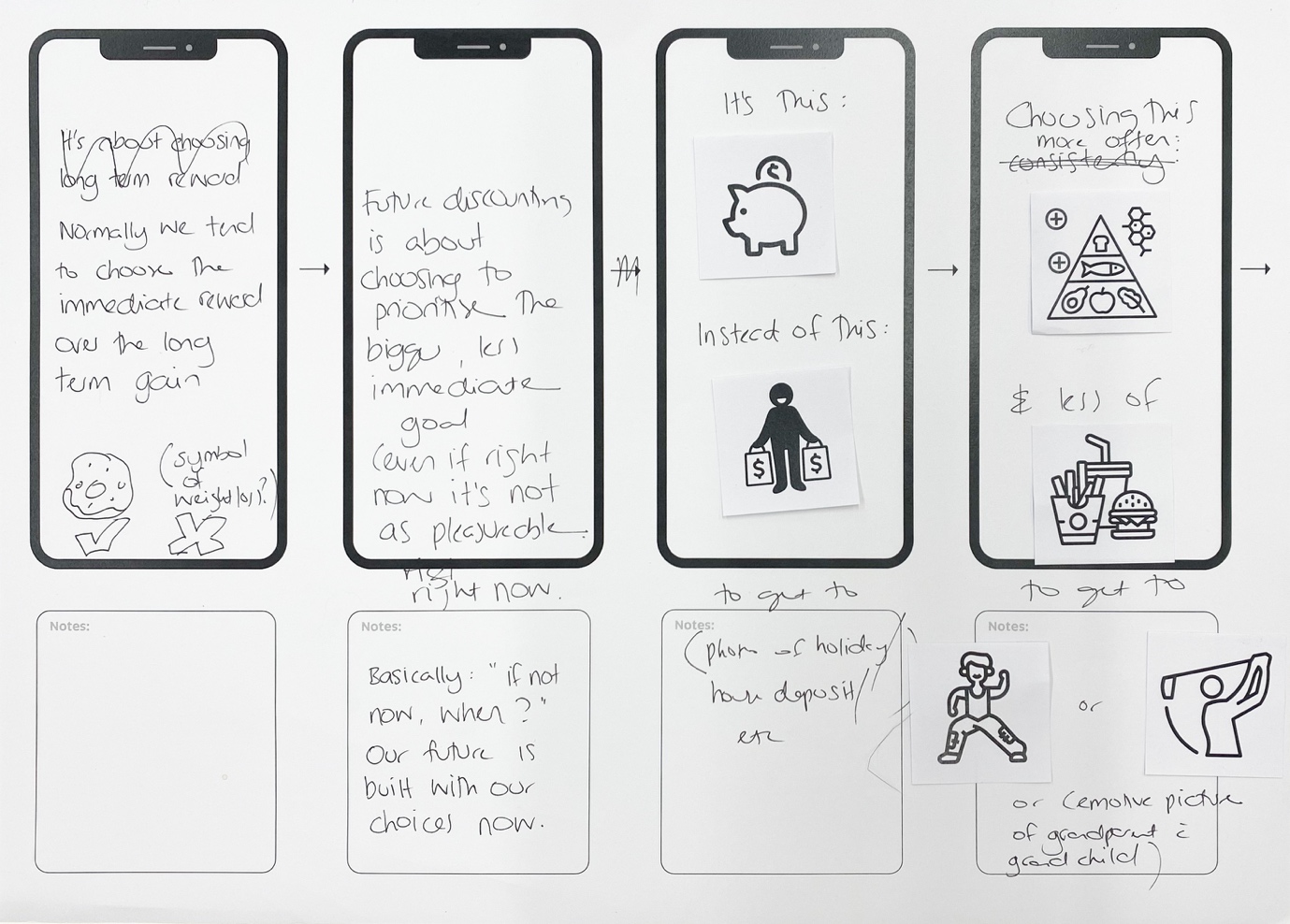


4.1.4

I have a few bits and pieces – but mainly in terms of really simple explanations. Like, saving now, not spending to get to holiday, house deposit, whatever. Three clear steps – this is what you have to do now to get to this thing, because if you don’t have that connection, it’s really hard to understand why you are doing the hard thing immediately.

It boils down to, if not now, when? If you don’t make the choices now, then you are not building the future you want.

It’s what you do now that builds, a year away, two years away, two decades away.

Lots of my friends know the marshmallow test and that is the way I would explain it but for someone who is not familiar with that…I’d say, it is what you do right now that determines future you.
