# Supplementary material for: Digital Episodic Future Thinking Intervention (Luminaut): Co-Design and Iterative Development Study
Source: JMIR Hum Factors. 2026 May 6;13:e74099. doi: 10.2196/74099 (PMC13148339; doi:10.2196/74099)
Supplement: Multimedia Appendix 4 [file humanfactors-v13-e74099-s004.docx]

**Multimedia Appendix 2.** Characteristics of enrolled participants according to workshop attendance.

| **Characteristic** | **Attended Workshop** | **Did Not Attend** | **Total Enrolled** |
| --- | --- | --- | --- |
| Total N | 30 (47.00) | 34 (53.00) | 64 |
| Female, n (%) | 27 (90.00) | 29 (85.29) | 56 (87.50) |
| Age (years), mean (SD)  Age range (years) | 36.37 (5.65)  25-44 | 37.12 (5.63)  25-44 | 36.77 (5.61)  25-44 |
| Education, n (%) |  |  |  |
| Secondary education - Year 12 | 0 (0.00) | 4 (11.76) | 4 (6.25) |
| Certificate III & IV level | 2 (6.67) | 7 (20.59) | 9 (14.06) |
| Advanced diploma/diploma level | 1 (3.33) | 2 (5.88) | 3 (4.69) |
| Bachelor degree level | 11 (36.67) | 7 (20.59) | 18 (28.13) |
| Graduate diploma/certificate level | 7 (23.33) | 3 (8.82) | 10 (15.63) |
| Postgraduate degree level | 9 (30.00) | 11 (32.35) | 20 (31.25) |
| IRSAD quintiles, n (%) |  |  |  |
| 1 (most disadvantaged) | 1 (3.33) | 5 (14.71) | 6 (9.38) |
| 2 | 1 (3.33) | 5 (14.71) | 6 (9.38) |
| 3 | 9 (30.00) | 7 (20.59) | 16 (25.00) |
| 4 | 5 (16.67) | 12 (35.29) | 17 (26.56) |
| 5 (most advantaged) | 14 (43) | 5 (14.71) | 19 (29.69) |
| Fluent in language/s other than English, n (%) | 11 (36.67) | 11 (32.35) | 22 (34.38) |
| Number of Chronic Health Conditions |  |  |  |
| 0 | 14 (46.67) | 16 (47.06) | 30 (46.88) |
| 1 | 12 (40.00) | 13 (38.24) | 25 (39.06) |
| 2 | 2 (6.67) | 5 (14.71) | 7 (10.94) |
| 3 | 2 (6.67) | 0 (0.00) | 2 (3.13) |
| System or device, n (%) |  |  |  |
| Apple | 18 (60.00 | 21 (61.76) | 39 (60.94) |
| Android | 12 (40.00) | 13 (38.24) | 25 (39.06) |

*Note:* n*=*number; SD = standard deviation; IRSAD = Index of Relative Socio-economic Advantage and Disadvantage 2021
