# Supplementary material for: Digital Episodic Future Thinking Intervention (Luminaut): Co-Design and Iterative Development Study
Source: JMIR Hum Factors. 2026 May 6;13:e74099. doi: 10.2196/74099 (PMC13148339; doi:10.2196/74099)
Supplement: Checklist 1 [file humanfactors-v13-e74099-s005.docx]

Checklist 1

GRIPP2-SF (Guidance for Reporting Involvement of Patients and Public Short Form) reporting checklist—short form (Staniszewska et al [66]).

| Section and topic | Item | Section where reported and details |
| --- | --- | --- |
| 1: Aim | Report the aim of PPI (patient and public involvement) in the study | The aim of patient and public involvement (PPI), as well as the aim of the study, is included in the Introduction section. |
| 2: Methods | Provide a clear description of the methods used for PPI in the study | The co-design (or PPI) process was influenced by the British Design Council’s Double Diamond Process, which is an iterative, user-centred and collaborative approach. Further details surrounding the co-design workshops and how data were collected are provided in the Methods. |
| 3: Study Results | Outcomes—Report the results of PPI in the study, including both positive and negative outcomes | The research team used the framework by Braun and Clarke (2012) to perform thematic analysis and code the data into themes. The research team reflected on, discussed, and prioritised specific and conceptual feedback that was derived from the co-design process. These suggestions are reported in the supplementary files and summarised in the results section. |
| 4: Discussion and conclusions | Outcomes—Comment on the extent to which PPI influenced the study overall. Describe positive and negative effects | Examples of where the co-design (or PPI) process influenced the study have been detailed in the Discussion section. For instance, the co-design (or PPI) process was integral to shaping the digital intervention and the results will be used to inform future clinical trials in other ‘pre-risk’ or ‘at-risk’ groups. |
| 5: Reflections / critical perspectives | Comment critically on the study, reflecting on the things that went well  and those that did not, so others can learn | Strengths and limitations of the study are included in the Discussion section. |
